# Comparative Genome Structure, Secondary Metabolite, and Effector Coding Capacity across *Cochliobolus* Pathogens

**DOI:** 10.1371/journal.pgen.1003233

**Published:** 2013-01-24

**Authors:** Bradford J. Condon, Yueqiang Leng, Dongliang Wu, Kathryn E. Bushley, Robin A. Ohm, Robert Otillar, Joel Martin, Wendy Schackwitz, Jane Grimwood, NurAinIzzati MohdZainudin, Chunsheng Xue, Rui Wang, Viola A. Manning, Braham Dhillon, Zheng Jin Tu, Brian J. Steffenson, Asaf Salamov, Hui Sun, Steve Lowry, Kurt LaButti, James Han, Alex Copeland, Erika Lindquist, Kerrie Barry, Jeremy Schmutz, Scott E. Baker, Lynda M. Ciuffetti, Igor V. Grigoriev, Shaobin Zhong, B. Gillian Turgeon

**Affiliations:** 1 Department of Plant Pathology and Plant-Microbe Biology, Cornell University, Ithaca, New York, United States of America; 2 Department of Plant Pathology, North Dakota State University, Fargo, North Dakota, United States of America; 3 Department of Botany and Plant Pathology, Oregon State University, Corvallis, Oregon, United States of America; 4 United States Department of Energy (DOE) Joint Genome Institute (JGI), Walnut Creek, California, United States of America; 5 HudsonAlpha Institute for Biotechnology, Huntsville, Alabama, United States of America; 6 Department of Biology, Faculty of Science, Universiti Putra Malaysia, Serdang, Selangor, Malaysia; 7 College of Plant Protection, Shenyang Agricultural University, Shenyang, China; 8 Department of Forest Sciences, University of British Columbia, Vancouver, Canada; 9 Supercomputing Institute for Advanced Computational Research, University of Minnesota, Minneapolis, Minnesota, United States of America; 10 Department of Plant Pathology, University of Minnesota, St. Paul, Minnesota, United States of America; 11 Pacific Northwest National Laboratory, Richland, Washington, United States of America; University of California San Francisco, United States of America

## Abstract

The genomes of five *Cochliobolus heterostrophus* strains, two *Cochliobolus sativus* strains, three additional *Cochliobolus* species (*Cochliobolus victoriae*, *Cochliobolus carbonum*, *Cochliobolus miyabeanus*), and closely related *Setosphaeria turcica* were sequenced at the Joint Genome Institute (JGI). The datasets were used to identify SNPs between strains and species, unique genomic regions, core secondary metabolism genes, and small secreted protein (SSP) candidate effector encoding genes with a view towards pinpointing structural elements and gene content associated with specificity of these closely related fungi to different cereal hosts. Whole-genome alignment shows that three to five percent of each genome differs between strains of the same species, while a quarter of each genome differs between species. On average, SNP counts among field isolates of the same *C. heterostrophus* species are more than 25× higher than those between inbred lines and 50× lower than SNPs between *Cochliobolus* species. The suites of nonribosomal peptide synthetase (NRPS), polyketide synthase (PKS), and SSP–encoding genes are astoundingly diverse among species but remarkably conserved among isolates of the same species, whether inbred or field strains, except for defining examples that map to unique genomic regions. Functional analysis of several strain-unique PKSs and NRPSs reveal a strong correlation with a role in virulence.

## Author Summary

The filamentous ascomycete genus *Cochliobolus* includes highly aggressive necrotrophic and hemibiotrophic pathogens with particular specificity to their host plants, often associated with production of host selective toxins (HST) that allow necrotrophs to trigger host cell death. Hemibiotrophs must keep their hosts alive during initial infection stages and rely on subverting host defenses by secreting small protein effectors. Many *Cochliobolus* species have emerged rapidly as devastating pathogens due to HSTs. The genomes of *Cochliobolus* and related pathogens that differ in host preference, host specificity, and virulence strategies have been sequenced. Our comparative results, at the whole-genome level, and with a spotlight on core genes for secondary metabolism and small secreted proteins, touch on how pathogens develop and hone these tools, according to host or lifestyle. We suggest that, while necrotrophs and hemibiotrophs employ fundamentally contrasting mechanisms of promoting disease, the tools they utilize (HSTs and protein effectors) overlap. The suites of secondary metabolite and SSP genes that each possesses reflect astounding diversity among species, hinting that gene products, particularly those associated with unique genomic regions, are candidates for pathogenic lifestyle differences. Manipulations of strain-unique secondary metabolite genes associated with host-specific virulence provide tangible examples.

## Introduction

The filamentous ascomycete genus *Cochliobolus* (anamorph *Bipolaris/Curvularia*) is comprised of more than forty closely related, often highly aggressive, pathogenic species with particular specificity to their host plants. All members of the genus known to cause serious crop diseases fall in a tight phylogenetic group suggesting that a progenitor within the genus gave rise, over a relatively short period of time (<20 MYA, Ohm *et al.*, [1]) to the series of distinct species [2], each distinguished by unique pathogenic capability to individual types of cereal (Table 1). Aggressive members include the necrotrophic corn pathogens *Cochliobolus heterostrophus* and *Cochliobolus carbonum*, the oat pathogen, *Cochliobolus victoriae*, the rice pathogen, *Cochliobolus miyabeanus*, the sorghum pathogen, *Bipolaris sorghicola*, the sugarcane pathogen, *Bipolaris sacchari*, and the hemibiotrophic generalized cereal and grass pathogen, *Cochliobolus sativus*.

**Table 1:** *Cochliobolus* and *Setosphaeria-*host interaction biology.

Species^a^	Host/tissue	Disease	HST/Effector?	HST/Effector Target	Pathogen Lifestyle
*Ch* race O (strains C5, Hm540)	corn/leaves	Southern Corn Leaf Blight	?	-	necrotroph
*Ch* race T (strains C4, Hm338, PR1x412)	corn with Tcms^b^/leaves	Southern Corn Leaf Blight	T-toxin	URF13 protein	necrotroph
*Cc* race 1 (strain 26-R-13)	*hm1hm1* c corn/leaves	Northern Leaf Spot	HC-toxin	histone deacetylase	necrotroph
*Cv* (strain FI3)	*Vb* d oats/crown	Victoria Blight	victorin	LOV1	necrotroph
*Cm* (strain WK1C)	rice/leaves	Brown Spot	?	-	necrotroph
*Cs* (strain ND90Pr)	barley, wheat, cereals/leaves	Spot Blotch, Common root rot	?	-	hemibiotroph
*St* (strain 28A)	corn/leaves	Northern Leaf Blight	?	Ht1, Ht2, Ht3, HtN^e^	hemibiotroph

a *Ch = C. heterostrophus*, *Cc = C. carbonum*, *Cv = C. victoriae*, *Cm = C. miyabeanus*, *Cs = C. sativus*, *St = S. turcica*.

b Tcms = cytoplasmic male sterility,

c *hm1hm1* = homozygous recessive for carbonyl reductase,

d *Vb* = presumed to be the same as the *LOV1* e (*Pc-2*) gene for resistance to *P. coronata*.

e Ht1, Ht2, Ht3, HtN are defined as resistance genes based on differential resistance/susceptibility to a set of *S. turcica* races.

All of the known *Cochliobolus* pathogens are classified as necrotrophs, except for *C. sativus*, which, although previously classified as such, has more recently been described as a hemibiotroph [3]. Many necrotrophic *Cochliobolus* spp. and related taxa (e.g., *Pyrenophora tritici-repentis*, *Stagonospora nodorum*) are notorious for their ability to evolve novel, highly virulent, races producing Host Selective Toxins (HSTs) and their concomitant capacity to cause diseases on cereal crops that were bred, inadvertently, for susceptibility to the HST-producing pathogen [4], [5]. For example, in 1970, race T, a previously unseen race of *C. heterostrophus* (*Bipolaris maydis*) caused a major epidemic [Southern Corn Leaf Blight (SCLB)], destroying more than 15% of the maize crop [6]. Before 1970, *C. heterostrophus* was known as an endemic pathogen (race O) of minor economic importance, first described in 1925 [7]. Subsequent research over the ensuing four decades since 1970 has demonstrated that the epidemic was triggered by the unfortunate confluence of complex DNA recombination events in both the fungal pathogen and the plant host. Race T is genetically distinct from race O in that it possesses an extra 1.2 Mb of DNA [8], [9] encoding genes for biosynthesis of the polyketide secondary metabolite, T-toxin, an HST essential for high virulence [4]. These genes are missing in race O. On the plant side, the presence in Texas male sterile cytoplasm (Tcms) maize, of a hybrid mitochondrial gene called *T-urf13*, composed of segments of two mitochondrial and one chloroplast gene, is essential for susceptibility. Tcms corn does not need to be detasseled to prevent self-crossing because it is male sterile, a desirable trait for breeders producing hybrid seed. The resulting popularity of Tcms maize was disastrous from the perspective of pathogen attack, however, as it served as a monoculture of susceptible germplasm [10], [11].

Similarly, *C. victoriae* (*Bipolaris victoriae*), causal agent of Victoria Blight, produces the chlorinated cyclic pentapeptide HST, victorin, rendering it highly virulent to oats carrying the dominant *Vb* allele [12]. The fungus caused widespread destruction (20 states) in the 1940's on oat varieties containing the recently introduced *Pc-2* gene for resistance to crown rust caused by *Puccinia coronata* [13]. Like the *C. heterostrophus* T-toxin/Tcms case, the monoculture of Victoria oats carrying *Pc-2* was the perfect milieu for attack by *C. victoriae* producing victorin, which elicits *Pc-2*-dependent Programmed Cell Death (PCD). Recent work with Arabidopsis identified a resistance-like protein responsible for susceptibility to *C. victoriae* and victorin [14], [15]. This work is seminal in demonstrating fungal HSTs can target resistance proteins to promote disease.

In contrast to the dominant plant host genes required for susceptibility to *C. heterostrophus* and *C. victoriae*, susceptibility to Northern Corn Leaf Spot caused by *C. carbonum* (*Bipolaris zeicola*) is conferred by a homozygous recessive maize gene(s) [16], [17]. *C. carbonum* race 1 produces the cyclic-tetrapeptide HST, HC-toxin, which is specifically active against corn with the genotype *hmhm*, as is the fungus itself [4], [18], [19]. *Hm1* and *Hm2* encode carbonyl reductases which inactivate the toxin [16]; *hmhm* lines, cannot inactivate the toxin, and are therefore sensitive. The site of action of HC-toxin in susceptible corn is histone deacetylase; it is hypothesized that HC-toxin acts to promote infection of maize of genotype *hm1hm1* by inhibiting this enzyme, resulting in accumulation of hyperacetylated core (nucleosomal) histones. This then alters expression of genes encoding regulatory proteins involved in plant defense [20], [21]. *C. carbonum* races 2 and 3 do not produce the toxin.

*C. miyabeanus* (*Bipolaris oryzae*) is the causal agent of Brown Spot of rice which contributed, along with a cyclone and tidal waves, to the Bengal rice famine of 1942/1943 that resulted in starvation of more than two million people [22]. To date, no HST has been associated with virulence, although *C. miyabeanus* culture filtrates can suppress plant phenol production [23].

*C. sativus* (*Bipolaris sorokiniana*) causes diseases of roots (Common Root Rot), leaves (Spot Blotch), and spikes (known as black point or kernel blight) of cereals (mainly barley and wheat) [24], [25], but also attacks many grasses, including switch grass (*Panicum virgatum* L.) [3], [26], [27] and *Brachypodium distachyon* (S. Zhong, unpublished). Three *C. sativus* pathotypes (0, 1 and 2) have been described [28], based on differential virulence patterns on three barley genotypes (ND5883, Bowman, and NDB112). Pathotype 0 isolates show low virulence on all three barley genotypes. Pathotype 1 isolates show high virulence on ND5883 but low virulence on other barley genotypes. Pathotype 2 isolates show high virulence on Bowman but low virulence on ND5883 and NDB112. Genetic analysis and molecular mapping indicates that a single locus, *VHv1*, controls high virulence of the pathotype 2 isolate ND90Pr on Bowman [29], [30], however, the exact nature of the gene(s) was unknown before this study (see Results). More recently, a new pathotype, highly virulent on NDB112, the most durable spot blotch resistance source in barley [31], has been found in North Dakota [32] and Canada [33].

*Setosphaeria turcica* (*Exserohlium turcicum*, *Helminthosporium turcicum*), a hemibiotrophic vascular leaf pathogen, is a member of the closest genus to *Cochliobolus* (see Figure 1 in Ohm *et al.*, [1]), and causes Northern Leaf Blight (NLB), a major disease of maize and sorghum in the US and internationally [34]. To date, at least four races of *S. turcica* have been identified based on their differential virulence performance on maize carrying resistance genes known as *Ht* [35], [36]. In recent work, Martin et al. [34], identified new resistance genes (named *St*) in both maize and sorghum. The connections between the *Ht* and *St* resistance genes are unclear at this point.

Until recently, it was assumed that necrotrophs use brute force methods (e.g., arsenals of cell wall degrading enzymes, HSTs) to invade and kill host tissues and do not subtly manipulate the host during infection, as do biotrophs with their arsenal of effectors [37]. Several lines of evidence, from studies of the Dothideomycete, necrotrophic wheat pathogens, *Pyrenophora tritici-repentis* [38] and *Stagonospora nodorum*, [39] indicate that, like biotrophs, these pathogens secrete protein effectors (in this case also called HSTs) that interact with host targets in a gene-for-gene manner. Unlike biotrophs, however, interaction of the fungal effector and host protein results in *susceptibility*, rather than resistance. The above-mentioned research on *C. victoriae*, further indicates mechanistic overlap of pathogenic lifestyle and has major implications regarding the challenge plants face in defending themselves against both necrotrophs and biotrophs [37], [40]. Recent studies involving *Arabidopsis* have revealed that victorin-induced PCD requires a host NB-LRR-type resistance protein [15], [41]. Thus a protein with canonical resistance protein structure is required for *susceptibility*. These observations point toward victorin, subverting effector triggered defenses against biotrophs, such as *P. coronata* (see above), to promote susceptibility to a necrotroph.

Here we provide a comparative analysis of genome similarities and differences among *Cochliobolus* and *Setosphaeria* pathogens, with particular emphasis on strain and species-unique sequences, secondary metabolism genes, and genes encoding small secreted proteins. Identification of these key structural genomic and molecular differences is the first step in understanding species-specificity and how closely related necrotrophic and hemibiotrophic pathogens cause disease. As proof of concept, we offer several examples of how comparative approaches pinpoint virulence associated, species-specific regions of interest. The Joint Genome Institute (JGI) has sequenced five strains of *C. heterostrophus* (three race T and two race O strains) and four additional species of *Cochliobolus*, including *C. carbonum*, *C. victoriae*, *C. miyabeanus*, and *C. sativus* and one strain of *S. turcica*. Add to this that host genome sequences (corn, rice, barley and *B. distachyon*) for six of these pathogens are available and one has the information base for dissecting both sides of the interaction mechanism going forward.

## Results

### Genome statistics

Five strains of *C. heterostrophus*, one strain of *C. sativus*, and one strain each of *C. victoriae*, *C. carbonum*, *C. miyabeanus*, and *S. turcica* were sequenced by JGI (Table 2, Table 3). Two *C. heterostrophus* strains and one strain each of *C. sativus* and *S. turcica* were fully sequenced as described in Materials and Methods, while the remaining genomes were sequenced using Illumina and assembled *de novo* using Velvet, as described in Materials and Methods. The highly inbred race O lab strain C5 was used as the reference sequence for all comparisons, as it is the most complete, consisting of only 68 scaffolds.

**Table 2:** *C. heterostrophus* race O strain C5, race T strain C4, *C. sativus* and *S. turcica* genome statistics.

Genome Characteristic	Sequenced strain			
	*C. heterostrophus* strain C5	*C. heterostrophus* strain C4	*C. sativus* strain ND90Pr	*S. turcica* strain 28A
Genome sequence total (Mb)	36.46	32.93	34.42	43.01
Contig sequence total (Mb)	36.32	32.09	33.22	38.26
Genome scaffold count	68	207	157	407
Genome contig count	88	586	478	1951
Scaffold N/L50	7/1.84 Mb	13/0.96 Mb	7/1.79 Mb	8/2.14 Mb
Contig N/L50	12/1.17 Mb	55/0.18 Mb	43/0.24 Mb	210/0.05 Mb
% genome covered by repeats	9%	1%	6%	12.96%
# predicted genes	13,336	12,720	12,250	11,702

**Table 3:** Statistics for short read re-sequenced Cochliobolus genomes.^a^

Strain	Sequencing depth	# Reads	% reads mapped	% paired	Read length (bp)	# Assembled nodes	Velvet assembly size (bp)
*Ch* Hm540	35.2	37,796,798	95	94	35	4,387	32,575,369
*Ch* Hm338	79.55	37,923,292	97	98	76	5,227	33,353,785
*Ch* PR1x412	33.58	36,292,318	94	95	35	7,229	32,661,792
*Cv* FI3	30.41	81,216,986	19	92	76	4,525	33,638,943
*Cc* 26-R-13	33.05	83,423,746	20	90	76	3,671	32,298,417
*Cm* WK1C ATCC 44560	27.39	81,110,386	17	90	76	3,553	32,695,608

a Using Illumina technology.

*Ch = C. heterostrophus*, *Cv = c. victoriae*, *Cc = C. carbonum*, *Cm = C. miyabeanus*.

Overall sequence assembly and annotation statistics are presented in Table 2 and Table 3. All *Cochliobolus* genomes are in the 32–38 Mb range with an estimated gene content of 11,700–13,200. The *S. turcica* genome is ∼43 Mb with ∼11,700 genes. Overall gene content and genome organization are highly similar within this group of fungi. In contrast, comparative analysis of *C. heterotrophus*, *C. sativus* and *S. turcica* in the context of 14 more distantly related Dothideomycetes genomes described elsewhere (Ohm *et. al.*, [1]) revealed significant variation.

### Mapping scaffolds to the genetic maps

**Figure 1:**
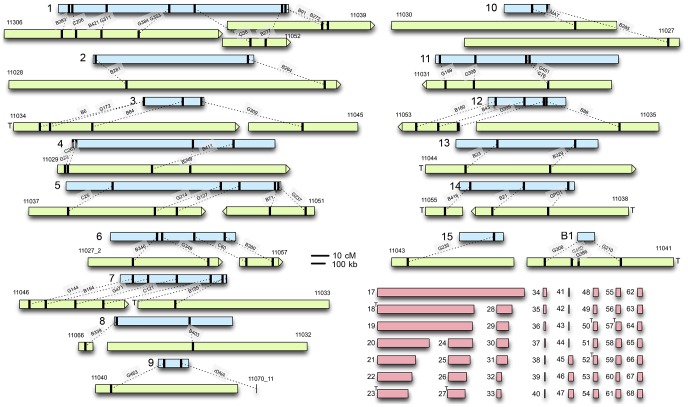
*C. heterostrophus* RFLP sequences anchor sequenced scaffolds to the genetic map. Genetic linkage groups determined by Tzeng *et al.* [8], are in light blue (linkage group numbers on the left). Assembled scaffolds from the reference C5 strain that could be anchored to each linkage group are in light green; numbers above are internal JGI identifiers (Table S2). Black bars indicate the relative locations of RFLPs Tzeng et al. [8] or feature, linked by dotted lines. The relative scale of genetic/physical distance was determined by calculating the average genetic/physical distance between consecutively placed RFLP markers (Figure S1). Scaffold ends marked with a T contain telomeric sequence. Unplaced scaffolds are shown in pink. Maps are to scale and shown as centiMorgans (cM) or kilobases (kb).

*C. heterostrophus*: A genetic map with 125 RFLP markers was constructed previously [8] using *C. heterostrophus* race O field strain Hm540 (sequenced herein) and race T C-strain B30.A3.R.45 (same backcross series as strains C5 and C4, sequenced herein [42], [43]) as parents. RFLP sequences were used to refine the *C. heterostrophus* race O strain C5 physical assembly and link it to the genetic RFLP map (Figure 1, Text S1). The interconnected genetic and physical maps allowed comparisons of physical and genetic distance, which was found to be ∼13 kb/cM on average (Figure S1). Correlations were also made between previously estimated chromosome sizes based on CHEF gel analysis [9], and physical size based on sequence assemblies (Tables S1, S2).

Based on RFLP map data, parental field isolate Hm540 was reported to lack the dispensable chromosome present in the other parental strain (B30.A3.R.45) used to build the map [8]. Comparisons of all five sequenced *C. heterostrophus* strains, using the Mauve alignment tool [44], supported this observation and revealed that all sequenced strains carried the previously recognized ‘B’ or dispensable chromosome (which corresponds to strain C5 scaffold 16) (Figure S2). Race O chromosomes 6 and 12 are of interest because counterparts are reciprocally translocated in race T and the high-virulence conferring *Tox1* locus, encoding genes for biosynthesis of T-toxin, maps genetically to both breakpoints [9]. Comparison of the race O and race T assemblies provided some clues as to the physical locations of these breakpoints, but the exact positions remain elusive, due to structural complexity associated with these regions [45]. Additional details regarding linkage of the *C. heterostrophus* physical assembly to the genetic map are available in Text S1.

*C. sativus*: Before mapping sequenced scaffolds to the previously constructed *C. sativus* genetic map [30] 121 polymorphic simple sequence repeat (SSR) markers were identified in the assembly sequences of the ND90Pr and ND93-1 parents, as described in Materials and Methods. Then, sequences of the SSRs and other markers were used to assign sequenced scaffolds to the updated map. Thirty of these linkage groups contained SSR markers and were found to be associated with 16 scaffolds, summing to 29.32 Mb. Seven linkage groups were unassigned (Figure S3). The two AFLP marker sequences (E-AG/M-CG-121 and E-AG/M-CA-207), cosegregating with the *VHv1-*associated high virulence of *C. sativus* pathotype 2 on cultivar Bowman [30], were used as blast queries against the ND90Pr genome assembly. E-AG/M-CG-121 mapped to scaffold 5, while E-AG/M-CA-207 mapped to scaffold 40 (Figure S3). Details of the construction, linking, and analysis of the *C. sativus* map are available in Text S1.

### Single nucleotide polymorphisms (SNPs) between the reference *C. heterostrophus* C5 strain and other strains

#### SNPs in *C. heterostrophus* strains

Genome assemblies were aligned, pairwise, to the *C. heterostrophus* race O C5 reference using MUMmer [46] to analyze SNP frequencies between strains and species and to infer whole-genome similarity. *C. heterostrophus* race T strain C4, which belongs to the same inbred laboratory strain series as the C5 reference [42] contained 1,584 SNPs (Table 4, Table S3). In contrast, the three *C. heterostrophus* field strains, race O strain Hm540, race T strains Hm338, and PR1x412 had 50,864, 30,624, and 33,552 SNPs, respectively, (∼20–30× more than strain C4) (Table 4, Table S3). More SNPs were identified for Hm540, the only race O field isolate, than for the race T field strains Hm338 and PR1x412, despite the reference C5 strain being race O. This supports previous RFLP data which indicated that race O field strain Hm540 was more diverse than any field isolate examined [8] and the hypothesis that race T arose once and more recently than race O [9], [47], [48].

**Table 4:** SNPs between *C. heterostrophus* (*Ch*) strain C5 and other strains and species.

Isolate	# Scaffolds	# Aligned	Total bases	Aligned bases	Total SNPs	Aligned bp/SNP
*Ch*C4	207	173	32,929,167	31,789,127	1,584	20,068.9
*Ch*Hm338	2,711	1,988	33,353,785	32,483,575	30,624	1,060.7
*Ch*Hm540	4,387	1,954	32,575,369	30,971,859	50,864	608.9
*Ch*PR1x412	7,229	4,269	32,661,792	31,041,698	33,552	925.2
*C. victoriae*	1,207	522	33,659,279	24,967,357	2,083,899	12
*C. carbonum*	3,671	1096	32,298,417	24,784,636	2,059,993	12
*C. miyabeanus*	3,553	891	32,878,767	24,421,161	2,110,786	11.6
*C. sativus*	157	70	34,417,436	25,730,439	1,981,616	13
*S. turcica*	407	179	43,014,577	6,932,055	749,006	9.3

For all *C. heterostrophus* strains, 30–31.5 Mb (95–97%) of the assembled basepairs (bp) could be aligned, although only 40–80% of the scaffolds could be aligned. Thus, 3–5% of each genome could not be aligned and corresponding sequences were located on small, difficult to assemble contigs.

#### SNPs in *C. sativus* strains

For the two *C. sativus* genomes, ND90Pr and ND93-1, 86.6% of the higher quality ND90Pr genome could be aligned to 96.6% of the ND93-1 genome, yielding 60,448 SNPs (Table 5). The relative similarity between these two strains is comparable to that seen between *C. heterostrophus* strains.

**Table 5:** SNPs between *C. sativus* strains ND90Pr and ND93-1.

Isolate	# Scaffolds	# Aligned	Total bases	Aligned bases	Total SNPs	Aligned bp/SNP
ND90Pr	157	156	34,417,436	29,889,643	60,448	494
ND93-1	18,112	17,418	30,342,250	29,309,584	60,448	485

#### SNPs in *Cochliobolus* species

*C. victoriae*, *C. carbonum*, *C. miyabeanus*, and *C. sativus* species had 2,083,899, 2,059,993, 2,110,786, and 1,981,616 SNPs, respectively, when aligned to *C. heterostrophus* reference strain C5, ∼54× fold more than comparisons within species (Table 4, Table S3). In each case, 24–25 Mb (75%) of assembled bps could be aligned to the reference, indicating that, in addition to the much higher quantity of SNPs than between *C. heterostrophus* strains, a full quarter of each genome was not present in *C. heterostrophus*. Although *C. sativus* has a wider host range than any of the other species, there is no compelling evidence that it is less related to *C. heterostrophus* than the other species. All species are estimated to have arisen relatively recently (<20MYA ago, see Figure 1 in Ohm *et al.*, [1]). Analysis of the mating type (*MAT*) regions (Text S1, Figure S4, Table S4) yielded similar patterns, except that within the *C. heterostrophus* species, the most similar *MAT* flanking regions were those of the same mating type, regardless of whether the strain was race O or race T, or an inbred or field strain. *C. carbonum* and *C. victoriae* are capable of crossing with each other [48] and thus would be predicted to be closely related and to have fewer SNPs between them than between either of them and other species. Indeed, when *C. victoriae* was aligned to *C. carbonum*, 292,216 SNPs were identified, roughly 10-fold fewer than when *Cochliobolus* species are compared to the *C. heterostrophus* reference, yet 10-fold more than are present when *C. heterostrophus* field strains are compared to the *C. heterostrophus* C5 reference strain (Table 6, Table S3). Thus, the SNP data are in line with the notion that *C. victoriae* and *C. carbonum* are closely related and that *C. victoriae* may have arisen from a *C. carbonum* isolate [49]. For comparison, aligning *C. miyabeanus* or *C. sativus* to the *C. carbonum* genome yields 2,078,277 and 2,022,068 SNPs, respectively (Table 6, Table S3), in the range seen when comparisons are made with *C. miyabeanus*, *C. carbonum*, *C. victoriae*, or *C. sativus* to *C. heterostrophus* C5 (Table 4, Table S3).

**Table 6:** SNPs between *C. carbonum* and other species.

Isolate	# Scaffolds	# Aligned	Total bases	Aligned bases	Total SNPs	Aligned bp/SNP
*C. victoriae*	1,207	565	33,659,279	30,153,232	292,216	115.2
*C. miyabeanus*	3,553	1,008	32,878,767	25,518,991	2,078,277	12.3
*C. sativus*	157	96	34,417,436	26,024,810	2,022,068	12.9
*Ch*C5	68	67	36,456,735	27,650,456	2,094,062	13.2
*S. turcica*	407	329	43,014,577	7,595,377	747,875	10.2

SNP calls generated in all genome comparisons were inflated for A to G, T to C, C to T, and G to A transitions, the most common type of mutation. In all comparisons made, including C5 to C4, these transitions were 5–10 times more abundant than other changes (Table S3).

#### SNPs between *C. heterostrophus* and *S. turcica*

We could align only 16.12% (6.9 Mb) of the *S. turcica* genome to the *C. heterostrophus* reference genome, as indicated in Table 4. Full details are available in Table S3.

#### Summary

Inbred *C. heterostrophus* C4 strain has far fewer SNPs (1,584), than *C. heterostrophus* field strains, when compared to the *C. heterostrophus* C5 reference. The numbers of SNPs in the *C. heterostrophus* field strains are comparable to each other, with Hm540, a race O isolate, containing the most (50,864) when compared to race O *C. heterostrophus* C5. The largest numbers of SNPs are between *C. heterostrophus* C5 and the additional *Cochliobolus* species (1.9–2.1 million) and these are ∼54 fold higher than within species SNPs (Table 4, Table S3). The *C. sativus* strains are as similar to each other as different *C. heterostrophus* strains are to one another, while *C. carbonum* and *C. victoriae* demonstrate a level of relatedness in between that seen among isolates within a species and across species.

### Identification of strain- and species-specific genomic regions

**Figure 2:**
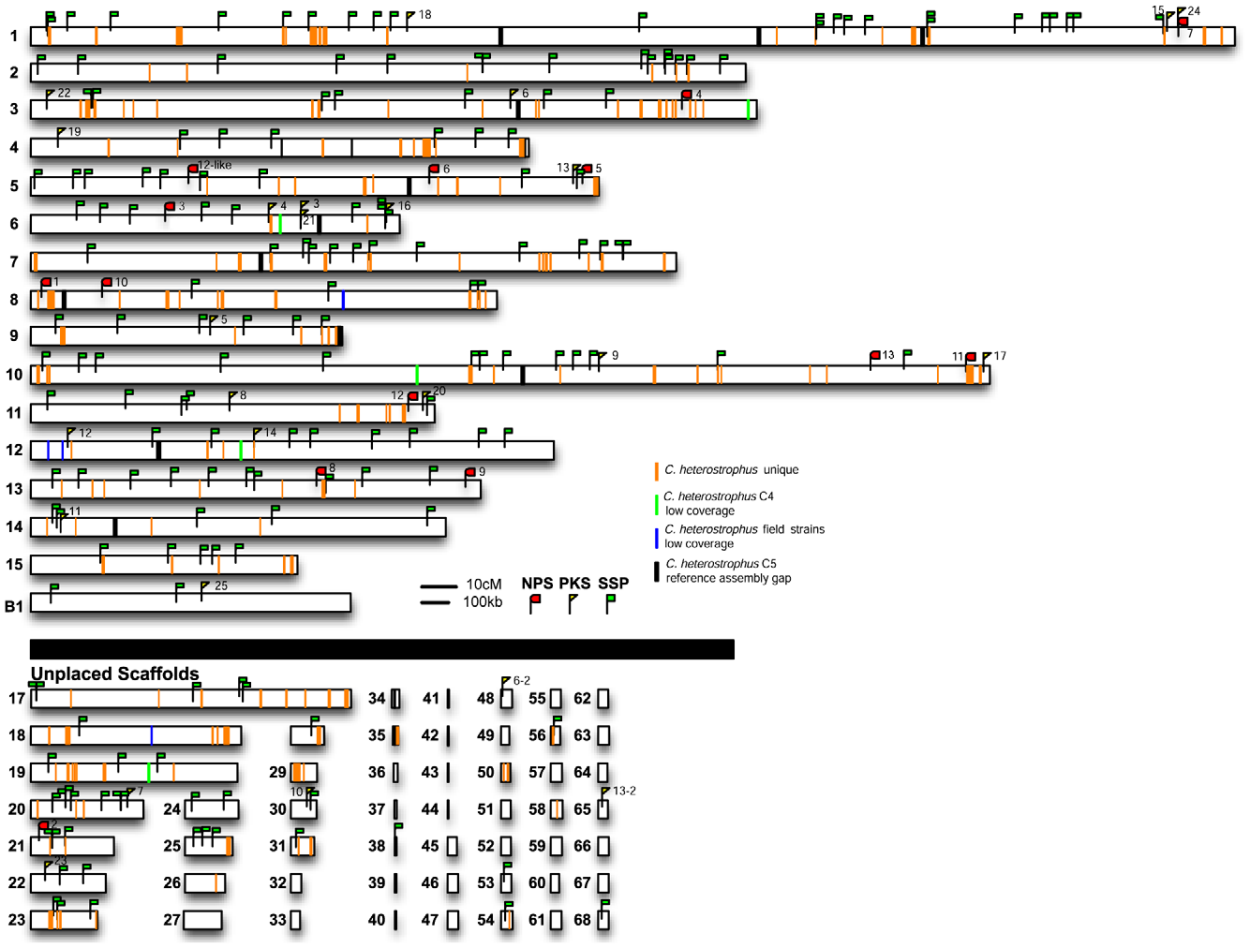
*C. heterostrophus* unique regions, secondary metabolite genes, and small secreted protein encoding genes are distributed throughout the genome. Reads from each *C. heterostrophus* or *Cochliobolus* species isolate were aligned to the *C. heterostrophus* C5 reference genome and regions of low coverage mapped; orange lines indicate no match in all non *C. heterostrophus* species but conserved in *C. heterostrophus* isolates (*C. heterostrophus* unique), green lines indicate low coverage of *C. heterostrophus* strain C4 reads, blue lines indicate low coverage of all *C. heterostrophus* field isolates. Gaps in the assembly are indicated as black vertical bars. The locations of all *C. heterostrophus* strain C5 *NPS* (red flags), *PKS* (yellow flags), and SSP (green flags) genes were also mapped to the C5 reference assembly. Note that one or more *NPS* or *PKS* genes map to most linkage groups and that several map to unplaced scaffolds. SSPs map to every chromosome/linkage group and most unplaced scaffolds. Same scale as Figure 1.

To begin to identify regions in the *C. heterostrophus* C5 assembly not represented in other strains, we first mapped gaps in the reference assembly (thick vertical black bars, Figure 2). A single gap was present in the assembly of 9 of 16 chromosomes and we speculate that these gaps correspond to centromeric regions. We then mapped sequence reads of all *Cochliobolus* genomes in this study to the *C. heterostrophus* C5 reference, identifying regions in the C5 reference that were not present in the query genome (Table S5). The sets of C5 genomic regions that were absent in a given query were combined to determine genomic regions unique and/or conserved at different taxonomic levels.

Individual reference-unique region counts were recorded for each of the *C. heterostrophus* strains. There were 609 areas of the C5 assembly unique (C4 reads did not map there) to the C5 genome when C4 reads were mapped to it. For *C. heterostrophus* strains Hm540, PR1x412, and Hm338, there were 3,279, 4,383 and 1,480 such regions, respectively. When only regions greater than 5,000 bp were considered, there were 19 when C4 was used as the query, and 33, 75, and 30, when PR1x412, Hm540, and Hm338 were used as the queries, respectively. Many of the gaps associated with Hm540 mapped to reference C5 scaffold 16 corresponding to the dispensable B chromosome, which is absent in Hm540 (Figure S2).

The C5 reference-unique regions were then combined and filtered to identify conserved genomic regions at the strain or species level, where regions were unique in one type of comparison, but not in others (Table S5). We designated “inbred C strain”-specific regions as gaps found when all field strains were aligned to C5 but not when C4 was aligned to C5, race O specific regions as gaps found when all race T strains were aligned to C5 but not when race O strain Hm540 was aligned to race O strain C5, and *C. heterostrophus*-specific regions as gaps found when all *Cochliobolus* species were aligned to C5, but not when any *C. heterostrophus* strain was aligned.

A total of 28,556 bp is missing from all *C. heterostrophus* field strains, yet present in the C4 assembly (Table S5). This ∼30 kb of DNA represents sequence uniquely conserved in the inbred C strains. There are at least six fungal-specific Zn2Cys6 transcription factors (ID# 1019013, 1020538, 1021058, 1021066, 1100349, 1100899) present in this C strain unique cache that may signify “early action” strain diversification. Zn2Cys6 transcription factors were among the most abundant predicted domains in the *C. heterostrophus* gene catalogue [1].

There were almost no race O specific regions identified (sequence found only in C5 and Hm540); only 11 regions, summing to 4,309 bp, were identified (Table S5). Our working hypothesis is that the essential difference between race O and race T is the 1.2 Mb of *Tox1* race T DNA (not in race O C5 and therefore not able to be aligned). Both race O only regions (scaffold 12, 732532–734880, and scaffold 19, 441888–443521) contain a single protein each (ID# 59063, 34937) with no conserved domains or predicted function.

Most significantly, at the species level, a total of 11.76 Mb DNA was missing from all non-*C. heterostrophus Cochliobolus* genomes analyzed, yet found in all field strains of *C. heterostrophus*. Only 1.6 Mb of this was found in pieces larger than 5 kb. Most of the sequence that separates *C. heterostrophus* from other species, therefore, is not the result of large wholesale insertions or deletions of DNA, but from a more piecewise gain and loss.

#### Summary

By mapping query reads to the reference *C. heterostrophus* C5 assembly, we generated relative coverage maps for each strain and species. Intersecting these coverage maps as present or absent in different genome sets gives us insight into short-term strain evolution. 28,556 bp of sequence was found in both of the *C. heterostrophus* C strains, but none of the *C. heterostrophus* field strains, while only 4,309 bp was uniquely conserved in race O strains and 11.7 Mb was uniquely conserved in *C. heterostrophus*. This sequence is what makes *Cochliobolus heterostrophus* unique from other *Cochliobolus* species.

### Secondary metabolism: Nonribosomal peptide synthetases

Nonribosomal peptide synthetases (NRPSs), found in fungi and bacteria, are multimodular megasynthases that catalyze biosynthesis of small bioactive peptides (NRPs), including virulence determinants, such as HSTs, via a thiotemplate mechanism independent of ribosomes [50], [51], [52], [53], [54]. NRPSs synthesize peptides using sets of core domains, known as modules, which consists of three domains: 1) an adenylation (AMP) domain which recognizes and activates the substrate via adenylation with ATP, 2) a thiolation (T) or peptidyl carrier protein (PCP) domain which binds the activated substrate to a 4′- phosphopantetheine (PP) cofactor via a thioester bond and transfers the substrate to 3) a condensation (C) domain which catalyzes peptide bond formation between adjacent substrates on the megasynthase complex. NRPSs can be mono-, bi-, or multi-modular and core domains in any particular multimodular enzyme may be most closely related to one another or to a domain from a different NRPS.

The suites of NRPS encoding genes (*NPS*) in the *C. heterostrophus* C4 and C5 genomes were identified and annotated previously [55], [56], [57]. To address degree of conservation and evolutionary relationships of NRPS proteins in our subject species in order to make inferences about function, we used the fungal AMP-binding (AMP) domain Hidden Markov Model (HMM) developed by Bushley and Turgeon [57] to identify individual AMP domains in the additional strains of *C. heterostrophus* and other *Cochliobolus* species, plus *S. turcica*. Phylogenetic trees were built to develop a comparative NRPS AMP domain inventory and included the known *C. heterostrophus* C5 AMP domains as a reference (Table S6).

#### Conservation of known *C. heterostrophus* NRPSs

**Figure 3:**
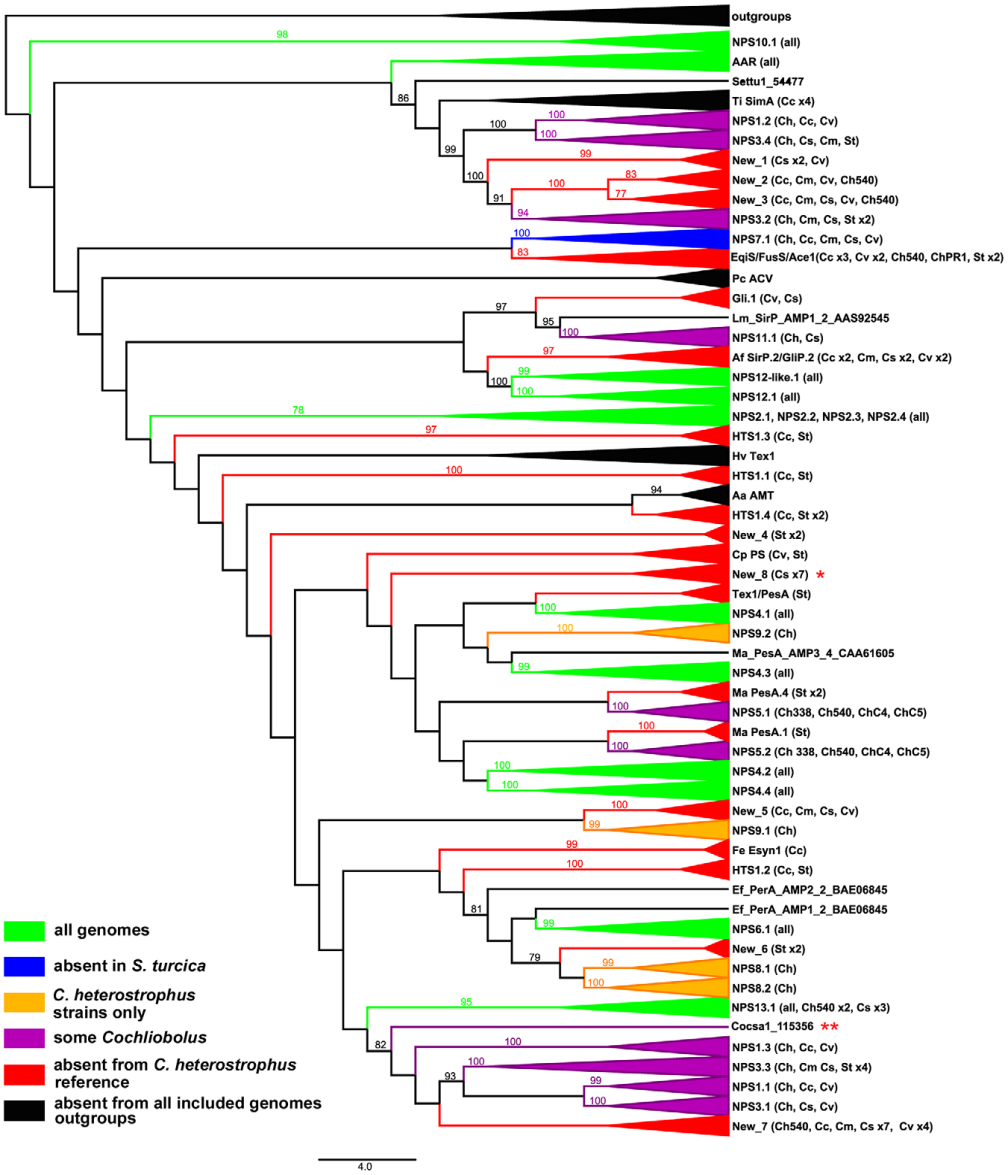
Cartoon of cross-species phylogenomic analyses of individual AMP binding domains from NRPS proteins. NRPS AMP domains were extracted from all five *C. heterostrophus* and from the *C. victoriae*, *C. carbonum*, *C. miyabeanus*, *C. sativus*, and *S. turcica* genomes. Members of the reference set of previously annotated *C. heterostrophus* NRPS AMP domains [57] were used as benchmarks for branches. Branches of the full Augustus phylogenetic tree (Figure S5A) are collapsed according to clustering with the reference set of *C. heterostrophus* AMP domains. Presence in each of the five *C. heterostrophus* strains (ChC5, ChC4, Ch540, Ch338, ChPR1), *Cochliobolus* species [*C. victoriae* (Cv), *C. carbonum* (Cc), *C. miyabeanus* (Cm), *C. sativus* (Cs)], or *S. turcica* (St) is noted in parentheses. Designations such as ‘St x2’ indicate that two AMP domains from *S. turcica* are present. AMP domains not grouping with the previously annotated *C. heterostrophus* set are labeled as ‘New_1 through _8’. Groups are color-coded according to their distribution in the genomes examined: green = present in all genomes, blue = absent from *S. turcica* only, orange = exclusive to *C. heterostrophus* and present in all *C. heterostrophus* strains, purple = present discontinuously in some, but not all *Cochliobolus* genomes, red = absent from the *C. heterostrophus* C5 reference, and black = absent from all included genomes. The latter includes outgroups and NRPS AMPs in species other than *Cochliobolus* or *Setosphaeria* producing some well-known metabolites [e.g., *Alternaria alternata* AMT producing AM-toxin, (AMT)]. Bootstrap values 75% and above are indicated on the branches. Branch lengths represent the average number of substitutions per site. Asterisks indicate unique *C. sativus* NRPSs discussed in text (Table 8, Figure 7).

First, using the highly curated set of *C. heterostrophus* NRPSs (Table 7, left column), we determined if all 28 AMP domains of all 14 *C. heterostrophus* C5 reference NRPS proteins were present in each genome analyzed. For the *C. heterostrophus* genomes, all C5 reference AMP domains and thus all complete NRPS proteins were present (Table 7, Figure 3, for full phylogenetic trees see Figure S5A, S5B, and for master inventories, see Table S6), with the single exception of bimodular NPS5, which was absent from race T strain PR1x412. Thus, there is almost complete conservation of NRPSs at the species level.

**Table 7:** Conservation of *C. heterostrophus* strain C5 nonribosomal peptide synthetases in other *Cochliobolus* strains and species.

C5	C4 Genbank accession	JGI ID	In other *C. heterostrophus* strains?	In other *Cochliobolus* spp.?	In *S. turcica*?
NPS1.1	AAX09983	1101207	+	*Cv*, *Cc*	−
NPS1.2	AAX09983	1101207	+	*Cv*, *Cc*	−
NPS1.3	AAX09983	1101207	+	*Cv*, *Cc*	−
NPS2.1	AAX09984	128084	+	+	+
NPS2.2	AAX09984	128084	+	+	+
NPS2.3	AAX09984	128084	+	+	+
NPS2.4	AAX09984	128084	+	+	+
NPS3.1	AAX09985	128098	+	*Cm*, *Cs*	−
NPS3.2	AAX09985	128098	+	*Cm*, *Cs*	+
NPS3.3	AAX09985	128098	+	*Cm*, *Cs*	+
NPS3.4	AAX09985	128098	+	*Cm*, *Cs*	+
NPS4.1	AAX09986	1091637	+	+	+
NPS4.2	AAX09986	1091637	+	+	+
NPS4.3	AAX09986	1091637	+	+	+
NPS4.4	AAX09986	1091637	+	+	+
NPS5.1	AAX09987	1095362	not in PR1x412	−	−
NPS5.2	AAX09987	1095362	not in PR1x412	−	−
NPS6.1	AAX09988	128080	+	+	+
NPS7.1	AAX09989	1209664	+	+	−
NPS8.1	AAX09990	1227314	+	−	−
NPS8.2	AAX09990	1227314	+	*Cs*	−
NPS9.1	AAX09991	1159473	+	−	−
NPS9.2	AAX09991	1159473	+	−	−
NPS10.1	AAX09992	1175121	+	+	+
NPS11.1	AAX09993	1104551	+	*Cs*	−
NPS12.1 (116719)	AAX09994	116719	+	+	+
NPS12.1 (118012)	-	1223123	+	+	+
NPS13	AY884198	1215193	+	−	−

+ = present, − = absent; 2x = two copies.

Strains *Ch* = *C. heterostrophus*, Hm540, PR1x412 are *C. heterostrophus* strains, *Cs = C. sativus*, *Cv = C. victoriae*, *Cc = C. carbonum*, *Cm = C. miyabeanus*, *St = S. turcica*,

See Figure S5A and Table S6.

**Figure 4:**
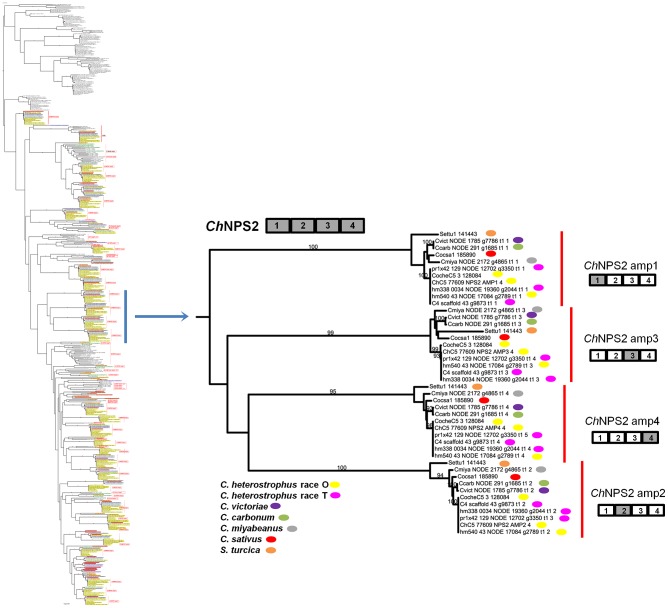
NPS2 is an example of a highly conserved Dothideomycete NRPS. NPS2 consists of four AMP domains (cartoon above partial tree) that produce the hexapeptide intracellular siderophore, ferricrocin, responsible for iron storage within cells. NPS2 is present in all five strains of *C. heterostrophus*, all other *Cochliobolus* species and *Setosphaeria*. Note four branches, which cluster together on the thumbnail of the full tree (Figure S5A), each corresponding to one of the four NPS2 AMP domains (gray AMP in NRPS cartoon to the right). Each fungal race or strain is color coded as indicated. Reference C5 AMP indicated as e.g., ChC5 77609 NPS2 AMP1 4; 77607 is the Genbank protein ID, NPS2 is the NRPS designation, AMP1 4 denotes the 1^st^ AMP domain of a total of 4 AMPs in the NRPS. JGI protein IDs are given for *S. turcica*, *C. sativus*, and *C. heterostrophus* strain C5. All other AMPs indicated by NODE number and Augustus gene call number (e.g., Cvict NODE 1785 g7786 t1 1 is a *C. victoriae* AMP on NODE 1785 in the Velvet assembly carrying Augustus called gene 7786; t1 1 indicates the order in which the AMP domain was predicted by HMMER). *C. heterostrophus* strain C4 AMP domains are indicated by scaffold and gene call. Bootstrap values 80% and above are indicated on the branches.

At the genus level, seven of the 14 NRPS proteins (and thus genes) in *C. heterostrophus* reference strain C5 were conserved across all species (Table 7, Figure 3, for full phylogenetic trees see Figure S5A, S5B). When conservation of NRPSs was considered across all *Cochliobolus* spp. and the related maize pathogen *S. turcica*, six of the 14 NRPS proteins in *C. heterostrophus* reference strain C5 were completely conserved across all species (NPS2, 4, 6, 19, and 12, and 12-like, Table 7). The phylogenetic profile (Figure 4) of the highly conserved NPS2 protein is an example of complete conservation. Note that all four NPS2 AMP domains are found in all species. NPS2 biosynthesizes the hexa-peptide siderophore, ferricrocin, and an evolutionary mechanism by which a four AMP domain NRPS can generate a six component metabolite has been proposed by us [58], [59]. In cross genome evaluation of conservation of NRPSs (see Table S18 in [1]), this was one of only two NRPSs conserved across 18 Dothideomycete genomes.

#### Discontinuously distributed and expanded NRPSs

**Figure 5:**
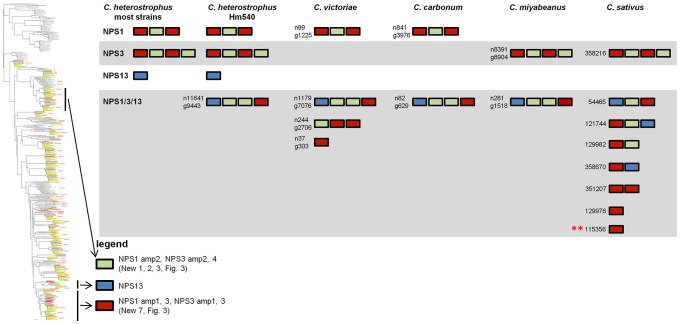
The NPS1/NPS3/NPS13 expansion group of NRPS AMP domains. NPS1 and NPS3 AMP domains are discontinuously distributed and expanded across the *Cochliobolus* isolates sequenced (Figure 6 and [57]). NPS1 and NPS3 AMP 2 and 4 domains (color coded green) group at the top of the phylogenetic tree (thumbnail to the left, full tree Figure S5A), while NPS1, NPS3 and NPS13 AMP domains 1 and 3 (color coded red or blue) group near the bottom of the tree. Each *C. heterostrophus* strain has NPS1 NPS3 and NPS13. The other Cochliobolus species possess either a complete *C. heterostrophus* NPS1 or NPS3 ortholog, but not both and none has NPS13. All species, however, have one or more additional NPS proteins consisting of NPS1/NPS3 or NPS1/NPS3/NPS13-related domains, that are absent from all *C. heterostrophus* genomes except Hm540. The *C. heterostrophus* Hm540 genome includes all four corresponding genes: *NPS1*, *NPS3*, *NPS13*, and the additional *NPS1/NPS3/NPS13* gene. The pattern of AMP domain expansion/loss/recombination is complicated and the additional NRPSs can be mono-, bi-, tri-, and tetra-modular proteins. *C. sativus* has 7 expanded NRPSs whose AMP domains group with NPS1/NPS3/NPS13 proteins, (Figure 6). One of these is *C. sativus* ID 115356 (double asterisk) which maps to a unique region of the genome in pathotype 2 strain ND90Pr (Figure 7), associated with high virulence on barley cultivar Bowman. Gene/AMP nomenclature as described in Figure 4.

**Figure 6:**
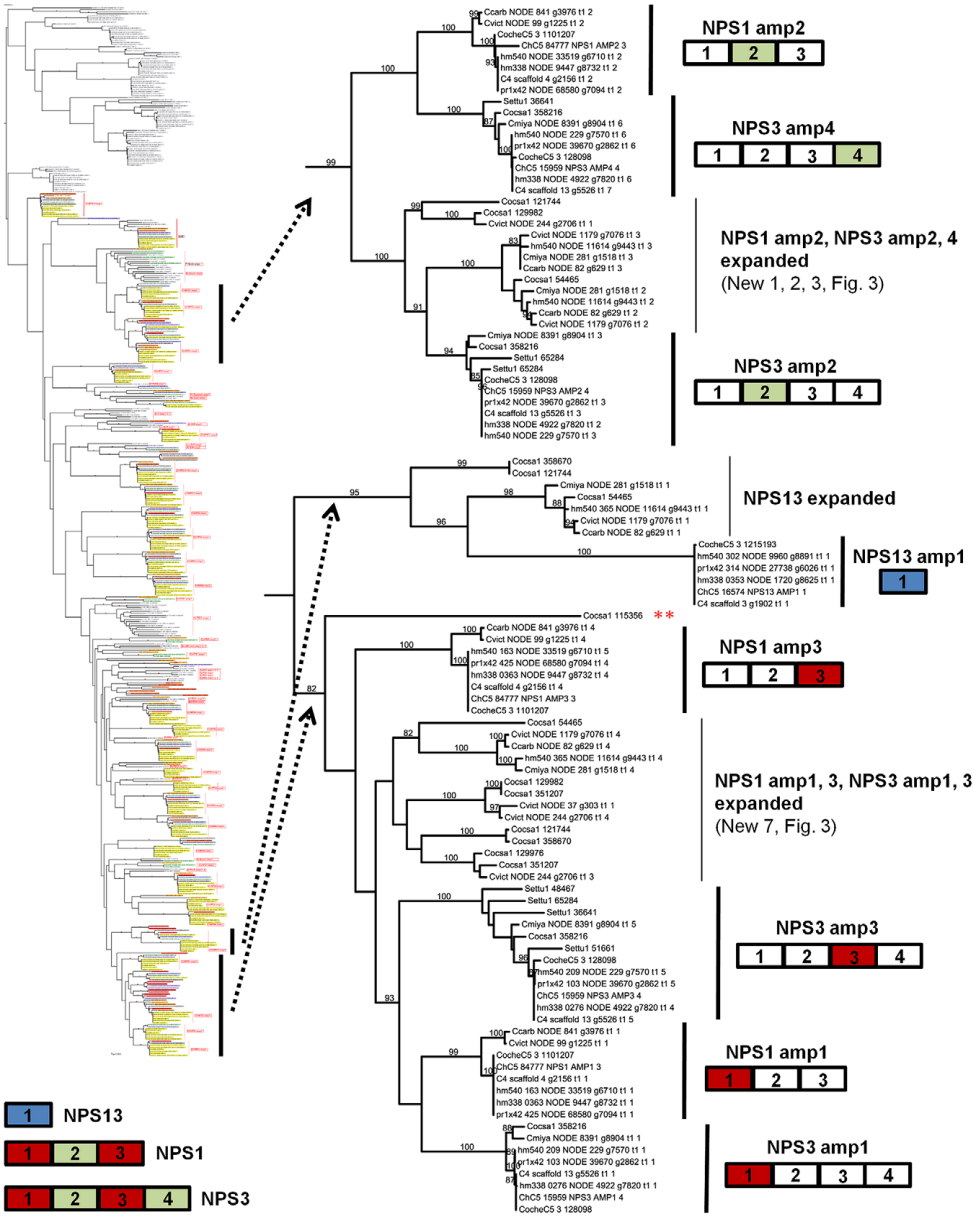
NPS1, NPS3, and NPS13 are examples of NRPS proteins encoded by highly recombinogenic and expanded *NPS* genes. The reference NPS1, NPS3 and NPS13 proteins are cartooned bottom left and AMP domains are color coded as in Figure 5. AMP domains corresponding to these proteins are completely conserved in the five strains of *C. heterostrophus*, but show discontinuous presence in all other *Cochliobolus* species (Figure 5) and *Setosphaeria*. Thumbnail of full AMP tree (Figure S5A) is shown at left. Note some AMP domains from NPS1 and NPS3 group at the top of the tree (AMPs 2 and 4, green), while the rest group at the bottom of the tree (AMPs 1 and 3, red); NPS13 AMP1 (blue) also groups at the bottom of the tree. Branches correspond to individual AMP domains which group together and the particular corresponding AMP domain is colored coded on the right of the diagram. Branches not in the original reference set of *C. heterostrophus* AMPs [57] are labeled as ‘expanded’ (Figure 5). Gene/AMP nomenclature and bootstrap values as described in Figure 4. Double red asterisk indicates *C. sativus* protein ID 115356 discussed in Figure 7).

Of those NRPS proteins for which all *C. heterostrophus* AMP domains are not conserved across all species (Table 7), three, NPS1, NPS3, and NPS13 are of particular note as they are expanded discontinuously, with multiple homologs for some, but not all AMP domains of these proteins in different species (Figure 5, Figure 6). We hypothesize that this group of NRPSs is a spawning ground for AMP domain diversity. On the whole protein level, the complete *C. heterostrophus* NPS1 (trimodular) and NPS3 (tetramodular) domain sets are either present or absent in other species. NPS1 is intact in *C. victoriae* and *C. carbonum*, while NPS3 is intact in *C. miyabeanus* and *C. sativus*, but absent from the other genomes (Figure 5, Table 7). Monomodular *C. heterostrophus* NPS13 is found only in *C. heterostrophus* (Figure 5, Table 7). NPS1, NPS3 and NPS13 protein AMP domains, as noted above, are expanded discontinuously resulting in a suite of novel proteins we call ‘NPS1/NPS3/NPS13 expanded’ (Figure 5). These new proteins may be mono- or multi-modular. *C. victoriae* has three such proteins, *C. carbonum* and *C. miyabeanus* have one each, and *C. sativus* has seven (Table 8, Figure 5). A *C. heterostrophus* NPS13-related module is present in tetramodular form in *C. carbonum*, *C. victoriae*, and *C. miyabeanus*, and in trimodular form in *C. sativus*. Both the monomodular NPS13, and tetramodular NPS13 related protein, are found in the *C. heterostrophus* Hm540 strain.

**Table 8:** Total and unique Cochliobolus NRPS and PKSs.

Species	NRPS					PKS		
	Total	Unique	ID	NPS1/NPS3/NPS13 associated	ID	Total	Unique	ID
*Ch* race T	14	0	NA^a^^, ^ b	3	AAX09983, AAX09985, AY884198	25	2	ABB08104, ABB76806
*Ch* race O	14	0	NA	3(4)	1101207, 128098, 1215193, (n1164, g9443)^c^	23	0	NA
*Cv*	18	5	n3108, g9998; n244, g2706; n37, g303; n1179, g7087; n572, g5163	4	n99, g1225; n244, g2706; n37, g303; n1179, g7076	21	1	n4, g34
*Cc*	20	6	n105 g794; n2409 g6648; n3710, g7564; n559, g3150; n120, g870; n464, g2585	2	n841, g3976; n82, g629	27	2	n2423, g6650; n189, g1086
*Cm*	11	0	NA	2	n281, g1518; n8391, g8904	21	0	NA
*Cs*	25	14	54465, 121744, 129982, 358670, 129976, 351207, 115356, 130053, 140513, 104448, 49884, 103953, 350779, 25865	8	54465, 121744, 129982, 358670, 129976, 351207, 115356, 358216,	18	0	NA

Strains *Ch = C. heterostrophus*, *Cv = C. victoriae*, *Cc = C. carbonum*, *Cm = C. miyabeanus*, *Cs = C. sativus*;

a NA, not applicable;

b for *Ch* C5 and *Cs*, JGI protein IDs are given; for *Ch* C4, GenBank protein IDs are given; all others are Augustus gene call numbers;

c in Hm540 only, See Figures S5A and S6A, Tables S6 and S10.

As shown in Figure 6, *C. heterostrophus* NPS1 AMP2 groups with *C. heterostrophus* NPS3 AMP2 and AMP4 orthologs, with 99% bootstrap support. The NPS1 and NPS3 AMP2 and AMP4 expanded group (New_1, 2, 3, Figure 3) is most closely related to NPS3 AMP2 (100% bootstrap support). *C. heterostrophus* NPS1 AMP1 and AMP3 group with *C. heterostrophus* NPS3 AMP 1 and AMP3 orthologs (NPS1 AMP1, NPS3 AMP 1 and AMP3 group together with 93% bootstrap support). The NPS1 and NPS3 AMP1 and AMP3 expanded group (New_7, Figure 3) is nested within, but without bootstrap support. *S. turcica* has seven total NPS1/3/13 associated AMP domains. Four cluster with NPS3 AMP3, two with NPS3 AMP2, and one with NPS3 AMP4 (Figure 6, Table S6). These expanded *S. turcica* domains belong to four different NRPSs (ID# 51661, 36641, 65284, 48467), none of which corresponds to Cochliobolus NRPSs.

Monomodular NPS13, as noted, is found only in *C. heterostrophus*, but there is an NPS13 expanded group of AMP domains, sister to (95% bootstrap support) *C. heterostrophus* NPS13. *C. sativus* possesses additional NPS13 expanded domains, always co-occurring with NPS1 and NPS3 expanded domains (Figure 5, Figure 6).

#### Species-unique NRPSs

In initiating our survey, we hypothesized that this category of NRPS would be most likely to include candidates associated with virulence functions, given the unique or spotty distribution signatures of HSTs. We define an NRPS as unique when no other *Cochliobolus* species has all of the orthologous AMP domains in identical whole-protein organization. The reference, *C. heterostrophus*, has 14 NRPSs, three of which (NPS5, NPS8 and NPS9) are unique to this species but found in both race O and T (Table 7, Table 8, Table S6). None of these three has an obvious role in virulence [55]. Of the other *Cochliobolus* species, *C. miyabeanus* has the fewest total (11) NRPSs and no unique ones, while *C. sativus* has the most (25), 14 of which are unique (Table 8) and include seven belonging to the NPS1/NPS3/NPS13 expanded group (and an eighth unique NRPS, ID# 358216) (Table 8, Figure 5). When the AMP domains from the 25 NRPSs identified in *C. sativus* isolate ND90Pr (pathotype 2) were used as blast queries to identify orthologs in *C. sativus* isolate ND93-1 (pathotype 0), five (ID# 130053, 140513, 104448, 115356, and 350779) were not present in the latter and thus are unique to ND90Pr.

*C. carbonum* and *C. victoriae* have 20 and 18 total NRPSs, respectively. Six are unique to *C. carbonum* (Table 8). None of these is in the NPS1/NPS3/NPS13 expanded group. One of the *C. carbonum* unique NRPSs is HTS1, responsible for HC-toxin biosynthesis. It has long been recognized that HTS1 is only found in race 1 of this species and not in any other Cochliobolus species [60] and our genome survey confirms this. Our NRPS survey, however, identified an ortholog in *S. turcica* (see section below). Four other novel *C. carbonum* NRPS AMP domains group in the 11 AMP domain *Tolypocladium inflatum* SimA clade for biosynthesis of cyclosporin, suggesting *C. carbonum* as a possible source of a cyclosporin-type molecule (Table 8, Figure S5). *C. victoriae* has five unique NRPSs, including two from the NPS1/NPS3/NPS13 expanded group (Figure 5, Table 8). One of the *C. victoriae* unique NRPSs is on n1179 (g7087) and is an ortholog of the gene for gliotoxin. Another *C. victoriae* unique AMP domain (on node 572, Figure S5A) is incomplete but has a match to a bimodular *S. turcica* NRPS (ID #97841). The fifth unique AMP, on node 3108 (Figure S5A), groups with the NRPS for aminoadipate reductase (lysine biosynthesis), but without bootstrap support.

For *S. turcica*, eight (ID# 36641, 48467, 51661, 65284, 99043, 155102, and 54477, 97841, 99181 Table S6) NRPSs were unique, not occurring in any *Cochliobolus* genome. One of these, 99181, clusters with varying support with PesA, a *Metarhizium anisopliae* NRPS producing an unknown product. Four of *S. turcica's* unique NRPSs (ID# 36641, 48467, 51661, 65284) contained NPS1/NPS3/NPS13 expansion AMP domains as noted above (Table S6).

#### NRPSs showcasing the value of the phylogenomic approach in pinpointing candidate virulence determinants

**Figure 7:**
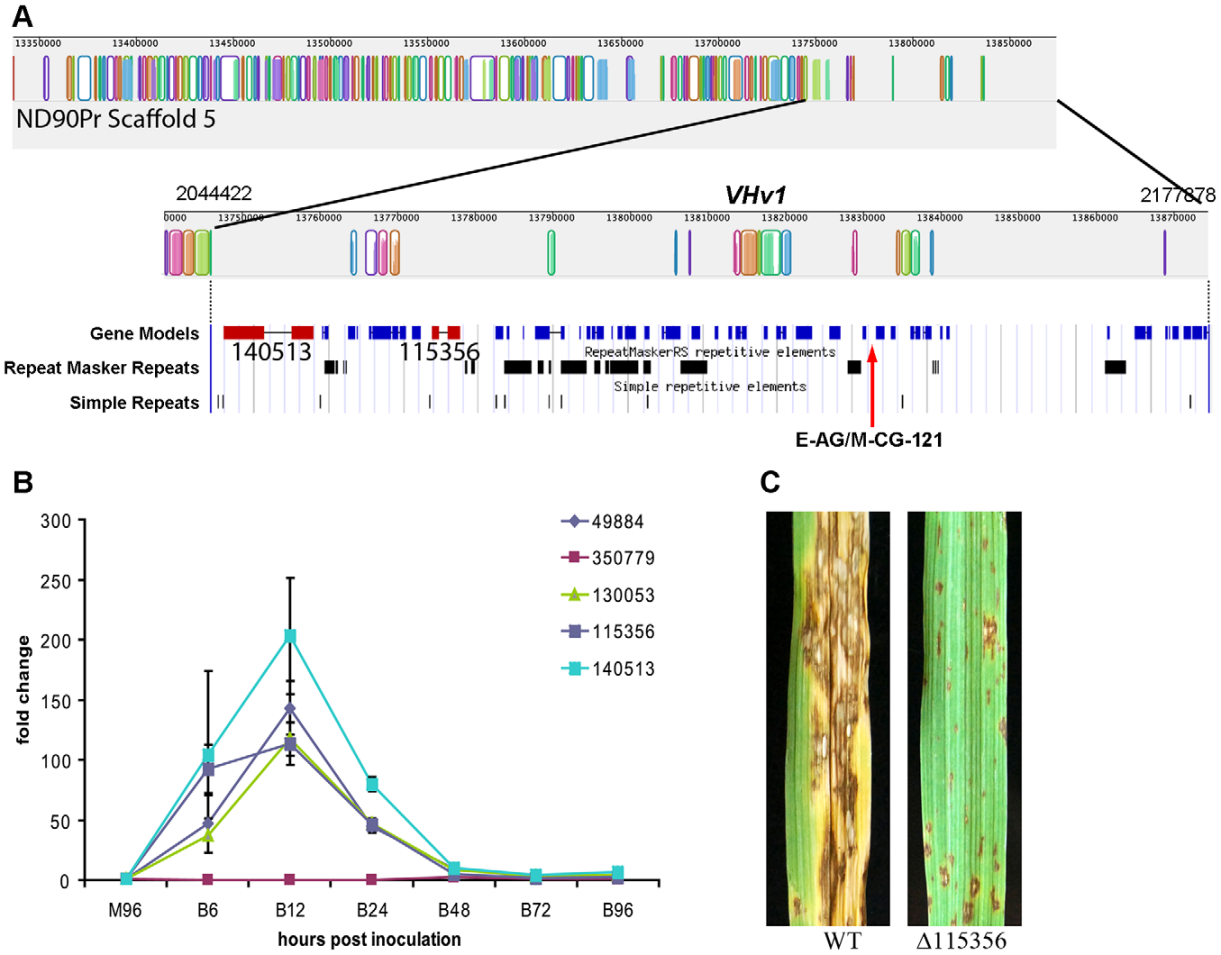
Genomic organization of the scaffold associated with the *VHv1* locus conferring high virulence of pathotype 2 isolate ND90Pr to barley cv. Bowman compared to the corresponding region in pathotype 0, isolate ND93-1. A. Mauve alignment [44] of ND93-1 scaffolds to ND90Pr. Colored blocks [Locally Collinear Blocks (LCB)] indicate matches between the two genomes, and vertical block shading corresponds with % similarity. Note very few colored blocks at right end of top row. The ∼133 kb *VHv1* locus, which maps to distal end of scaffold 5 (2.18 Mb), is unique to isolate ND90Pr, as indicated by absence of colored blocks at right end of the top row and the second row which is the same region at higher resolution. Below this Mauve alignment segment of the *VHv1* region on scaffold 5 (from position 2,044,422 to 2,177,878) is the JGI browser view of the same region, displaying gene models and repeats. There are forty-three predicted genes (blue) in this region, only a fraction of which have KOG or GO descriptions (Table S7). Two NRPSs (ID # 115356 and 140513, shown in red) map to this region and are unique to the ND90Pr isolate and also not found in any of the genomes examined in this manuscript (Figure 3, Figure 5, Table 8). E-AG/M-CG-121 is one of two AFLP markers (the other is E-AG/M-CA-207 on scaffold 40) are linked to the virulence locus, *VHv1* (Figure S3). B. Quantitative real-time PCR analysis of five *NPS* genes (protein ID 49884, 350779, 130053, 115356, 140513) during infection of barley cv. Bowman. Gene expression was normalized based on the expression of the β-Actin gene, and the values are the relative expression levels in comparison with M96, a mixture of mycelia harvested, at 96 hours after culture set up, from different media including PDA, MM, V8PDA, and water agar. B6, B12, B24, B48, B72, and B96 are samples collected at 6, 12, 24, 48, 72, and 96 hours after inoculation. Primers are shown in Table S9. The error bars indicate the minimum and maximum values of relative expression of the gene. C. Inoculation of barley cv. Bowman with wild type (ND90Pr) and a mutant lacking the gene corresponding to protein ID 115356. Images taken 7 days after inoculation. Virulence on Bowman is significantly reduced compared to that of plants inoculated with the wild-type strain (Figure S7).

Case Study 1: NRPSs unique to C. sativus pathotype 2, isolate ND90Pr determine virulence on barley cultivar Bowman. Previous genetic studies have indicated that a single locus (*VHv1*) in *C. sativus* pathotype 2 isolate ND90Pr controls high virulence on barley cv. Bowman [30]. Two AFLP markers E-AG/M-CG-121 and E-AG/M-CA-207 that co-segregate with the *VHv1* locus in ND90Pr [30] mapped to scaffold 5 (Figure 7A) and 40 (Figure S3), respectively, in the genome assembly. The *VHv1* region (distal end of scaffold 5) carrying the E-AG/M-CG-121 marker includes 43 predicted genes (Table S7), plus many repetitive elements (Figure 7A, Table S8). None of the 43 genes was found in the *C. sativus* pathotype 0 isolate ND93-1 genome. Two of the genes in the region encode NRPS (ID# 115356, 140513), mentioned above as unique to isolate ND09Pr. 115356 is on a branch by itself in the NPS1/NPS3/NPS13 expansion group on the NRPS AMP tree (Figure 6, Figure 5, double asterisks, Table 8) and is not found in any of the other *Cochliobolus* species, in *S. turcica*, or in Genbank. NRPS ID# 140513, also unique to the *VHv1* region (Figure 7), maps to a *C. sativus-*specific clade we call ‘New_8’, consisting of seven AMP domains (Figure 3, asterisk) corresponding to three additional *C. sativus-* unique NRPSs. One of these is ID# 130053 with three AMPs on scaffold 25. Based on proximity in the genome assembly, we believe a second unique AMP, in protein ID# 49884, also on scaffold 25, is actually a fourth AMP domain of 130053. The remaining NRPS grouping in the New_8 clade is ID# 104448.

Differences in *NPS* gene content and pathogenicity phenotype of closely related *C. sativus* strains allowed us to identify candidates for functional analyses. We hypothesized that, since the *VHv1* region is unique to isolate ND90Pr and contains two NRPSs (ID#140513, 115356) unique to *C. sativus*, one or both of these might be responsible for high virulence on barley. We conducted real time PCR (Table S9) on infected barley leaves and demonstrated that expression levels of the genes corresponding to 140513 and 115356 in the *VHv1* region, and also of the genes corresponding to unique proteins 130053 and 49884 described above were up-regulated 12 hours post inoculation (Figure 7B), while the gene corresponding to protein ID# 350779, which maps in the Gliotoxin clade (Figure S5A, Table S9), was not. Deletion of the gene corresponding to protein 115356 indicates that, indeed, it is involved in the high virulence of ND90Pr on cv. Bowman, as the mutant is significantly reduced in virulence to the host, compared to the wild-type strain (Figure 7C, Figure S7). Thus, our comparative approach to analyzing secondary metabolite core proteins (NRPSs) led to the identification of a unique genomic region in *C. sativus* pathotype 2 isolate ND90Pr associated with high virulence on barley cv. Bowman that carries NRPSs which, when functionally manipulated, impacted virulence.

**Figure 8:**
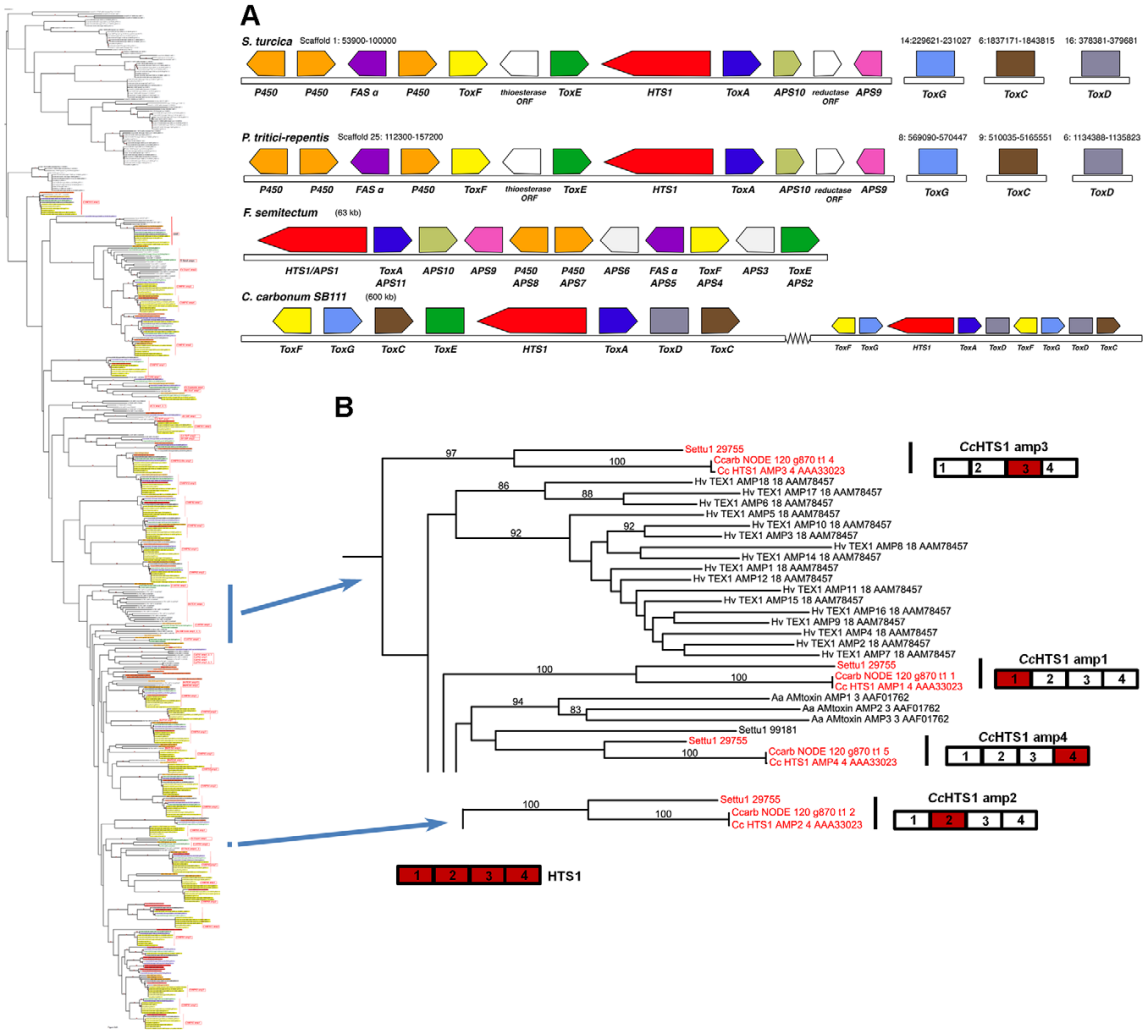
*S. turcica* has an ortholog of the *C. carbonum* NRPS HTS1 responsible for HC-toxin biosynthesis. A. Gene annotation and comparisons of the *S. turcica*, *P. tritici-repentis*, and *F. semitectum* regions carrying orthologs of the HC-toxin locus genes in *C. carbonum* strain SB111. Protein designations (color coded) correspond to *C. carbonum* and *F. semitectum* (APS) nomenclature. HTS1 is an NRPS, ToxA, E, F correspond to efflux pump, DNA-binding, and branched chain amino acid transaminase, proteins, respectively, and FAS α is a fatty acid synthase alpha subunit. Tox C (FAS beta subunit), ToxD (dehydrogenase), and ToxG (alanine racemase) in the cluster in *C. carbonum*, are not clustered in the other species but map to different scaffolds in the *S. turcica* and *P. tritici-repentis* assemblies. In *C. carbonum*, all of the known genes required for HC-toxin production are multicopy, in two linked, but separated clusters in a 600 kb region in isolate SB111; the genes are absent from toxin non-producing *C. carbonum* isolates that have been examined [122]. B. Portions of the full phylogenetic tree (Figure S5A) showing placement of the HTS1 AMPs, extracted from tree to the left. HTS1 has four AMP domains cartooned bottom left. Each *C. carbonum* AMP domain (red), groups, with high bootstrap support, with *S. turcica* protein 29755, a four AMP domain NRPS, (red), except for AMP4. In each of these matches, *C. carbonum* is represented twice, once by the SB111 AMP domain of the deposited sequence #AAA33023, and once as extracted by Augustus from our Illumina Velvet assembly of strain 26-R-13. Note all HTS1 AMP domains group separately one from another and AMP2 is distant from the others.

Case Study 2: Among Cochliobolus species, the NRPS HTS1, which biosynthesizes the tetrapeptide HST HC-toxin, is unique to *C. carbonum* race 1 and has been demonstrated previously to be required for pathogenicity to *hmhm* maize [4], [18], [19]. HTS1, however, is present in *S. turcica* and other fungi. Given the thorough documentation of HTS1 as a pathogenicity determinant, functional analyses were not necessary to cement the connection between its unique signature within the genus and its role as a virulence determinant. With the wider genomic resources reported here, we found orthologs of all four HTS1 AMP domains in *S. turcica* (ID# 29755) (Figure 8A). Manning et al., [61] also report orthologs in *P. tritici repentis* (ID# 12015) and Wight and Walton have found an ortholog in *Alternaria jesenkae* and demonstrated, furthermore, that the isolate makes HC-toxin [62]. In addition, there are HTS1 orthologs (APS1, Acc#: ACZ66258) in the Sordariomycete, *Fusarium incarnatum/semitectum*, that biosynthesize a different metabolite, apicidin [63].

In *C. carbonum* race 1 strain, SB111, the original strain in which the *HTS1* locus was described, the structural organization of the cluster of genes encoding enzymes for HC-toxin production is complex and includes two copies of most genes, in two clusters residing in an ∼600 kb region. The organizations of the *S. turcica* and *P. tritici-repentis* clusters are similar to each other, but different from that described for *C. carbonum* (Figure 8). Firstly, there is no evidence that the *S. turcica* and *P. tritici-repentis* genes are duplicated. Secondly, only orthologs of *C. carbonum* HTS1, ToxA, ToxE, and ToxF (*C. carbonum* HTS1 cluster nomenclature) proteins are clustered in *S. turcica* and *P. tritici-repentis*; orthologs of *C. carbonum* ToxC, ToxD, and ToxG proteins are found in both genomes, but on separate scaffolds in each genome (Figure 8). HTS1, ToxA, ToxE, and ToxF orthologs are present in the *F. semitectum* APS1 cluster, however there is no ToxC, ToxD, and ToxG in the sequenced cluster and, as genome sequence is not available for this species, we were unable to search for these genes.

Thus the phylogenetic approach, when conducted with the suite of *Cochliobolus* genomes, pinpointed the NRPS, HTS1, as unique to *C. carbonum* race 1 and functional analyses done previously, prove that the metabolite is an HST required for pathogenicity. The inclusion of a genome from a genus sister to *Cochliobolus*, however, identified an ortholog in *S. turcica*. This, combined with reports of HTS1 orthologs in other phylogenetically scattered groups, indicates a complex genetic history.

**Figure 9:**
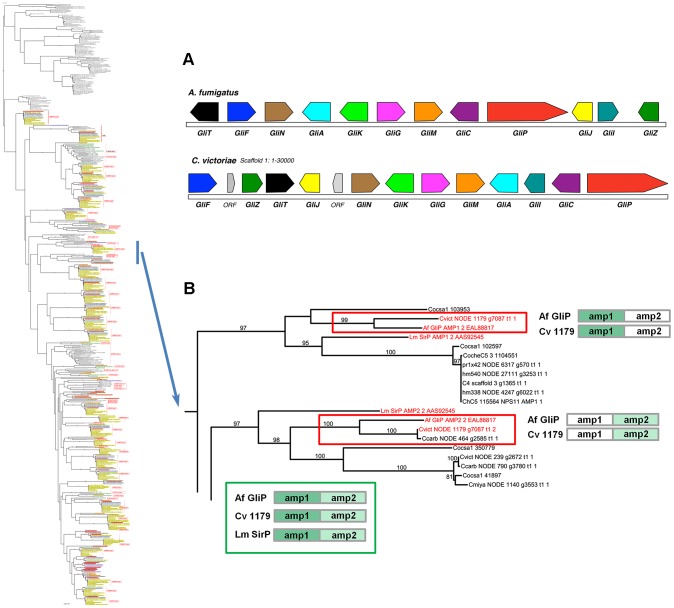
*C. victoriae* has an ortholog of *A. fumigatus* GliP responsible for gliotoxin production. A. Gene annotation and comparisons of the *C. victoriae* and *A. fumigatus* regions carrying orthologs of the Gliotoxin biosynthetic proteins. Protein designations (color coded) correspond to *A. fumigatus* nomenclature [64]. In *A. fumigatus*, GliP is an NRPS, GliT, F, N, A, G, M, C, J, I and Z correspond to oxidase, cytochrome P450, methyl transferase, transporter, glutathione S-transferase, *O*-methyltransferase, cytochrome P450, dipeptidase, aminotransferase and Zn finger proteins, respectively. GliK is of unknown function. In *C. victoriae*, ‘ORF’ = unknown function. B. The bimodular (2 AMP domains) *C. victoriae* NODE 1179 NRPS (Augustus gene call g7087) is an example of the phenomenon of spotty conservation of NRPS AMP domains across fungi. *L. maculans* also has an ortholog (SirP, producing sirodesmin), however, this is not as closely related as the *C. victoriae* ortholog. Of the two AMP domains that comprise the *C. victoriae* GliP ortholog, AMP2 is present in *C. carbonum* (NODE 464 g2585) and AMP1 is found in *C. sativus* (ID 103953). Neither possesses both, although additional, related sister domains are found in other *Cochliobolus* species. Cartoon (bottom) shows the *A. fumigatus*, *C. victoriae* and *L. maculans* NRPS orthologs color coded as to AMP domain. Branches carrying GliP orthologs extracted from full phylogenetic tree (Figure S5A) to left. Gene/AMP nomenclature and bootstrap values as described in Figure 4.

Case Study 3: *C. victoriae* has a NRPS that groups with high support with the *A. fumigatus* NRPS for Gliotoxin biosynthesis. In addition to the NRPSs extracted from our sequenced genomes, our phylogenetic trees included NRPSs producing known products, such as the two AMP domain *A. fumigatus* NRPS, GliP, for gliotoxin biosynthesis and the *Leptosphaeria maculans* NRPS, SirP, for sirodesmin production, both epipolythiodioxopiperazine (ETP) toxins. Two *C. victoriae* AMP domains on node 1179 clustered with 99–100% bootstrap support with *A. fumigatus* GliP AMP1 and AMP2 (Figure 9, Table 8). Both were evolutionarily closer than the corresponding SirP AMP domains. Thus, our objective to determine if any of the unknown NRPSs grouped with NRPSs with characterized products yielded a *C. victoriae* candidate for production of gliotoxin or a related metabolite. Furthermore, examination of the neighborhood surrounding the *C. victoriae* bimodular NRPS, indicates that all of the genes in the *A. fumigatus* gene cluster [64] are present in a cluster in *C. victoriae* (Figure 9A).

#### Summary

To thoroughly understand the evolutionary history of multimodular NRPSs, AMP domains were analyzed as individuals using a combination of amino acid alignments and phylogenetic tree building. Results show that, within a species, NRPSs are highly conserved, but conservation dissipates as comparisons are made across the genus. Thus, diversity of these genes, their encoded proteins and corresponding metabolite potential, is truly enormous. Strain-unique NRPSs are primary suspects for producing small molecules conferring high virulence or host specificity. A robust example of this are the species- and strain-unique *C. sativus* ND90Pr NRPS proteins 115356 and 140153 which map to the unique *VHv1* high virulence conferring region, for which gene deletion confirms a role in cultivar specific virulence. The phylogenetic structure of NPS1 and NPS3 enzymes suggest that corresponding genes undergo rapid duplication and expansion and could act as a cauldron for the formation of new *NPS* genes.

### Secondary metabolism: Polyketide synthases

Polyketide synthases (PKSs), like NRPSs, are large multidomain enzymes that produce small molecules (polyketides) with functions that include HSTs. The suites of PKS encoding genes (*PKS*) in the *C. heterostrophus* C4 and C5 genomes were identified and annotated previously [65]. To address degree of conservation and evolutionary relationships of PKSs in our subject species in order to make inferences about function, we used the PFAM ketosynthase domain (KS) HMM as a query to search for orthologs in the additional strains of *C. heterostrophus* and other species, and the related maize pathogen, *S. turcica*.

#### Conservation of known reference strain *C. heterostrophus* C5 polyketide synthases

**Figure 10:**
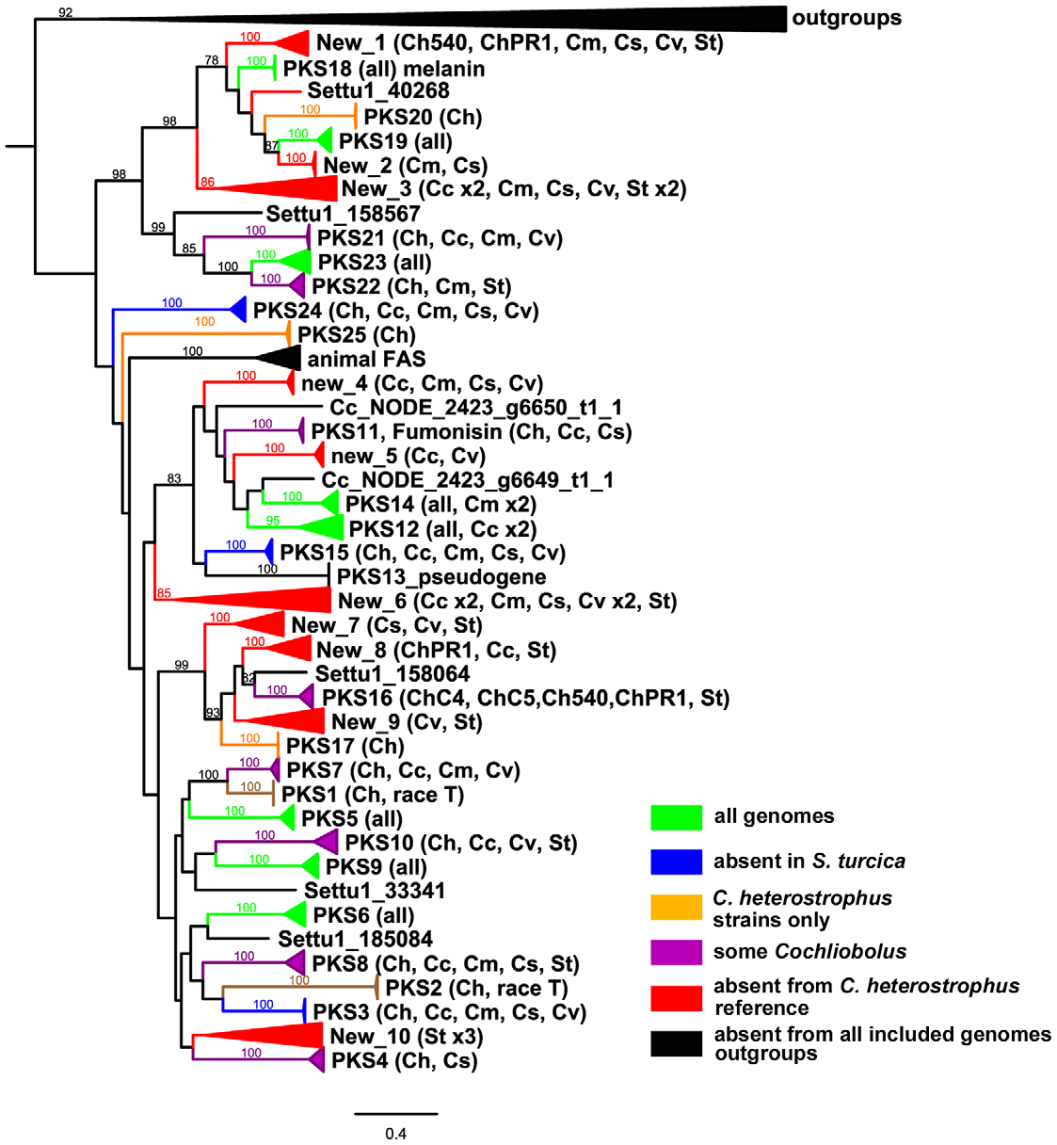
Cartoon of cross-species phylogenomic analyses of individual ketosynthase domains from PKS proteins. The ketosynthase (KS) domains were extracted from all five *C. heterostrophus* and from the *C. victoriae*, *C. carbonum*, *C. miyabeanus*, *C. sativus* and *S. turcica* genomes. See Figure 3 for species designations, color codes and format. KS domains colored black and therefore absent in analyzed genomes include outgroups and KS domains in animal fatty acid synthases (FAS). KS domains not grouping with the previously annotated *C. heterostrophus* set are labeled as ‘New _1 through _10’. Gene/KS nomenclature and bootstrap values as described in Figure 3 and Figure 4 for AMP domains.

Most *C. heterostrophus* PKSs are conserved across all *C. heterostrophus* strains, although PKS16 is absent from the genome of strain Hm338 and PKS25 is absent from strain Hm540 (Table 9, Figure 10, for the full phylogenetic trees see Figure S6, and Table S10 for master inventories). PKS13 is a pseudogene found only in strain C5 (and C4). As with the NRPSs, conservation of PKSs across the *Cochliobolus* genus is not as high as within *C. heterostrophus* species and is even less when the related genus, *S. turcica* is considered. Seven out of the 23 PKSs in reference strain C5 are conserved in all *Cochliobolus* species and *S. turcica* (Table 9, Figure 10); the only known product of these is melanin (produced by *C. heterostrophus* PKS18). Otherwise, the products of conserved PKSs are unknown. Three PKSs are present in all *Cochliobolus* genomes, but not *S. turcica* (Table 9). Two PKSs are unique to *C. heterostrophus*, while nine are present discontinuously throughout the species examined. Blast searches using the predicted protein sequences of *C. sativus* isolate ND90Pr PKSs as queries against the genome sequences of the isolate ND93-1 (a pathotype 0 isolate) indicated that all PKSs predicted in ND90Pr were found in ND93-1, except one (ID# 184740).

**Table 9:** Conservation of *C. heterostrophus* strain C5 polyketide synthases in other strains and species.

*Ch*Gene	Genbank C4 protein ID	JGI C5.V3 Protein ID	In all *C. heterostrophus* strains?	In all *Cochliobolus* spp?	In *S. turcica*?
*PKS1*	ABB08104		Race T	−	−
*PKS2*	ABB76806		Race T	−	−
*PKS3*	AAR90258	1098212	+	+	−
*PKS4*	AAR90259	96868	+	*Cs*	−
*PKS5*	AAR90260	1103337	+	+	+
*PKS6*	AAR90261	1168707, 1229323	+	*Cv*, *Cc*, *Cm*	+
*PKS7*	AAR90262	1118456	+	*Cv*, *Cc*, *Cm*	−
*PKS8*	AAR90263	28817	+	*Cc*, *Cm*, *Cs*	+
*PKS9*	AAR90264	1104189	+	+	+
*PKS10*	AAR90265	96669	+	*Cv*, *Cc*	+
*PKS11*	AAR90266	1112706	+	*Cc*, *Cs*	−
*PKS12*	AAR90267	1216295	+	+, 2x *Cc*	+
*PKS13*	AY495653	75987	−		
*PKS14*	AAR90268	67271	+	+, 2x *Cm*	+
*PKS15*	AAR90269	108173	+	+	−
*PKS16*	AAR90270	33896	not in Hm338	−	+x2
*PKS17*	AAR90271	1105179	+	−	−
*PKS18*	AAR90272	34478	+	+	+
*PKS19*	AAR90273	81477	+	+	+
*PKS20*	AAR90274	1108855	+	−	−
*PKS21*	AAR90275	1029307	+	*Cv*, *Cc*, *Cm*, *Cs*	−
*PKS22*	AAR90276	105287	+	*Cm*	+
*PKS23*	AAR90277	77059	+	+	+
*PKS24*	AAR90278	1209664	+	+	−
*PKS25*	AAR90279	1034546	not in Hm540	−	−

+ = present, − = absent; 2x = two copies;

Strains *Ch* = *C. heterostrophus*, C4, Hm338, Hm540, PR1x412 are *C. heterostrophus* strains,

*Cs = C. sativus*, *Cv = C. victoriae*, *Cc = C. carbonum*, *Cm = C. miyabeanus*, *St = S. turcica*,

for *Ch* C4 Genbank protein IDs are given, for *Ch* C5 JGI protein IDs are given.

See Figure S6A and Table S10.

#### Species-unique PKSs

As for the NRPSs, we anticipated that species-unique PKSs (PKSs possessing KS domains lacking orthologous KS domains with bootstrap support in other species) would be the likeliest candidates for virulence functions, given the well-documented roles of HSTs in virulence and their corresponding unique or spotty distribution patterns in related strains. *C. heterostrophus* race O has 23 PKSs and no unique ones, while *C. heterostrophus* race T has 25 PKSs, two of which are unique. *C. victoriae*, *C. miyabeanus*, and *C. sativus* have 21, 21, and 18 PKSs, respectively, and no unique ones; *C. carbonum* has 27 PKSs including two unique ones, and *S. turcica* has 27 PKSs, including 13 unique ones (Table S10).

#### Discontinuously distributed and expanded PKSs

There were ten clusters (designated ‘New’ 1–10) of *PKS* genes that did not have an ortholog in *C. heterostrophus* (Figure 10, Table S10). Some of these clusters, such as ‘New_2 or New_5’, had representatives of only a few species. Others such as ‘New_3 or New_6’ contained representatives of all species except *C. heterostrophus*. Two clusters (New_1 and New_8) were not sister to a group with a *C. heterostrophus* reference PKS, but contained an ortholog from a *C. heterostrophus* field strain (Figure 10).

The only expanded group of PKSs from the *C. heterostrophus* set was PKS14, which had two orthologs in *C. miyabeanus* (Table 9, Figure 10). Otherwise, *C. heterostrophus* PKSs were either conserved as single copies, discontinuously present in single copy, or *C. heterostrophus* unique. Expansion did not seem to occur centered around a certain set of ‘birthing reservoir’ genes, as for NPS1 and NPS3 AMP domains (Figure 6).

#### PKSs showcasing the value of the phylogenomic approach in pinpointing candidate virulence determinants

**Figure 11:**
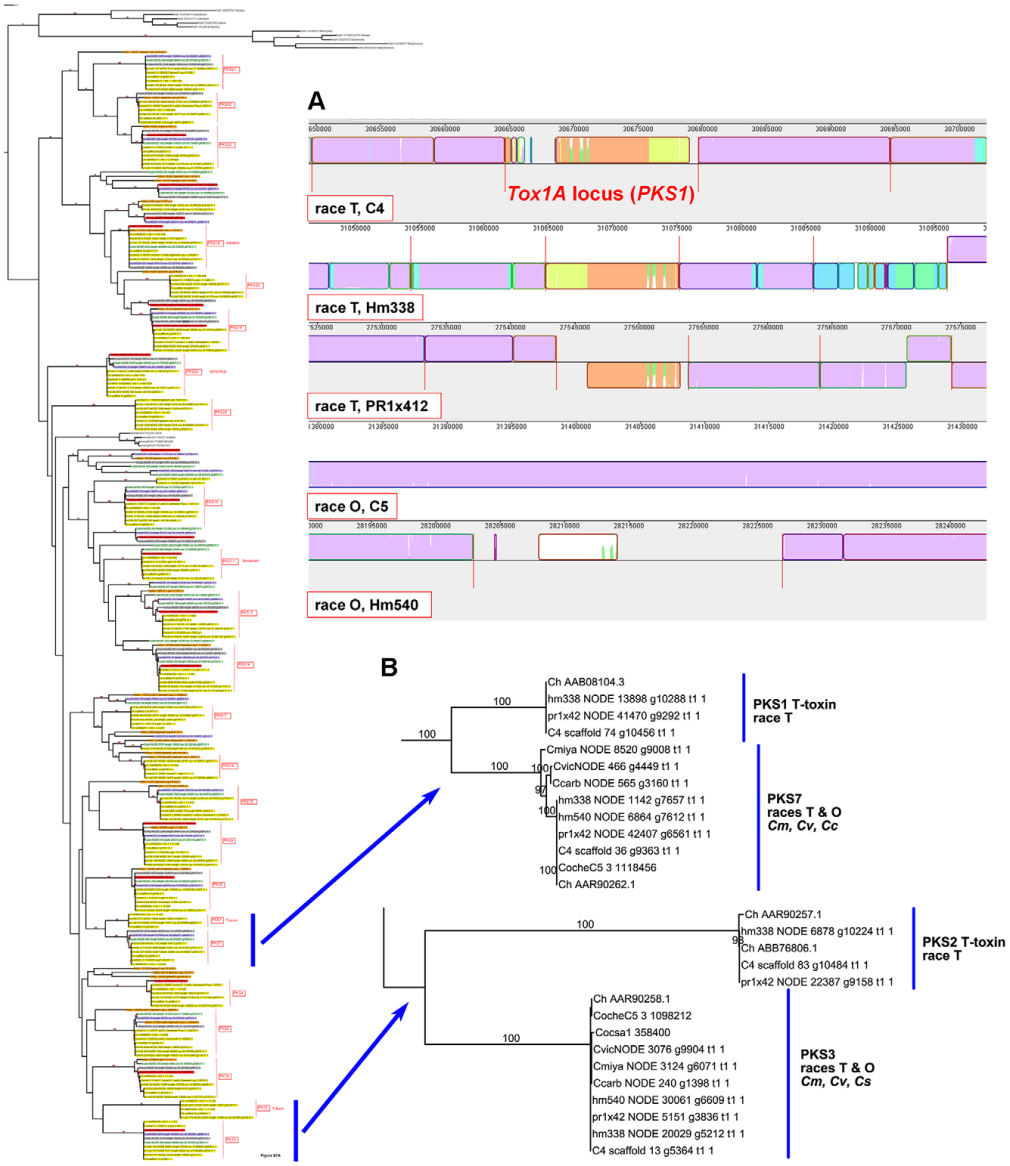
The two PKSs responsible for T-toxin production by race T of *C. heterostrophus* are unique to race T. A. Mauve alignment [44] of the sequences of three race T (C4, Hm338, PR1x412) and two race O (C5, Hm540) strains of *C. heterostrophus*. Sequences in common across all five genomes are colored ‘mauve’. The three orange blocks correspond to regions found only in race T genomes and carry the *PKS1* gene essential for T-toxin biosynthesis and high virulence on cytoplasmic male sterile corn. B. PKS1 and PKS2 are found in race T strains only and are unrelated by phylogeny. In *C. heterostrophus*, PKS1 is most closely related to PKS7 (high bootstrap support), which is found in both race T and race O strains, as well as *C. victoriae*, *C. carbonum*, and *C. miyabeanus*, but not in *C. sativus* or *S. turcica*. PKS2 groups in a different location with PKS3, but without bootstrap support, and is found in all strains examined except *S. turcica*. Full phylogenetic tree is to left (Figure S6A). The reference set of *C. heterostrophus* strain C4 PKSs are shown by their Genbank numbers [e.g., Ch AAB08104, which is PKS1 (Table 9)] and their Augustus gene call (C4 scaffold 74 g10456) and thus are in the tree in duplicate. C5 proteins are indicated as JGI protein ID (e.g., CocheC5 1118456). For more information on evolutionary relationships of PKS1 and PKS2 see [45].

Case Study 1: All race T strains of *C. heterostrophus* have two *PKS*s not found in race O strains or any other known species. *PKS1* and *PKS2*, genes required for biosynthesis of T-toxin, are present in all *C. heterostrophus* race T strains but absent from all *C. heterostrophus* race O strains and all *Cochliobolus* species examined to date (Figure 10, Figure 11A, Table 9). In previous work, we demonstrated that strains deleted for either of these two *PKS* genes fail to make T-toxin and are much reduced in virulence on T cytoplasm corn [45], [48], [66], [67]. This race-specific PKS example mirrors the aforementioned NRPS examples, i.e., the *C. sativus* ND90Pr region carrying the NRPSs 115356 and 140513 (Figure 7) and the *C. carbonum* region encoding HTS1 (Figure 8).

Case Study 2: A *S. turcica*-specific PKSs is up-regulated *in planta*. Three *S. turcica* PKSs grouped together in a *S. turcica*- specific clade, labeled ‘New_10’ (Figure 10, Table S10). Using real time PCR, we examined *in planta* expression of one of the *S. turcica* unique genes (ID 161586) and found that expression was increased 560-fold at three days post inoculation (Figure S8), then dipped and rose again to the same level at seven days post inoculation. Although we haven't yet deleted this gene, based on other test cases, it is tempting to couple the *in planta* expression pattern with a possible role in virulence.

#### Summary

Like NRPS proteins, the PKSs examined were either highly conserved, partially conserved, or strain unique. Some orthologs had duplicated members for some species, but this expansion did not orbit a particular set of genes such as *NPS1* and *NPS3*. PKSs identified as strain or species-unique include characterized, as well as unknown, candidate virulence factors. The race T unique *C. heterostrophus PKS* genes *PKS1* and *PKS2* are examples of characterized unique, highly specific virulence factors. Further characterization of strain-unique *PKSs*, such as *S. turcica* ID 161586, which is highly expressed *in planta* (Figure S8) could reveal novel virulence factors.

### Location of *C. heterostrophus NPS* and *PKS* genes

Several publications demonstrate that species/strain unique sequences tend to reside in variable regions of the genome such as in subtelomeric locations [68], [69] and dispensable chromosomes [70]. All *C. heterostrophus* reference strain C5 *NPS* and *PKS* genes were mapped to the assembled linkage groups (Figure 2). For *NPS*s, 13 of the 14 total could be mapped to one of the 16 linkage groups/chromosomes, and 6 of the 13 were <200 kb from the end of the linkage group. Two (*NPS 5*, *9*) of the six are unique to *C. heterostrophus* and two (*NPS1*, *11*) have limited distribution in *Cochliobolus* spp.. For *PKSs*, 22 of the 25 total could be mapped to one of the 16 linkage groups, 9 of the 22 were <200 kb from the end of the corresponding linkage group and one (*PKS25*) mapped to the B chromosome. Five (*PKS 13*, *16*, *17*, *20*, *25*) of the ten are unique to *C. heterostrophus* and one more (*PKS11*) has limited distribution in *Cochliobolus* spp.. In sum, approximately half of the *NPS*s and *PKS*s map to scaffold ends, in some cases with mapped telomeres (Figure 1). As chromosome ends are notoriously variable, this placement could indicate a mechanism for patchy phylogenetic distribution of these genes.

Note that the two *PKSs* involved in T-toxin production by race T are absent in race O strain C5, but map (genetically) to the breakpoints of race T chromosomes 12;6 and 6;12 which are reciprocally translocated with respect to chromosomes 6 and 12 in race O C5 [9]. Note also that *PKS3*, which has a phylogenetic relationship, but without bootstrap support, to *PKS2* (Figure 10, Figure 11B), maps internally to race O chromosome 6 (Figure 2), and that *PKS7*, which is the closest (with bootstrap support) *C. heterostrophus PKS* to *PKS1* (Figure 11B), maps to the end of unplaced scaffold 20 (Figure 2).

### Small secreted proteins (SSPs)

To identify candidate effector proteins, we searched the gene catalog of each species for proteins that were cysteine rich (≥2% cysteine), small (<200 amino acids), predicted to be secreted (using Phobius [71]), and without transmembrane domains. Between 141 and 289 SSPs per genome (Table 10) were identified with *C. sativus* ND90Pr containing the most and *C. heterostrophus* Hm338 the fewest. We next conducted an all versus all blast analysis to determine if SSPs were strain or species-unique, using an 80% blast cutoff. Very few *C. heterostrophus* SSPs were unique to any particular strain as most could be found in at least one other *C. heterostrophus* field or lab strain. Using this approach, we identified between one and 21 unique SSPs (Table 10, master inventory Table S11). We found more strain-unique SSPs in the other *Cochliobolus* genomes, as our analysis included five *C. heterostrophus* strains. *S. turcica* and *C. sativus* had the most isolate-unique SSPs, containing 191 and 167 candidates, respectively. As these are the two strains thought to act as hemibiotrophs, it is interesting that they contain more SSPs, and more unique SSPs, than the necrotrophic isolates, although this is only a correlation at this point.

**Table 10:** Comparative small secreted protein candidate effector inventories.

Strain	Total SSPs	Strain unique
total	1753^a^	506
*Ch* C5	180^b^	14
*Ch* C4	171	21
*Ch* 338	141	1
*Ch* Hm540	151	4
*Ch* PR1x412	151	8
*Cc* 26R13	153	24
*Cv* FI3	160	25
*Cm* WK1C	143	51
*Cs* ND90Pr	289	167
*St* 28A	214	191

a Total SSPs for all strains examined.

b Total for each strain (e.g., *Ch* C5).

The *C. heterostrophus* C5 assembly has 180 predicted secreted proteins matching the criteria. We examined each of these in the JGI browser with respect to EST support, SNPs, and predicted functional domains. Seventy-two of these calls had absolutely no EST support, while 24 calls had incomplete EST support (ESTs matching some portion, but not all, of the gene call), leaving 84 with complete (spanning the entire gene model) EST support (Table 11). Genes in the no-EST support category are of special interest, as they may be specifically expressed *in planta* and thus not expressed under conditions used for preparing our EST libraries (fungus grown *in vitro* on a variety of complete (CM) and minimal (MM) media, and mixed into CM or MM pools). Lack of strong EST support may also suggest an erroneous gene call.

**Table 11:** *C. heterostrophus* strain C5 SSP candidate effector analysis.

**Expression data**	
In complete medium	84
In minimal medium	24
No expression data	72
**Predicted Domains and Conservation**	
None	120
None, but present in other orders	23
PFAM domain predicted	37
**SNPs**	
SNPs in at least one *Cochliobolus* species	101
no SNPs	79

As is typical with candidate effectors, functional domain predictions were lacking, with only 37 candidates having some predicted function, generally involved in cell wall or extracellular matrix function (Table 11, Table S11). An additional 23 candidates were conserved in other fungi outside of the Dothideomycetes. The remaining 120 calls were featureless and seemingly unique to the Dothideomycetes. *C. heterostrophus* strain C5 SSP calls were rich in SNP calls to other *Cochliobolus* genomes: 101 candidate SSPs had SNPs with at least one other *Cochliobolus* genome.

In our all versus all blast analysis, only 6 of the 180 *C. heterostrophus* C5 SSPs were found in all 10 strains examined and 14 were unique to strain C5 (Table 11). The presence or absence of most SSPs did not fall into easily categorized bins such as *C. heterostrophus*-specific, or maize-pathogens only. Instead, SSPs were present and absent in no particular pattern across the genomes. 115 SSPs were present in at least one other species (*C. victoriae*, *C. miyabeanus*, *C. carbonum*, or *S. turcica*), with seven found in all species, and 27 in all *Cochliobolus* species. SSPs mapped to all scaffolds larger than S26 (Figure 2).

Unlike those in some phytopathogens, such as *Leptosphaeria maculans* [72], SSP encoding genes did not occur in clusters; candidates seldom were located within 10 kb of each other (Figure 2). These genes were, however, often located in or near regions we identified as *C. heterostrophus* species unique (Figure 2).

## Discussion

The genomes of five *C. heterostrophus* strains, two *C. sativus* strains, three additional *Cochliobolus* species (*C. victoriae*, *C. carbonum*, *C. miyabeanus*) and *S. turcica*, a member of a close sister genus, were sequenced and compared, to identify unique genomic regions and to inventory secondary metabolism and SSP encoding genes. This dataset is distinctive in that it allows us to contrast genomes that are very closely related, yet differ in several key ways. First, our dataset includes highly related pathogens of several different host plants (corn, wheat, rice, barley, Brachypodium). Second, this set includes pathogens that are highly virulent on specific cultivars of a particular host (i.e., *C. heterostrophus* race T on Tcms maize, *C. victoriae* on *Vb* oats, *C. carbonum* on *hmhm* maize), as well as more generalist pathogens such as *C. sativus* that can cause disease on multiple hosts (barley, wheat, Brachypodium). Third, the group includes two hemibiotrophs, *S. turcica* and *C. sativus*, while the rest are necrotrophs. Fourth, we can make graduated comparisons at different levels of parental and phylogenetic relatedness as we progress from genomes of the same inbred line, species, genus, and family. We have used this final point as a probe to attempt to understand the significant genomic differences between strains that shape host choice, specificity, and lifestyle.

### Structural differences across strains and species

Our whole-genome alignment data support graduated degrees of similarity at the highly inbred strain, field strain, species and genus levels. *C. heterostrophus* strains C4 and C5, offspring of successive backcrosses [42], were highly similar to one another, with 20 fold fewer SNPs than pairwise comparisons of reference strain C5 to *C. heterostrophus* field strains. This remarkably low number of SNPs highlights the power of selective inbreeding in establishing uniformity across the genome. The two *C. sativus* field strains, when aligned to each other, had a comparable number of SNPs to those of *C. heterostrophus* field strains aligned to reference *C. heterostrophus* strain C5. Other *Cochliobolus* genomes had roughly 50 fold more SNPs than *C. heterostrophus* field strains when aligned to the *C. heterostrophus* C5 reference. This level of similarity was seen when comparing any two *Cochliobolus* species to one another, with the exception of comparing *C. victoriae* to *C. carbonum*. These two species are capable of successfully mating, although progeny of crosses are unable to cross to each other or to their parents (Turgeon lab, unpublished). We have hypothesized that *C. victoriae* may have evolved from a *C. carbonum* strain [49]. This similarity is seen at the whole genome level, as *C. victoriae* and *C. carbonum* share an intermediary number of SNPs compared to *C. heterostrophus* inter- and intra-species comparisons.

Our SNP data show that approximately one quarter of the genome differs between *Cochliobolus* species and that only about one tenth of this is found in segments larger than 5 kb. We and others [72], [73], [74] have recently introduced the term mesosyteny [1] to describe organizational conservation between species. Genetic content is conserved across chromosomes, but not co-linearly. It seems possible that our findings here, showing that many small, scattered differences summing to significant quantitative differences (i.e., 25% dissimilar), could be the product of the same mechanisms that result in mesosyntenic patterns. Pathogens of the same host (e.g., *C. carbonum* and *C. heterostrophus* on maize) or lifestyle were not more similar to each other than those of different hosts; instead overarching genetic patterns followed phylogenetic lines. As it is estimated that the Pleosporaceae arose as a group less than 20 MYA (see Figure 1 in Ohm *et al.*, [1]) and the genus *Cochliobolus* is young in the group, genome comparisons provide us with an overall picture of a timeline of how genome diversity varies with speciation.

Our intra-species SNP tallies are comparable to SNP tallies found when strains of other species are examined. For example, there were 10,495 SNPs called between two *Fusarium graminearum* strains [75], and a range of 13,274–188,346 SNPs called for 18 *Neurospora crassa* classical genetic mutants [76]. Both dataset tallies are in the same range as our *C. heterostrophus* field strain comparisons. With respect to SNPs in different species of the same genus, it is unusual that we were able to perform a whole-genome SNP analysis at all, without limiting our scope to coding sequence. We owe this to the very close phylogenetic relationship of these species.

### Strain-, species-, and genus-specific genes for secondary metabolism

Although individual functional domains of *NPS/PKS* proteins can be identified bioinformatically, attempting to predict their corresponding metabolite product is challenging. The genes encoding these proteins evolve rapidly and through complex mechanisms [57], [65] and whole gene alignment methods provide misleading or unclear results when determining presence or absence of a particular *NPS* or *PKS* gene. Here, we extracted individual conserved signature catalytic domains, i.e., the AMP-binding domains from mono- or multi-modular NRPSs or the ketosynthase (KS) domain from multidomain PKSs using customized HMM models, then built alignments and phylogenetic trees with these individual units to determine the presence or absence of whole or partial NRPS and PKS proteins, and their evolutionary relationships. In our opinion this is a necessary first step towards understanding evolutionary history of the corresponding genes and the possible small molecules produced by these highly diverse proteins.

We found that within a Dothideomycete genus, in this case *Cochliobolus*, approximately half of the *NPS* and a third of the *PKS* genes are well conserved (present in all strains). When related *S. turcica* was considered these numbers dropped to a third and a fifth, respectively. The rest were found to be poorly conserved or species-unique when the highly curated *C. heterostrophus* gene sets were used as reference. The small molecules produced by the corresponding non-conserved proteins are largely uncharacterized, but the differences between strains and species imply that the potential for production of biochemically unique molecules is large and considerably beyond that expected for closely related strains (housekeeping genes share ∼95% identity). These findings refine our understanding of *NPS* and *PKS* genes, as very few are conserved. For example, only two *NPS* genes and one *PKS* gene were found when 18 Dothideomycete genomes were analyzed [1]. Broadly conserved secondary metabolism genes, where they have been characterized, produce small molecules that serve basic cellular functions (ferricrocin, melanin [58], [77], [78], [79]). Poorly conserved *NPS* and *PKS* genes, while still largely uncharacterized, can include those involved in host-specific high virulence.

### NPS1, NPS3, and NPS13 embody the intriguing genetic origins of NRPS proteins

NPS1 and NPS3 AMP domains are discontinuously distributed and expanded across the *Cochliobolus* and *Setosphaeria* isolates sequenced and are sources of much of the NRPS diversity (Figure 5, Figure 6 and [57]). The individual AMP modules do not cluster by protein, but instead, NPS1, NPS3 and NPS13 AMP domains occur in two distinct, and mixed, groups (Figure 6). Strikingly, each *Cochliobolus* species possesses either a complete *C. heterostrophus* NPS1 or NPS3 ortholog, but never both. Furthermore, all species have one or more additional NRPS proteins consisting of NPS1/NPS3 related domains, and a NPS13 related domain that is absent from all *C. heterostrophus* genomes except Hm540 (Figure 5). The *C. heterostrophus* Hm540 genome includes all four corresponding genes: *NPS1*, *NPS3*, *NPS13*, and the additional *NPS1/NPS3/NPS13* gene (Figure 5). The pattern of duplication and loss appears to have been very rapid to account for this distribution, and is further complicated by the presence of additional bi-, mono-, tri-, and tetra-modular proteins, particularly in *C. sativus*, whose AMP domains group with NPS1/NPS3/NPS13 proteins, (Figure 5, Figure 7, Figure S5).

### Evolutionary origin of *NPS* and *PKS* genes

The origin of strain or species unique secondary metabolism genes is of great interest and horizontal gene transfer is a common, but not the only, explanation for their appearance [65], [80], [81], [82]. The volatility of the *NPS1*, *NPS3* and *NPS13* family raises the possibility that genes we presume are horizontally transmitted could have vertical histories obfuscated by species and strain sequence depth. We speculate that partial or whole genes encoding individual domains or whole proteins recombine and expand quickly, and, when they confer high virulence, as in the case of HSTs, can spread rapidly throughout a population. When the susceptible host allele is not present in the population, the gene is lost or not conserved in the majority, but not the entirety, of the population, as is the case for *C. heterostrophus* race T and genes for T-toxin production; race T is difficult to find in the field currently [43]. As we sequence more and more isolates, we might find that the T-toxin genes are present in strains of many more Dothideomycetes than we originally expected. This is certainly the case with the HC-toxin genes which is not so surprising, given that the *Hm* alleles are present in most plants.

### Pinpointing virulence-associated secondary metabolite genes

Identifying secondary metabolites that function as virulence factors (such as HSTs) is a primary goal when studying a pathogen's genome. The impact of HSTs was realized early on because they render the producing fungi pathogenic or highly virulent to principal crops. Thus, most were characterized physiologically and genetically decades ago [4], [18], [19], [40], [83], [84], [85]. The pivotal point of our comparative analyses is the strikingly obvious observation that secondary metabolite genes, when unique to a species or strain, are likely to encode a virulence determinant. We provide several examples.

The first example is the *C. heterostrophus PKS1* and *PKS2* genes involved in production of the HST T-toxin. These genes reside in 1.2 Mb of DNA, not found in race O and located at the breakpoints of two race T chromosomes (12;6, 6;12), reciprocally translocated with respect to race O counterparts (chromosomes 6, 12). The T-toxin genes are not in race O or any other *Cochliobolus* species. Deletion of either *PKS* eliminates T-toxin production and drastically reduces virulence of the fungus on Tcms maize, as reported earlier [45], [67]. Known *Tox1* genes, such as *PKS1* are on very small scaffolds (∼25 kb) in race T strains C4, Hm338, and PR1x412 (Figure 11), which cannot be further assembled due to the repetitive and AT-rich nature of the locus. Thus the physical structure of the *Tox1* locus remains elusive, but its association with a unique genomic region, however complex, is clear-cut. Although T-toxin is unique to *C. heterostrophus*, a closely related fungus, *Didymella zeae maydis* (formerly, *Phyllosticta maydis*, *Mycosphaerella zeae maydis*), produces a polyketide HST, PM toxin, with the same biological specificity as T-toxin. The central PKS for PM-toxin is the closest PKS to *C. heterostrophus* PKS1, but still only ∼60% identical at the amino acid level and organization of the cluster of genes required for toxin production differs; in *D. zeae-maydis*, the genes are present in a single tight cluster [86], [87].

The second example, examined here, is the NRPS, HTS1, for HC-toxin production. The genes for HC-toxin produced by *C. carbonum*, were identified two decades ago in a tour de force molecular manipulation exercise [88], [89]. A strong genomic signature attends these genes as they reside in an ∼600 kb region not found in other races of *C. carbonum*, or in any of the additional *Cochliobolus* genomes examined then or here. Two copies of a cluster of HC-toxin genes are located in this region and both copies of the core NRPS, HTS1, had to be deleted to demonstrate elimination of toxin production and reduction of virulence [88], [89]. In current investigations, we, Manning et al., [61] and Wight and Walton [62] have discovered that HC-toxin like genes are present in *S. turcica* (Figure 8), *P. tritici-repentis* and *A. jesenskae*, respectively. These genes are also apparent orthologs of the genes for apicidin (APS1) production by some *Fusarium* species [90]. Thus, genes for HC-toxin or HC-toxin-like metabolites are more broadly distributed than previously thought. In terms of amino acid identity, the *S. turcica* and *P. tritici-repentis* NRPS HTS1 proteins are 79% identical at the amino acid level, but identity drops to 39–43% when these are compared to the *C. carbonum* HTS1 or APS1 proteins. The *S. turcica* and *P. tritici-repentis* HTS1 orthologs lack the C-terminal condensation domain found in *C. carbonum* HTS1 and *F. semitectum* APS1, suggesting *S. turcica* and *P. tritici-repentis* make a different product. Whether or not *S. turcica* and *P. tritici-repentis* are capable of producing HC-toxin is unknown, however, it has been reported [62] that *A. jesenskae* does. That HC-toxin producing capability might be found in pathogens other than *C. carbonum* is not unreasonable, considering the maize defense gene *Hm1*, necessary to detoxify the toxin, is found in all grasses [91].

The third example concerns *C. sativus*. Two of the *NPS* genes unique to *C. sativus* ND90Pr (IDs 115356 and 140513, Figure 7) are present at the *VHv1* locus associated with high virulence on cultivar Bowman. The entire *VHv1* locus is absent in the low virulence isolate, ND93-1, and the two *NPSs* are not found in any other genomes examined here, or in Genbank. Our data show that these genes are up-regulated 12 hrs after inoculation, and that deletion of one of them, (115356), significantly reduces virulence on barley cultivar Bowman (Figure 7). Recent work on deletion of the gene encoding 4′-phosphopantetheinyl transferase provided indirect evidence that a secondary metabolite is involved in the biosynthesis of the virulence factor in ND90Pr [92]; our current work directly confirms this.

The phylogenetic location of these *VHv1 NPS* genes is revealing in that they are either in branches with no close sister members (ID# 140513, Figure S5A) or in the NPS1/NPS3/NPS13 expansion clade (ID# 115356, Figure 5, Figure 6). In addition to these two genes, we have evidence based on real time expression data on RNA from inoculated barley, that the *C. sativus* genes corresponding to protein ID#s 49884 and 130053 are also up-regulated at 12 hrs post inoculation. These genes are found in the same *C. sativus* specific clade (New_8, Figure 3, Figure S5A) as protein ID# 140513, and although we have not yet made mutants, our prediction is that these will also contribute to virulence.

Our analyses of the hemibiotroph, *S. turcica*, is in its infancy, however, as noted in the Results we have identified 13 unique PKSs, three of which (ID# 161586, 30113, 34554) grouped together in a *S. turcica*- specific clade, called ‘New_10’ (Figure 10, Figure S8, Table S10). As preliminary support for the importance of unique *PKS*s, we used real time PCR, to examine *in planta* expression of one of these *S. turcica* unique genes (161586) and found that expression was indeed increased (>500 fold) by three days post inoculation. Although we haven't yet deleted this gene, it is tempting to predict that the *in planta* expression pattern is indicative of a role in virulence.

One of the species-unique NRPSs in *C. victoriae* (on node 1179, gene #7087) is a NRPSs with two AMP domains clustering with 99–100% bootstrap support to AMP domains from the bimodular *A. fumigatus* NRPS, GliP, which produces the ETP toxin, Gliotoxin. Related to these NRPSs is the *L. maculans* NRPS, SirP, which produces sirodesmin. Candidate orthologs of these NRPSs have been reported in *Chaetomium globosum*, *Magnaporthe oryzae*, and *Fusarium graminearum* [93]. Gliotoxin is associated with virulence of *A. fumigatus* to immune-compromised patients [94]. Functional characterization of the newly discovered *C. victoriae* counterpart is necessary to determine the type of ETP produced and whether or not it might play a role in virulence, as Gliotoxin does. Note the entire Gliotoxin gene cluster [64] is present in *C. victoriae* (Figure 9). Gene knockout and screening for alteration in virulence to oats, due to victorin production, indicates no change from that of wild type (Wu, Turgeon, unpublished). This *C. victoriae* NRPS is not found in other *Cochliobolus* genomes, yet it clusters with *A. fumigatus* GliP, exemplifying the patchy distribution signature of most members of the *NPS* family of genes.

### Effectors and lifestyle

Effectors are pathogen produced small secreted proteins (SSPs)/small molecules that interact with the host plant to promote disease. Effectors historically were called avirulence proteins (as their discovery hinged on association with a corresponding plant resistance gene), but we now recognize that effectors are virulence factors that aid the pathogen by specifically targeting aspects of host cell defense and recognition. Evading detection is a necessary strategy for (hemi)biotrophs, where triggering the host hypersensitive response curtails disease. Necrotrophs, on the other hand, benefit from the death of host cells, and have evolved molecules such as HSTs, like victorin which subverts function of an R gene (Lov1/Pc-2) to trigger susceptibility and plant cell death intentionally [14], [15], [95]. Protein HSTs such as *P. tritici-repentis* and *Stagonospora nodorum* ToxA are clear examples of secreted, necrotrophic, proteinaceous, host-selective virulence factors acting to effect virulence in host cells, like any other effector, but which, in the presence of a R-protein look-alike, is necessary for susceptibility [96]. The lingering question is whether or not necrotrophs utilize SSP effectors in the traditional (and difficult to identify) sense of micromanipulation of the host environment, or, instead, use effectors to trigger host cell death through abuse of (hemi)biotrophic defenses. In this regard, given our clear discovery that at least one NRPS metabolite (ID# 115356), when deleted has a much reduced phenotype reminiscent of a necrotrophic HST phenotype, we question whether *C. sativus* should truly be considered a hemibiotroph or a necrotroph.

On the other hand, our SSP analysis shows that *C. sativus* and *S. turcica* have an expanded SSP repertoire compared to the other species examined, which is consistent with a hemibiotroph strategy, i.e., arsenals of effectors are used to evade host detection. The repertoire of candidate effectors in necrotrophs, nevertheless, is quite large. If only a small subset of these is involved in virulence, it would mean that *Cochliobolus*, and perhaps other necrotrophs, use effectors more expansively than is recognized. This is a difficult question to address, and our *in silico* analysis requires experimental confirmation of *in planta* expression and secretion before we can be sure *Cochliobolus* species utilize protein effectors. Perhaps a strategy prioritizing species or strain unique regions would aid characterization attempts. Bearing on this point, the SSP catalogue differed markedly from secondary metabolites in their conservation across, and within, species. Only six of the 180 (3%) *C. heterostrophus* C5 SSPs were identified in all genomes examined (including *S. turcica*), unlike the 7/25 (28%) PKSs and 6/14 (43%) NRPSs. Considering *C. heterostrophus* genomes only, 27 of these 180 SSPs were present in each (15%), again, far fewer than the 21/25 (84%) PKS and 13/14 (93%) NRPS *C. heterostrophus* C5 genes conserved throughout all *C. heterostrophus* genomes. This indicates that, more so than secondary metabolite genes, SSP encoding genes are extraordinarily volatile in the evolutionary history of the genus.

### Final thoughts

The stories of the SCLB and Victoria blight epidemics are dramatic examples of interactions between crops, whose ‘evolution’ is driven by human intervention (breeders) and their pathogens, which evolve naturally to exploit new genetic susceptibilities. Both the Tcms and *Pc-2* genes were introduced into maize and oats, respectively, by breeders fewer than 30 years before the epidemic outbreaks. Specifically, Tcms was discovered in the 1940's, incorporated into elite corn inbred lines increasingly throughout the 1960's, and was present in almost all of the hybrid corn in the US by 1970. The vast monoculture of Tcms maize was the perfect host for the previously unknown race. Species of *Cochliobolus* spp. clearly have proven their ability to cause extraordinary crop losses. As we begin to understand the intimidating capacity for diverse production and evolution of new HSTs, we must also look for ways to apply this knowledge to our disease response strategies.

## Materials and Methods

### Strains

*C. heterostrophus* strains sequenced by JGI included inbred strains C5 (ATCC 48332, race O, *MAT1-1*, *Tox1−*) and C4 (ATCC 48331, race T, *MAT1-2*, *Tox1+*) and field strains Hm540 (geographical origin North Carolina, race O, *MAT1-1*, *Tox1−*), Hm338 (New York, race T, *MAT1-2*, *Tox1+*, ATCC 48317), and PR1x412, (a progeny of a cross between PR1C from Poza Rica, Mexico and strain 412, unknown geographical origin, race T, *MAT1-1*, *Tox1+*). In addition, the genomes of *C. victoriae* strain FI3 (unknown geographical origin, *MAT1-2*, victorin*+*), *C. carbonum* strain 26-R-13 [*MAT1-1*, HC-toxin*+*, a progeny of a cross between *C. carbonum* strains 2-R-6 (*alb2*; *MAT1-1*) and Five Points (unknown geographical origin, *MAT1-2*) performed by Dr. Steve Briggs]. *C. miyabeanus* strain WK1C (Wuankuei, Yulan county China, *MAT1-2*), *C. sativus* isolate ND90Pr (North Dakota, ATCC 201652, *MAT1-2*, pathotype 2 on barley cv. Bowman) and *S. turcica* strain St28A (New York, race 2,3,N, *MAT1-1*) were sequenced by JGI. *C. sativus* isolate ND93-1 (North Dakota, ATCC 201653, *MAT1-1*, pathotype 0 on barley cv. Bowman) was sequenced at the University of Hawaii.

### Genomic resources

The highly inbred *C. heterostrophus* reference race O lab strain C5 was sequenced using the Sanger whole genome shotgun approach, with paired end reads and improved by manual finishing and fosmid clone sequencing (http://www.jgi.doe.gov/sequencing/protocols/prots_production.html). Four different sized libraries were sequenced: 3.1 kb, 6.8 kb, and two fosmid libraries (32.3 kb and 35.3 kb), to a total coverage of 9.95×. ESTs were generated by growing strains in complete and minimal medium under many conditions [on complete and minimal medium, on sexual reproduction plates, stress medium (-N, -Fe, etc)] and pooled as complete or minimal samples for sequencing and support of gene annotation. The genome of isogenic *C. heterostrophus* race T strain C4 was sequenced using Illumina technology (300 bp insert size, 2×76 bp reads to a nominal depth of 200×), assembled using Velvet [97] and AllPathsLG [98] and annotated using ESTs from *C. heterostrophus* strain C5. The genome of *C. sativus* pathotype 2 isolate ND90Pr was sequenced using a hybrid approach, which included 40 kb fosmid Sanger reads, shredded consensus from Velvet assembled Illumina data (300 bp insert size, 2×76 bp reads), Roche (454) standard and Roche (454) 4 kb insert paired ends, all assembled using Newbler [99] and annotated using *C. sativus* ND90Pr ESTs as described below. The genome of the second *C. sativus* isolate, ND93-1, was sequenced at the University of Hawaii by paired end 454 runs and assembled using Newbler. *S. turcica* strain 28A was sequenced using Roche (454), Sanger fosmids, and shredded consensus from Velvet assembled Illumina data; EST libraries were prepared from *S. turcica* strains using conditions described above for *C. heterostrophus*.

The JGI annotation pipeline was used to annotate *C. heterostrophus* strains C5 and C4, *C. sativus* ND90Pr, and *S. turcica*. For this, the assembled genomic scaffolds were masked using RepeatMasker [100]with the RepBase fungal library of 234 fungal repeats [101] and genome-specific libraries derived using [102]. Multiple sets of gene models were predicted for each assembly, and automated filtering based on homology and EST support was applied to produce a final non-redundant GeneCatalog representing the best gene model found at each genomic locus. The gene-prediction methods were: *EST-based predictions* with EST map (http://softberry.com) using raw ESTs and assembled EST contigs for each genome; *homology-based predictions* with Fgenesh+ [103] and Genewise [104], with homology seeded by BLASTx alignments of the GenBank non-redundant sequence database (NR: http://www.ncbi.nlm.nih.gov/BLAST/) to the genomic scaffolds; and *ab initio predictions* using Fgenesh [103]) and GeneMark [105]. Genewise models were extended to include 5′ start and/or 3′ stop codons when possible. Additional EST-extended sets were generated using BLAT-aligned [106] EST data to add 5′ UTRs, 3′UTRs, and CDS regions that were supported by ESTs but had been omitted by the initial prediction methods.

All genome annotations can be interactively accessed through MycoCosm [107], http://jgi.doe.gov/fungi.

### Resequencing additional *C. heterostrophus* race T and race O strains and other *Cochliobolus* spp

Because the subject genomes are all closely related to the fully sequenced reference *C. heterostrophus* strain C5, additional *C. heterostrophus* field strains Hm540, Hm338 and PR1x412, *C. victoriae*, *C. carbonum*, and *C. miyabeanus*, were (re)-sequenced using using Illumina technology. DNA was randomly sheared into ∼200 bp fragments using Covaris E210 according to the manufacturer's recommendation and the resulting fragments were used to create an Illumina library. 2×76 bp reads were assembled using Velvet (version 1.1.04), [97]. There are no ESTs available for these organisms. Assembled contigs were mapped to the reference *C. heterostrophus* C5 for analysis of genome variation and rearrangements. Assembled reads are called ‘nodes’ (scaffolds). Overall sequence assembly and annotation statistics are presented in Table 3.

The genomes without JGI annotation pipeline gene predictions (*Ch*Hm540, *Ch*Hm338, *Ch*PR1x412, *C. carbonum*, *C. miyabeanus*, *C. victoriae*) were annotated using Augustus [108].

### Mapping genomes to the *C. heterostrophus* strain C5 assembly

Assembled genomes were mapped individually to the C5 reference strain using the nucmer program of MUMmer v 3.22 [46], a program that finds unique, exact matches to build whole genome alignments. SNPs were called and analyzed using the dnadiff wrapper on the filtered MUMmer delta files. Unassembled reads were also aligned to the *C. heterostrophus* C5 reference genome for low coverage analysis using maq-0.7.1. Regions of the reference genome under a depth of three aligned reads were considered “low coverage” for our analyses. Adjacent low coverage regions were merged if they were separated by less than 100 bp in order to minimize noise from mis-mapping of occasional low quality reads. After low coverage regions were identified for pairwise comparisons to C5, regions were identified that were shared as low coverage in multiple genomes: i.e., *C. carbonum*, *C. victoriae*, *C. miyabeanus*, and *C. sativus* for *C. heterostrophus* specific regions; *C. heterostrophus* Hm540, Hm338, and PR1x412 for C strain specific regions; and C4, Hm338, and PR1x412 for race O specific regions.

Cross genome comparisons were visualized using Mauve [44], a multiple genome alignment tool that visualizes localized collinear blocks (LCB) between genomes.

### Mapping reference genome *C. heterostrophus* strain C5 sequenced scaffolds onto the genetic map

Cloned RFLP markers [8] were sequenced and sequences used in blast queries against the *C. heterostrophus* C5 assembly. Top hits were filtered using Bioperl and manually confirmed to span the entire RFLP with very high stringency to rule out markers that might exist as more than one copy. Physical and genetic distances of adjacent RFLPs mapping to the same scaffold were plotted and used to calculate an average ratio of physical to genetic distance (Figure S1, Table S2). Relative RFLP location was used to orient scaffolds along the linkage group when possible.

### Mapping of sequenced scaffolds to the *C. sativus* genetic map

A genetic linkage map was generated previously using the mapping population derived from a cross between parental isolates ND93-1 and ND90Pr [30] and amplified fragment length polymorphism (AFLP) and RFLP markers. To add simple sequence repeat (SSR) markers to the map, the draft sequence assembly of the *C. sativus* isolate ND93-1 was screened for SSR loci with di- and tri-nucleotide units tandemly repeated six or more times using a Perl script (provided by Zheng Jin Tu at the University of Minnesota, St. Paul). The SSR-containing sequences from ND93-1 were aligned to the draft genome sequence of isolate (ND90Pr) of *C. sativus*. Only those sequences that were polymorphic between the two *C. sativus* parents (ND93-1 and ND90Pr) were used for primer design and tested for segregation in the mapping population used previously [30]. PCR conditions and detection of SSR markers were as previously described [109]. Map construction was performed by using MAPMAKER version 2.0 [110]. A minimum LOD value of 4.0 and a maximum theta of 0.3 were used to group all SSR markers with previously mapped AFLP, RFLP and PCR markers [30]. The Kosambi mapping function was used to calculate the map distance.

To associate linkage groups to the sequenced scaffolds of *C. sativus*, the sequences of mapped SSR markers were used as queries to blast against the draft genome assembly of *C. sativus* isolate ND90Pr and the coordinates of each SSR marker were recorded for the associated scaffold.

### Identification and phylogenomic characterization of nonribosomal peptide synthetases and polyketide synthases

NRPS and PKS proteins were identified using our custom fungal AMP domain model [57] for the former and an HMM model build from *C. heterostrophus* KS domains plus sequences from the C-terminal and N-terminal ketosynthase (KS) Pfam domains for the latter (PF00109 and PF02801). Proteins were identified in two ways. In the first case, genome nucleotide sequences were searched using Genewise [111] and sequences extracted and concatenated by a Perl script utilizing Bioperl's searchIO system [112]. In the second case, Augustus protein models (see above) and JGI protein models (*C. heterostrophus*, *C. sativus*, *S. turcica*) were searched with the PKS KS and NRPS AMP HMM using HMMER 3.0 [113], and the sequences extracted and concatenated using a Perl script with Bioperl's searchIO.

AMP and KS domains were aligned, separately, with MAFFT (http://mafft.cbrc.jp/alignment/software/) and manually inspected to remove columns of poor alignment. ProtTest [114] was run on both alignments and identified the RTREVF model as the best fit for the AMP domains and the WAGF model as the best fit for the KS domain alignments, respectively. RAxML [115] using the RTREVF and WAGF models with a gamma distribution was used to infer maximum likelihood trees and bootstrap support was determined using the fast-bootstrap method with 1000 bootstrap replicates [116]. The CIPRES portal (http://www.phylo.org/sub_sections/portal/) was used for inference of phylogenetic trees.

*C. sativus* ND90Pr NRPS AMP domains were used as blast queries to identify AMP domain orthologs in ND93-1 with the methods above. An ND93-1 AMP was considered orthologous if it was at least 95% identical to the ND90Pr query.

### Small secreted protein identification

Candidate small secreted proteins (SSP) were identified by screening the gene catalogue of each genome. Proteins smaller than 200 amino acids and containing more than 2% cysteines were searched for transmembrane domains and secretion tags using Phobius [71]. Those without transmembrane domains were retained. EST support and domain prediction for *C. heterostrophus* C5 SSPs was performed using the JGI portal. Cross-genome comparisons were made based on all vs. all reciprocal best hit analysis with an 80% similarity cutoff.

### *C. sativus* EST library construction

Three EST libraries were constructed. A mycelia-only library was constructed by harvesting mycelia grown on different media including Potato Dextrose Agar (PDA), minimal medium (MM) [117], V8PDA (150 ml V8 juice, 850 ml H_2_O, 10 g PDA, 10 g Agar and 3 g CaCO_3_), and water agar (15 g agar, 1000 ml water). Mycelia were harvested after 12, 24, 48, 76 and 96 hours of growth, and time points from different media were mixed together for RNA extraction. Equal amounts of extracted RNA from each of the 5 time points was bulked to construct the mycelia only library To construct the *in planta* cDNA libraries, two week old barley cv. Bowman and 4 week old *Brachypodium distachyon* line Bd21were inoculated with conidia of ND90Pr at a concentration of 5×10^3^/ml [118]. Inoculated plants were incubated in a humid chamber for 24 hours and moved to the greenhouse. Leaves were harvested at 6, 12, 24, 48, 72, and 96 hours after inoculation and total RNA extracted from each sample. The final *in planta* cDNA libraries were constructed by mixing the equal amounts of total RNA from different time points. Total RNA was isolated from all samples using the PureLink RNA Mini Kit (Invitrogen, Carlsbad, CA) and purified by treatment with DNase I (Invitrogen, Carlsbad, CA). These three libraries were sequenced by JGI.

### Quantitative real-time PCR

For *C. sativus*, total RNA extracted as described above at six time points (6, 12, 24, 48, 72, and 96 hours) after inoculation was used for RT-PCR. The reverse transcription reaction was performed on 2 µg of total RNA using the SuperScript III First-Strand Synthesis System (Invitrogen, Carlsbad, CA). cDNA was diluted 20 times and used as the template for quantitative RT-PCR, which was performed with the AB7500 real time PCR system (Applied Biosystems, Foster, CA) (Table S9). For each cDNA sample, three replications were performed. Each reaction mixture (20 µl) contained 5 µl of the cDNA template, 10 µl of SYBR Green PCR Master Mix (Applied Biosystems, Foster, CA) and 0.3 µl of each primer (10 mM). All samples were normalized using RT-Actin-F and RT-Actin-R primers as a control, and values were expressed as the change in the increase/decrease of the relative levels of the control sample (M96, which is the mixture of mycelia harvested from different media including PDA, MM, V8PDA, and water agar).

For *S. turcica*, leaf samples with lesions were collected at five time points (3, 5, 6, 7 and 8 days) after inoculation with 9×10^4^ spores per plant (three weeks old) and total RNA was extracted and qPCR done as described [119]. The actin gene was used as internal control using *ATC1* primers [119]. The *S. turcica* gene primers corresponding to protein ID 161586 are listed in Table S9. Expression level was expressed as fold change versus mycelial samples harvested on Lactose Casein Agar (LCA) plates.

### Transformation and gene deletion

Fungal transformation and molecular characterization of gene knockout mutants were conducted according to the methods of [120]. The split marker system [121] was used for gene deletion. The 5′ and 3′ flanking sequences of the *NPS* gene encoding protein ID 115356 were amplified from ND90Pr DNA using primer pairs GTCGACTGCCATCTGGAAAC/CACTGGCCGTCGTTTTACAACGTCCACTCGACAGGTCCGTAGGT and TCATGGTCATAGCTGTTTCCTGTGGTATCCACAAAGCCACAGCA/GACGAACCAGA GATGCATGA) respectively.

To verify deletion of the gene corresponding to protein ID 115356, primers CAN1-F3: AGTTGTTGGGGAGTTGTTGG and CAN1-F4: TGAGCCGTTGTCATGTATCG matching the deleted portion of the gene were used. The expected PCR product was obtained from WT DNA, but not when DNA of the deletion mutant was used as template. To further confirm that the hygromycin resistance gene replaced the target gene at the native locus, PCR was conducted using a primer located outside the 3′ flank used for gene deletion (CsNPS1-F0: GTCCTACGGCAATTGTGGAC) and a second primer (HY: GGATGCCTCCGCTCGAAGTA) located in the hygromycin resistance gene. No amplification occurred when WT DNA was used, while the expected amplicon was observed when DNA of the mutants was used as template.

### Plant inoculation

For *C. sativus*, virulence of the mutant (ID# 115356, Figure 7C) and wild type strains was tested on barley cv. Bowman by spray inoculation as described in Fetch and Steffenson (1999), except 2×10^3^ conidia/ml were used. Inoculated plants were incubated in a humid chamber for 18–24 hours, and then transferred to a greenhouse room (20+/−2°C).

For *S. turcica*, three week old W64A maize plants were sprayed with 9×10^4^ spores per plant, and plants grown under conditions described previously for *C. heterostrophus* [119].

### Mating type locus comparisons

*MAT1-1* and *MAT1-2* mating type regions were identified by blasting the corresponding known *C. heterostrophus MAT* sequences (*MAT1-1*: accession CAA48465, *MAT1-2*: accession CAA48464) against each genome. Regions immediately 10 kb upstream and downstream were aligned pairwise to *C. heterostrophus* C5 (*MAT1-1*) or C4 (*MAT1-2*) *MAT* regions using ProgressiveMauve [44] to generate SNP data, and as a group (with and without *S. turcica* for *MAT1-1*) for visualizing the alignment.

### Data access

Genome assemblies and annotations are available via JGI Genome Portal MycoCosm (http://jgi.doe.gov/fungi, [107] and DDBJ/EMBL/GenBank under the following accessions *Cochliobolus heterostrophus* ATCC 48331 (race T, strain C4): AIHU00000000, *Cochliobolus heterostrophus* ATCC 48332 (race O, strain C5): AIDY00000000, *Cochliobolus sativus* ND90Pr: AEIN00000000, *Setosphaeria turcica* Et28A: AIHT00000000, *C. sativus* ND93-1:PRJNA87041, *Cochliobolus carbonum* 26-R-13: AMCN00000000, *Cochliobolus miyabeanus* ATCC 44560: AMCO00000000, *Cochliobolus victoriae* FI3: AMCY00000000.

## Supporting Information

Figure S1 Genetic distance correlates with physical distance on the *C. heterostrophus* map. RFLP markers located on the same scaffold were used to plot genetic distance against physical distance. Genetic distances between RFLPs determined by Tzeng et al. [8].
(PDF)

Figure S2 A *C. heterostrophus* dispensable chromosome is present in some but not all *C. heterostrophus* strains. Mauve alignment [44] of the genome of strains Hm540 and C5. Colored blocks [Locally Collinear Blocks (LCB)] indicate matches between genomes. Note there are only a few matches (colored blocks) in Hm540 to scaffold S16/chromosome B1 in C5. The C5 S16 scaffold (∼750 kb) is shown in its entirety below the whole genome alignment.
(PDF)

Figure S3 *C. sativus* SSR sequences anchor sequenced scaffolds to the genetic map. Genetic map of *C. sativus* based on 68 SSR markers, 102 amplified fragment length polymorphism (AFLP) markers, 34 RFLP markers, two polymerase chain reaction–amplified markers, the mating type locus (*CsMAT*), and the barley cultivar-specific virulence locus (*VHv1*). Of the 37 linkage groups, 30 were assigned to 16 of the 157 scaffolds based on alignment of mapped SSR markers to the sequence assembly of ND90Pr. Linkage groups are on the left (open bars, numbered at the top) and assembled scaffolds (solid black bars, numbered at the top) are on the right. The start point of each scaffold is at the top. Dotted lines connect the SSR loci on the genetic map and physical map. AFLP markers flanking the *VHv1* locus and the locus itself are highlighted in red (Figure 7). The scale bar corresponds to 500 kb.
(PDF)

Figure S4 Analysis of the mating type region in *Cochliobolus* spp. and *S. turcica. S. turcica* 28A, *C. sativus* ND90Pr, *C. carbonum* 26-R-13, *C. heterostrophus* Hm540, and *C. heterostrophus* PR1x412, and the reference *C. heterostrophus* C5 strains are *MAT1-1*, while the others are *MAT1-2*. 10 kb regions flanking the *MAT* idiomorphs were aligned for each mating type. In all cases, the order of genes immediately surrounding the *MAT* locus (∼20 kb) was conserved, as described in [123] and [124]; genes flanking the *MAT* locus, differ from those flanking *MAT* in other ascomycetes [125]. JGI ID numbers are shown for strain C5 (*MAT1-1*) and C4 (*MAT1-2*). *S. turcica* had the most variation compared to the *Cochliobolus MAT* region, although the *MAT* genes were well conserved. Approximately 500 bp of the 5′ region and ∼3 kb of the 3′ region around the *MAT* gene were more variable than other regions when all genomes were compared.
(PDF)

Figure S5 Maximum likelihood tree of NRPS AMP-binding (AMP) domains identified using Augustus (A) and Genewise (B). RAxML using the RTREVF model with a gamma distribution was used to infer the maximum likelihood tree and bootstrap support was determined using the fast-bootstrap method with 1000 bootstrap replicates. See Materials and Methods. AMP domains are color-coded by species. Plain branches at the top of the tree are AMP domains from related adenylating enzymes (e.g., acyl CoA ligases, etc). AMP domains corresponding to the *C. heterostrophus* reference set (Figure 3, Table 4, Table S6,) are indicated on the right of each group. Bootstrap values above the branches. Certain additional AMP domains correspond to well-known metabolites from fungi outside the *Cochliobolus* and *Setosphaeria* strains. *Gibberella fujikuroi* FusS produces fusarin C, *Fusarium heterosporum* EqiS produces Equisetin, *Aspergillus fumigatus* GliP produces Gliotoxin, *Leptosphaeria maculans* SirP produces sirodesmin, *Magnaporthe oryzae* Ace1 produces an unknown metabolite involved in pathogenicity, *Fusarium* spp. Esyn1 produces enniatin, AAR, involved in lysine biosynthesis, *Tolypocladium inflatum* TiSimA produces cyclosporin, Penicillium spp., produces penicillin, δ-(L-α-aminoadipyl)-L-cysteine-D-valine (ACV), *C. carbonum* HTS1 produces HC-toxin, *Hypocrea virens* Tex1 produces peptaibols, *Alternaria alternata* AMT produces AMT toxin, *Claviceps purpurea* PS1(LPS1) is an NRPS that along with lysergic acid, produces an ergot alkaloid, *Metarhizium anisopliae* PesA unknown product, *Epichloë* festucae perA produces Peramine.
(PDF)

Figure S6 Maximum likelihood tree of PKS ketosynthase (KS) domains identified using Augustus (A) and Genewise (B). RAxML using the WAGF model with a gamma distribution was used to infer the maximum likelihood tree and bootstrap support was determined using the fast-bootstrap method with 1000 bootstrap replicates. See Materials and Methods. Plain branches at the top of the tree are KS domains from related enzymes. KS domains are color-coded by species as in Figure S6. Bootstrap values above the branches. PKS11 is a *C. heterostrophus* ortholog of the *Fusarium verticillioides* PKS for fumonisin.
(PDF)

Figure S7 Quantification of spot blotch disease induced by the *C. sativus* wild type and mutant (Δ115356) on barley cv. Bowman. Disease rating was taken at 7 days after inoculation and is based on a 1 to 9 scale [118]. Four replicates were used. Error bar indicates the standard deviation.
(PDF)

Figure S8 Quantitative real-time PCR analysis of *S. turcica PKS* gene (protein ID 161586) during infection of maize cultivar W64A-N. Gene expression was normalized based on the expression of the β-actin gene. Values are relative expression levels compared to that in mycelia grown on LCA medium. Samples were collected at 3, 5, 6, 7, and 8 days after inoculation. Primers are shown in Table S9. Error bars indicate the minimum and maximum relative expression values of the gene.
(PDF)

Table S1 Comparison of estimates for *C. heterostrophus* strain C5 chromosome sizes based on CHEF gel analysis and assembly size.
(DOC)

Table S2 RFLP map coordinates on the C5 assembly.
(XLS)

Table S3 SNPs across species detected with Mummer.
(XLS)

Table S4 MUMMER MAT alignments.
(DOC)

Table S5 Regions of low coverage of C5, by other sequences.
(DOC)

Table S6 Master NRPS AMP domain inventories.
(XLS)

Table S7 Forty three predicted genes in the unique region of isolate ND90Pr associated with the virulence locus *VHv1*.
(DOC)

Table S8 Summary of repetitive elements identified in the unique region of isolate ND90Pr.
(DOC)

Table S9 Primers used in quantitative real time-PCR of *C. sativus* and *S. turcica* genes.
(DOC)

Table S10 Master PKS KS domain inventories.
(XLS)

Table S11 Master SSP inventories and associated SNPs.
(XLS)

Text S1 Supplementary Methods. Mapping scaffolds onto the *C. heterostrophus* genetic map. Mapping scaffolds to the *C. sativus* genetic map. Mating type region comparisons.
(DOCX)
