# Supplementary figures and images for: Comparative Genome Structure, Secondary Metabolite, and Effector Coding Capacity across Cochliobolus Pathogens
Source: PLoS Genet. 2013 Jan 24;9(1):e1003233. doi: 10.1371/journal.pgen.1003233 (PMC3554632; doi:10.1371/journal.pgen.1003233)

## genetic vs physical distance

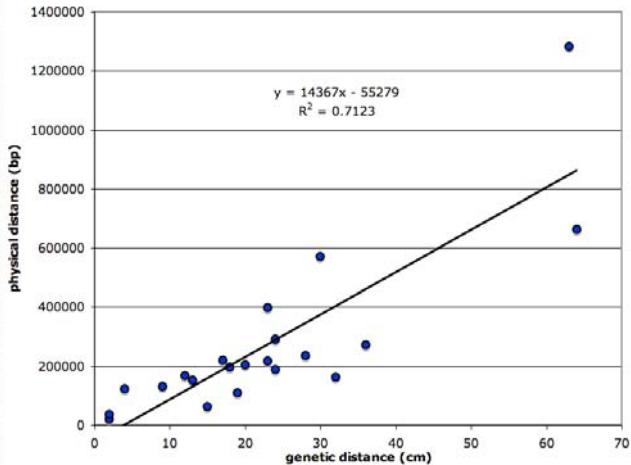

**Figure S1**

Supplement: Figure S1 — Genetic distance correlates with physical distance on the C. heterostrophus map. RFLP markers located on the same scaffold were used to plot genetic distance against physical distance. Genetic distances between RFLPs determined by Tzeng et al. [8]. (PDF) [file pgen.1003233.s001.pdf]

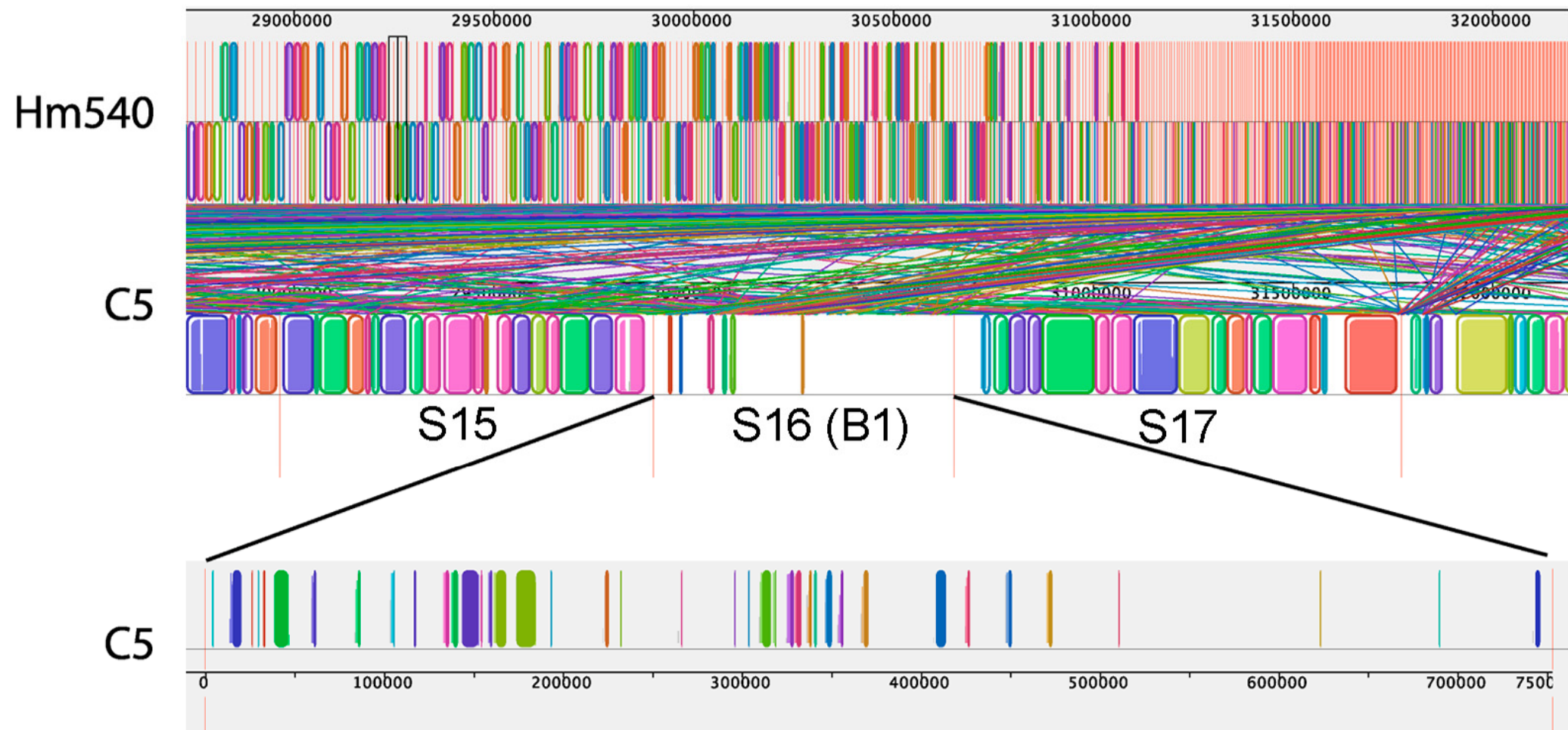

**Figure S2**

Supplement: Figure S2 — A C. heterostrophus dispensable chromosome is present in some but not all C. heterostrophus strains. Mauve alignment [44] of the genome of strains Hm540 and C5. Colored blocks [Locally Collinear Blocks (LCB)] indicate matches between genomes. Note there are only a few matches (colored blocks) in Hm540 to scaffold S16/chromosome B1 in C5. The C5 S16 scaffold (∼750 kb) is shown in its entirety below the whole genome alignment. (PDF) [file pgen.1003233.s002.pdf]

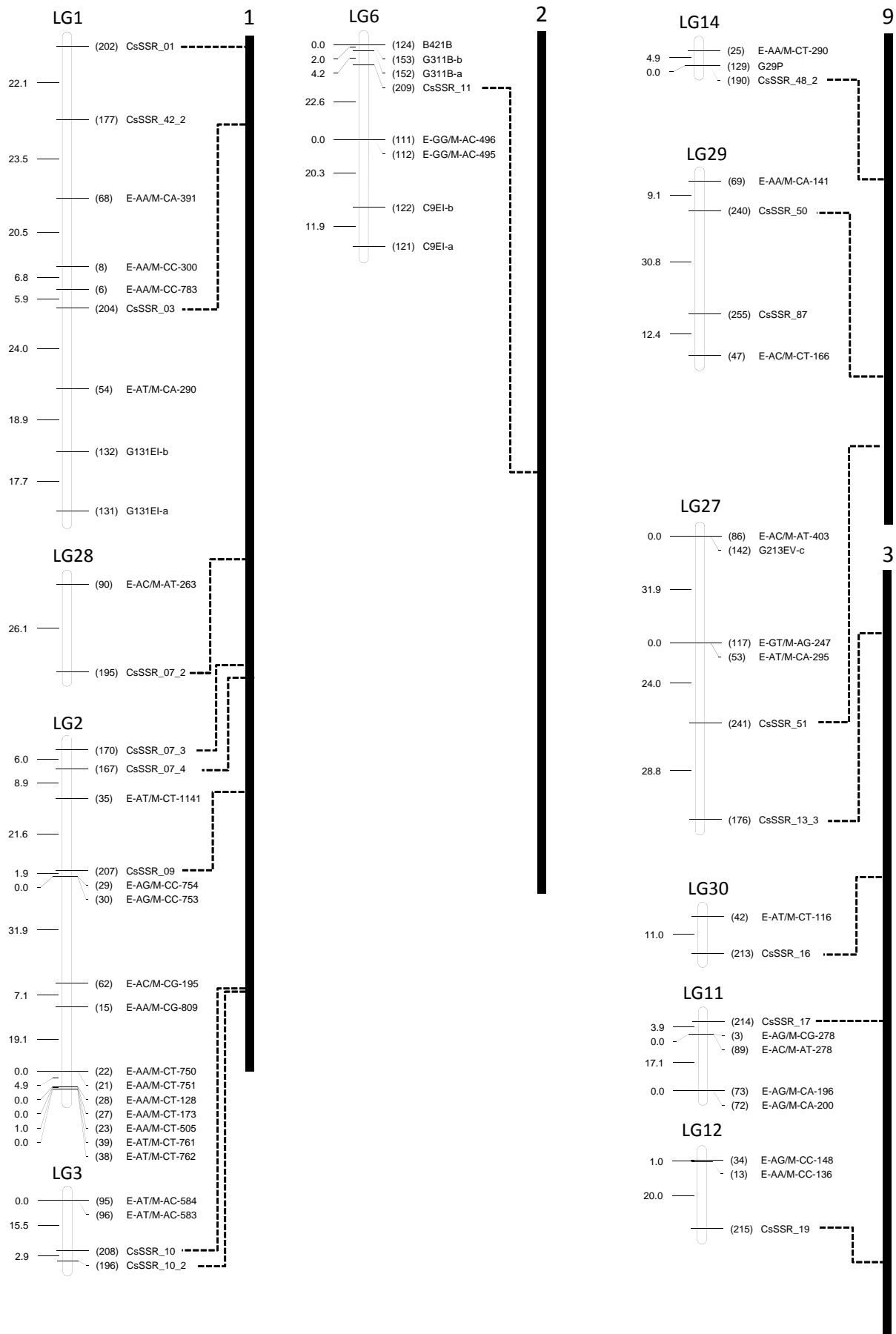

Figure S3

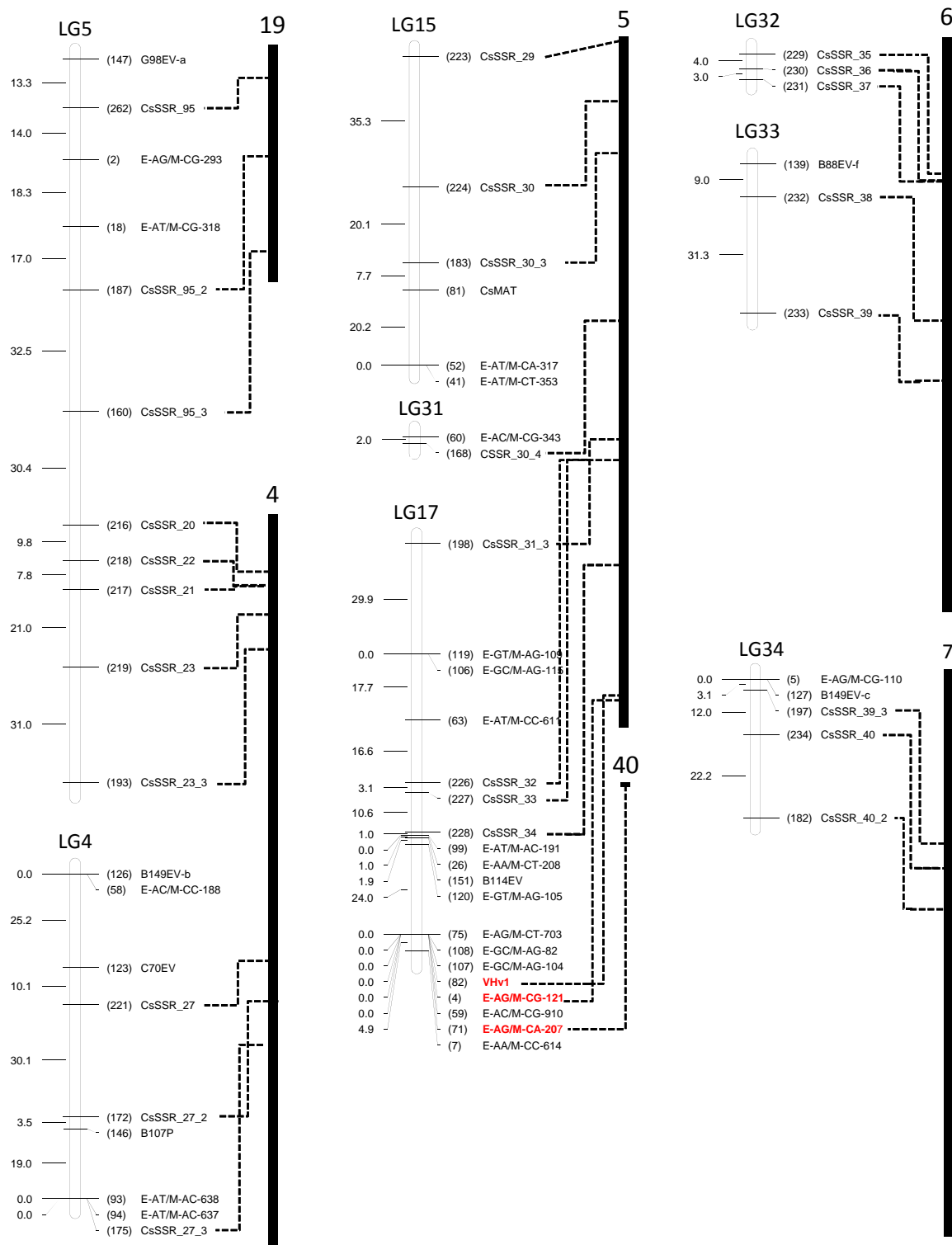

Figure S3, cont'd

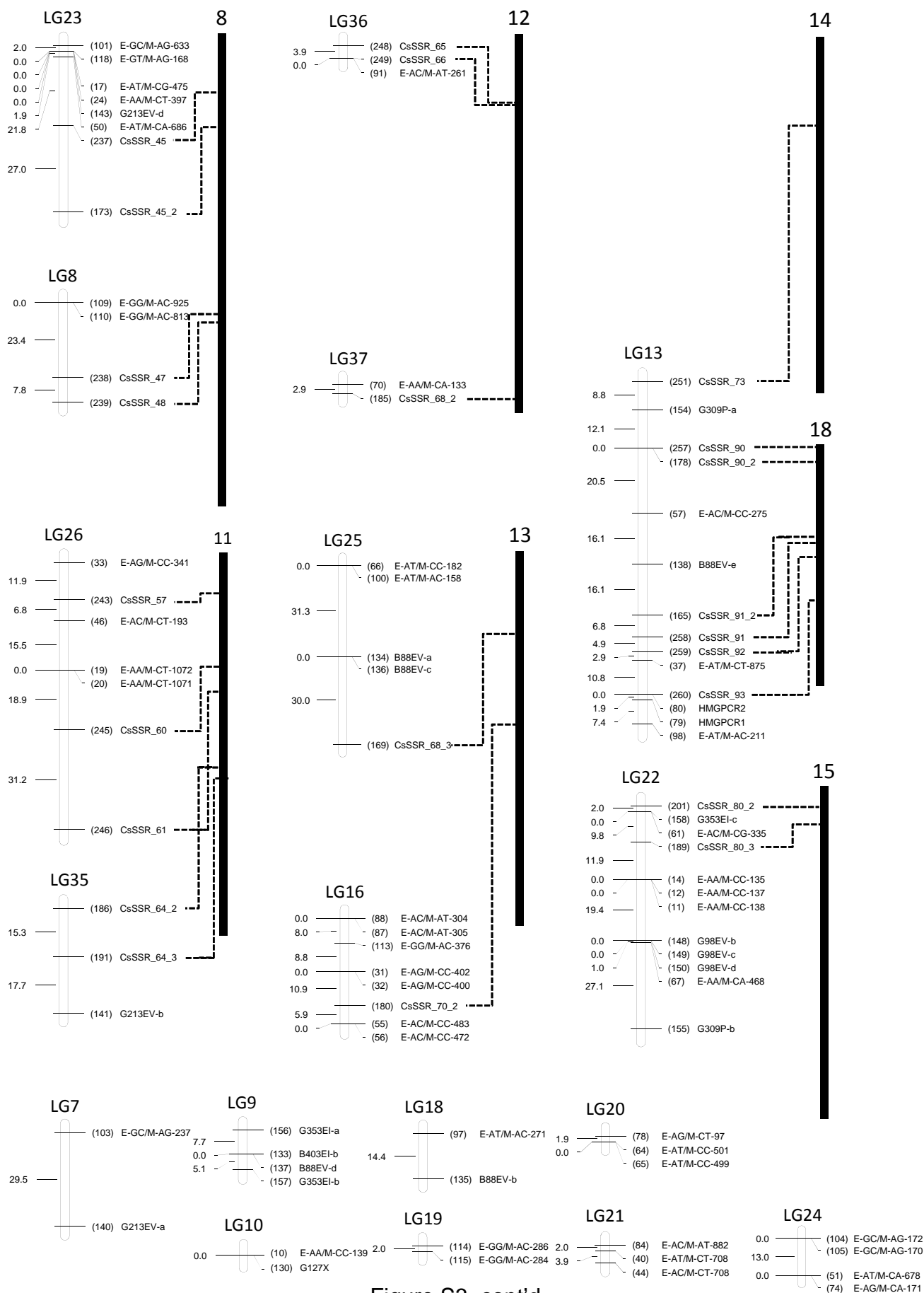

Figure S3, cont'd

Supplement: Figure S3 — C. sativus SSR sequences anchor sequenced scaffolds to the genetic map. Genetic map of C. sativus based on 68 SSR markers, 102 amplified fragment length polymorphism (AFLP) markers, 34 RFLP markers, two polymerase chain reaction–amplified markers, the mating type locus (CsMAT), and the barley cultivar-specific virulence locus (VHv1). Of the 37 linkage groups, 30 were assigned to 16 of the 157 scaffolds based on alignment of mapped SSR markers to the sequence assembly of ND90Pr. Linkage groups are on the left (open bars, numbered at the top) and assembled scaffolds (solid black bars, numbered at the top) are on the right. The start point of each scaffold is at the top. Dotted lines connect the SSR loci on the genetic map and physical map. AFLP markers flanking the VHv1 locus and the locus itself are highlighted in red (Figure 7). The scale bar corresponds to 500 kb. (PDF) [file pgen.1003233.s003.pdf]

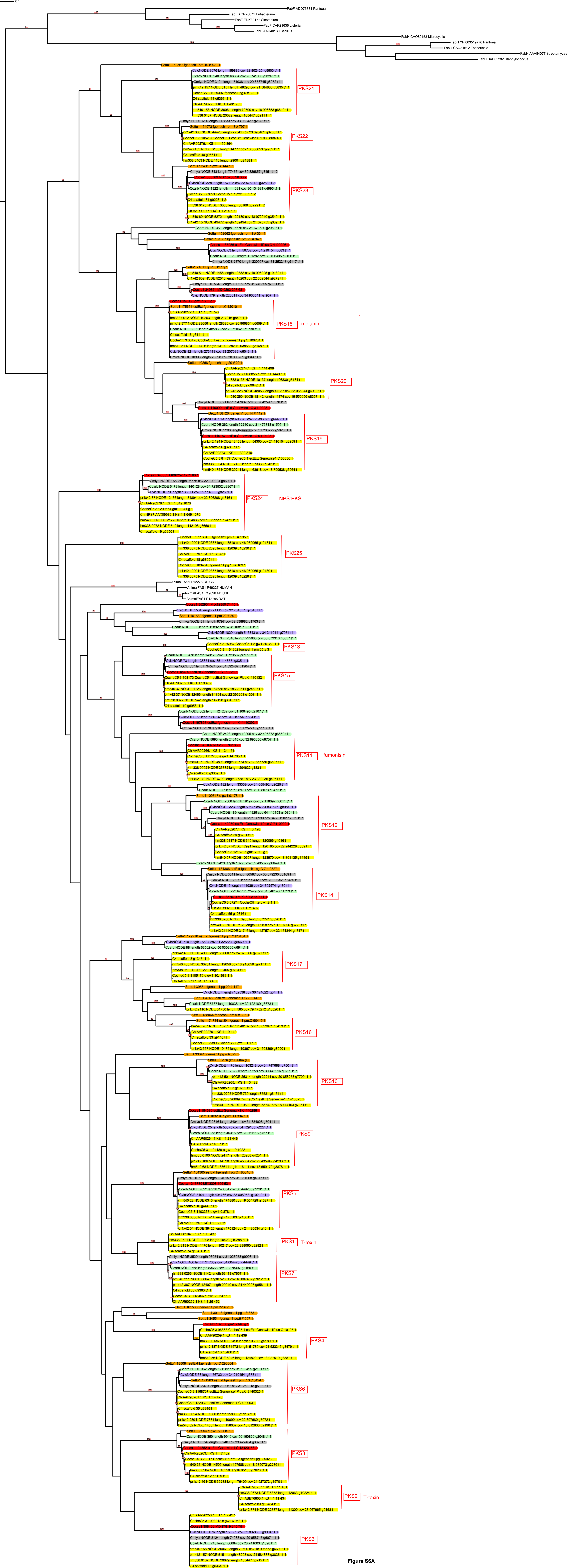

Figure S6A

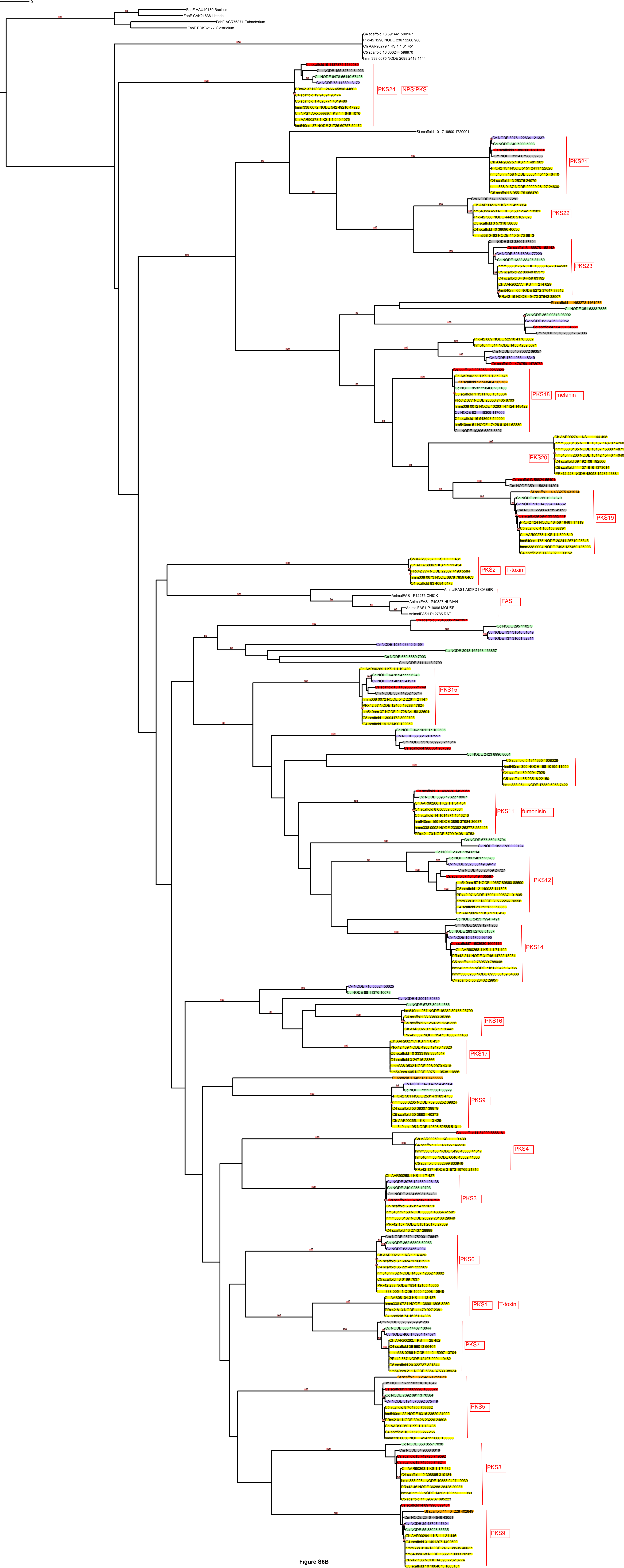

Figure S6B

Supplement: Figure S6 — Maximum likelihood tree of PKS ketosynthase (KS) domains identified using Augustus (A) and Genewise (B). RAxML using the WAGF model with a gamma distribution was used to infer the maximum likelihood tree and bootstrap support was determined using the fast-bootstrap method with 1000 bootstrap replicates. See Materials and Methods. Plain branches at the top of the tree are KS domains from related enzymes. KS domains are color-coded by species as in Figure S6. Bootstrap values above the branches. PKS11 is a C. heterostrophus ortholog of the Fusarium verticillioides PKS for fumonisin. (PDF) [file pgen.1003233.s006.pdf]

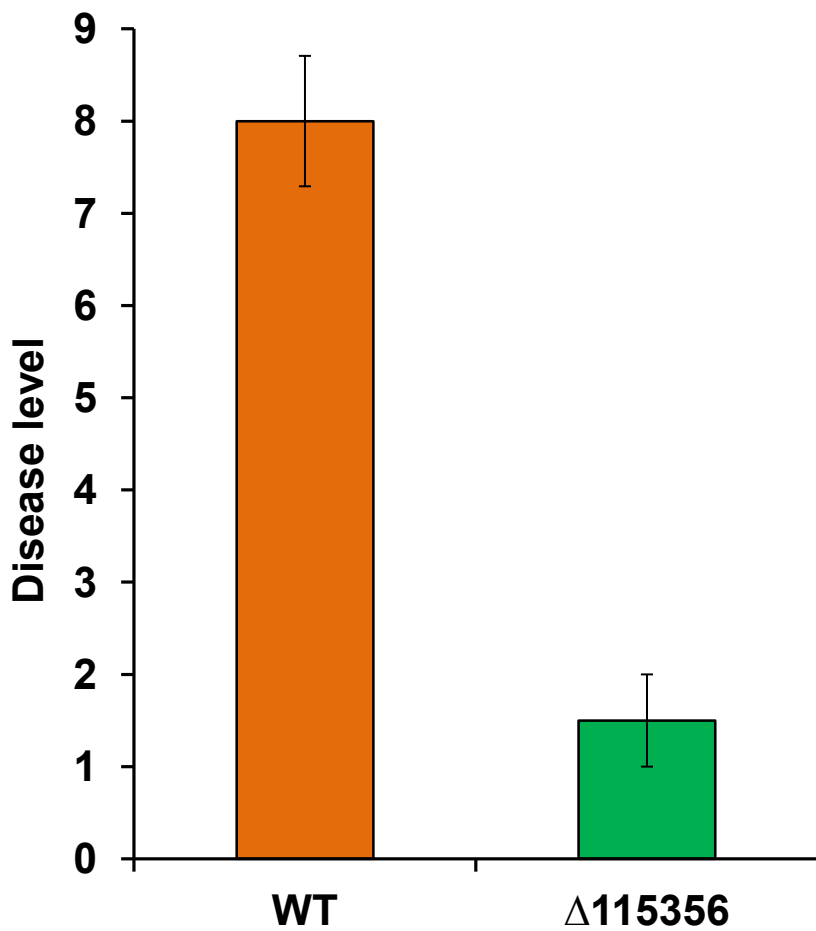

**Figure S7**

Supplement: Figure S7 — Quantification of spot blotch disease induced by the C. sativus wild type and mutant (Δ115356) on barley cv. Bowman. Disease rating was taken at 7 days after inoculation and is based on a 1 to 9 scale [118]. Four replicates were used. Error bar indicates the standard deviation. (PDF) [file pgen.1003233.s007.pdf]

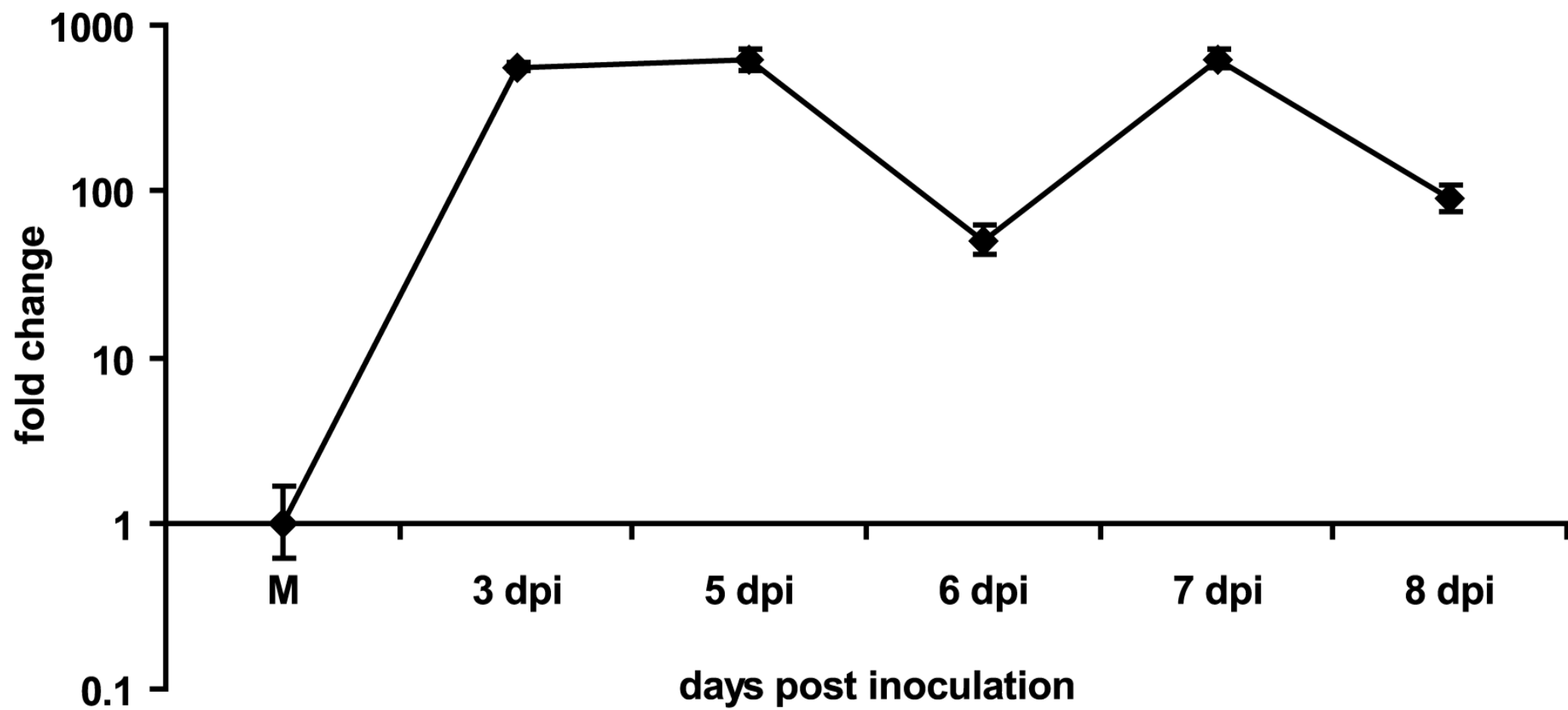

**Figure S8**

Supplement: Figure S8 — Quantitative real-time PCR analysis of S. turcica PKS gene (protein ID 161586) during infection of maize cultivar W64A-N. Gene expression was normalized based on the expression of the β-actin gene. Values are relative expression levels compared to that in mycelia grown on LCA medium. Samples were collected at 3, 5, 6, 7, and 8 days after inoculation. Primers are shown in Table S9. Error bars indicate the minimum and maximum relative expression values of the gene. (PDF) [file pgen.1003233.s008.pdf]
