# Supplementary material for: Comparative Genome Structure, Secondary Metabolite, and Effector Coding Capacity across Cochliobolus Pathogens
Source: PLoS Genet. 2013 Jan 24;9(1):e1003233. doi: 10.1371/journal.pgen.1003233 (PMC3554632; doi:10.1371/journal.pgen.1003233)
Supplement: Figure S4 — Analysis of the mating type region in Cochliobolus spp. and S. turcica. S. turcica 28A, C. sativus ND90Pr, C. carbonum 26-R-13, C. heterostrophus Hm540, and C. heterostrophus PR1x412, and the reference C. heterostrophus C5 strains are MAT1-1, while the others are MAT1-2. 10 kb regions flanking the MAT idiomorphs were aligned for each mating type. In all cases, the order of genes immediately surrounding the MAT locus (∼20 kb) was conserved, as described in [123] and [124]; genes flanking the MAT locus, differ from those flanking MAT in other ascomycetes [125]. JGI ID numbers are shown for strain C5 (MAT1-1) and C4 (MAT1-2). S. turcica had the most variation compared to the Cochliobolus MAT region, although the MAT genes were well conserved. Approximately 500 bp of the 5′ region and ∼3 kb of the 3′ region around the MAT gene were more variable than other regions when all genomes were compared. (PDF) [file pgen.1003233.s004.pdf]

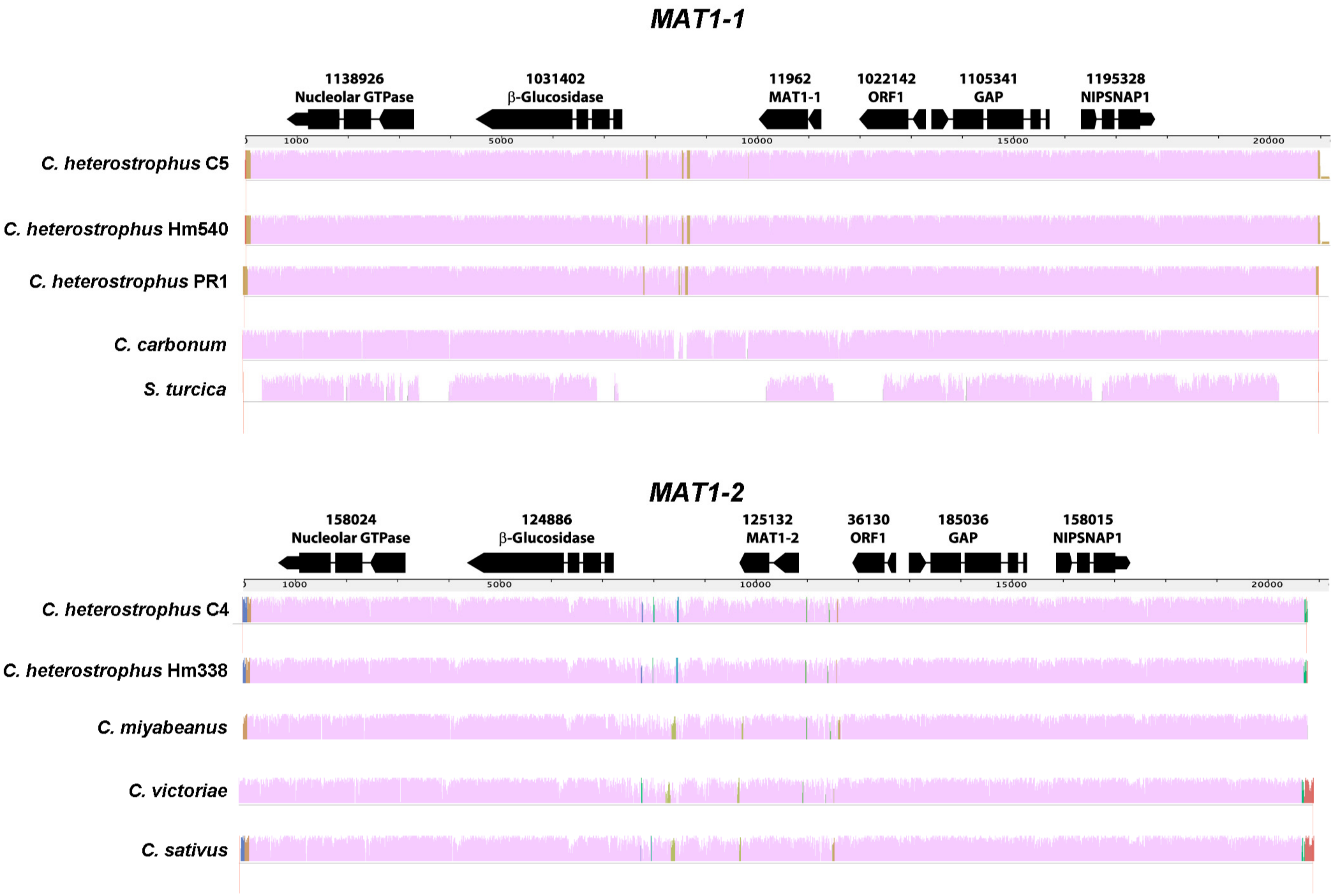
MAT1-1
1138926
Nucleolar GTPase
1031402
β-Glucosidase
11962
MAT1-1
1022142
ORF1
1105341
GAP
1195328
NIPSNAP1
1000
5000
10000
15000
20000
C. heterostrophus C5
C. heterostrophus Hm540
C. heterostrophus PR1
C. carbonum
S. turcica
MAT1-2
158024
Nucleolar GTPase
124886
β-Glucosidase
125132
MAT1-2
36130
ORF1
185036
GAP
158015
NIPSNAP1
1000
5000
10000
15000
20000
C. heterostrophus C4
C. heterostrophus Hm338
C. miyabeanus
C. victoriae
C. sativus

Figure S4
