# Supplementary material for: Comparative Genome Structure, Secondary Metabolite, and Effector Coding Capacity across Cochliobolus Pathogens
Source: PLoS Genet. 2013 Jan 24;9(1):e1003233. doi: 10.1371/journal.pgen.1003233 (PMC3554632; doi:10.1371/journal.pgen.1003233)
Supplement: Table S1 — Comparison of estimates for C. heterostrophus strain C5 chromosome sizes based on CHEF gel analysis and assembly size. (DOC) [file pgen.1003233.s009.doc]

| **Chromosome** | **CHEF Gel estimated size**  **(Mb) ^a^** | **Physical assembly size**  **(Mb) ^b^** |
| --- | --- | --- |
| 1 | 3.1 | 4.2 |
| 2 | 3.7 | 2.5 |
| 3 | 3.6 | 2.5 |
| 4 | 2.0 | 1.7 |
| 5 | 2.5 | 2.0 |
| 6 | 1.9 | 1.3 |
| 7 | 2.2 | 2.3 |
| 8 | 2.0 | 1.6 |
| 9 | 2.0 | 1.1 |
| 10 | 1.9 | 3.4 |
| 11 | 1.9 | 1.4 |
| 12 | 1.8 | 1.8 |
| 13 | 1.9 | 1.6 |
| 14 | 1.5 | 1.5 |
| 15 | 1.3 | 0.9 |
| 16 (B1) | 1.3 | 0.75 |
|  | 34.6 | 30.55 |

**Table S1.** Comparison of estimates for *C. heterostrophus* strain C5

chromosome sizes based on CHEF gel analysis and assembly size.

**^a^** Based on Tzeng et al 1992

^b^ Based on JGI assembly
