# Supplementary material for: Comparative Genome Structure, Secondary Metabolite, and Effector Coding Capacity across Cochliobolus Pathogens
Source: PLoS Genet. 2013 Jan 24;9(1):e1003233. doi: 10.1371/journal.pgen.1003233 (PMC3554632; doi:10.1371/journal.pgen.1003233)
Supplement: Table S4 — MUMMER MAT alignments. (DOC) [file pgen.1003233.s012.doc]

| **Table S4.** MUMMER alignment of *MAT* loci | | | |  | |  | |  |
| --- | --- | --- | --- | --- | --- | --- | --- | --- |
| **Reference** | **Query** | **# SNPs between loci** | **Mating type comparison** | | **predicted # SNPS** | |  | |
| *Ch* C5 | *Ch* C4 | 91 | opposite | | 1 | |  | |
| *Ch* C5 | *Ch* Hm338 | 220 | opposite | | 19 | |  | |
| *Ch* C5 | *Ch* Hm540 | 15 | same | | 33 | |  | |
| *Ch* C5 | *Ch* PR1 | 0 | same | | 22 | |  | |
|  |  |  |  | |  | |  | |
| *Ch* C5 | *C. carb* | 1622 | same | | 1340 | |  | |
| *Ch* C5 | *C. vict* | 1532 | opposite | | 1300 | |  | |
| *Ch* C5 | *C. miya* | 1712 | opposite | | 1348 | |  | |
| *Ch* C5 | *C. sat* | 1446 | opposite | | 1209 | |  | |
| *Ch* C5 | *S. tur* | 3458 | same | | 2269 | |  | |
|  |  |  |  |  | |  | |  |
| *Ch* C4 | *Ch* C5 | 91 | opposite |  | |  | |  |
| *Ch* C4 | *Ch* Hm338 | 12 | same |  | |  | |  |
| *Ch* C4 | *Ch* Hm540 | 94 | opposite |  | |  | |  |
| *Ch* C4 | *Ch* PR1 | 91 | opposite |  | |  | |  |
| *Ch* C4 | *C. carb* | 1548 | opposite |  | |  | |  |
| *Ch* C4 | *C. vict* | 1644 | same |  | |  | |  |
| *Ch* C4 | *C. miya* | 1823 | same |  | |  | |  |
| *Ch* C4 | *C. sat* | 1554 | same |  | |  | |  |
| *Ch* C4 | *S. tur* | 3239 | opposite |  | |  | |  |
|  |  |  |  |  | |  | |  |
| *C. carb* | *C. vict* | 121 | opposite |  | |  | |  |
| *C. carb* | *C. sat* | 1539 | opposite |  | |  | |  |
| *C. vict* | *C. miya* | 1775 | same |  | |  | |  |

*C. heterostrophus* strains C5, Hm540, and PR1x412, *C. carbonum* strain 26-R-13*, S. turcica* strains 28A are *MAT1-1*, while *C. heterostrophus* strains C4 and Hm338, *C. victoriae* strain FI3*, C. miyabeanus* strain WK1C*,* and *C. sativus*  strain ND90R are *MAT1-2*.  Opposite indicates opposite mating type, same indicates same mating type.
