# Supplementary material for: Comparative Genome Structure, Secondary Metabolite, and Effector Coding Capacity across Cochliobolus Pathogens
Source: PLoS Genet. 2013 Jan 24;9(1):e1003233. doi: 10.1371/journal.pgen.1003233 (PMC3554632; doi:10.1371/journal.pgen.1003233)
Supplement: Table S5 — Regions of low coverage of C5, by other sequences. (DOC) [file pgen.1003233.s013.doc]

| **Table S5.** Regions of low coverage to the *C. heterostrophus* strain C5 reference strain. | | | | | | | | | | | | |
| --- | --- | --- | --- | --- | --- | --- | --- | --- | --- | --- | --- | --- |
| **Comparison** | **Inbred unique^a^** | | | | **Race O unique^b^** | | | | ***C. heterostrophus* core^c^** | | | |
| Low coverage included | Hm338, Hm540, PR1x412 | | | | C4, HM338, PR1x412 | | | | *Cc, Cv, Cm, Cs* | | | |
| Low coverage excluded | C4 | | | | HM540 | | | | C4, Hm540, Hm338, PR1x412 | | | |
| Cutoff | **0** | **100bp** | **5kb** | **0** | | **100bp** | **5kb** | **0** | | **100bp** | **5kb** |  |
| # Regions | 291 | 64 | 0 | 11 | | 3 | 0 | 24,754 | | 13,539 | 187 |  |
| Sum | 28,556 | 24,366 | 0 | 4,309 | | 4,281 | 0 | 11,756,765 | | 11,399,570 | 1,606,829 |  |
| Average | 98 | 381 | 0 | 392 | | 1,427 | 0 | 475 | | 842 | 8,593 |  |

**^a^**sequence ONLY in inbred C strains (C5, C4) compared to field strains

**^b^**sequence ONLY in race O strains

**^c^**Sequence ONLY in *C. heterostrophus*, and found in all *C. heterostrophus* strains
