# Supplementary material for: Comparative Genome Structure, Secondary Metabolite, and Effector Coding Capacity across Cochliobolus Pathogens
Source: PLoS Genet. 2013 Jan 24;9(1):e1003233. doi: 10.1371/journal.pgen.1003233 (PMC3554632; doi:10.1371/journal.pgen.1003233)
Supplement: Table S7 — Forty three predicted genes in the unique region of isolate ND90Pr associated with the virulence locus VHv1. (DOC) [file pgen.1003233.s015.doc]

**Table S7**. Summary of 43 predicted genes in the unique region of isolate ND90Pr associated with the virulence locus *VHv1*.

| **Protein ID ^a^** | **Location** | **KOG or GO Description** | **Best hit ^b^** | **Accession number** | **Organism** | **Percent identity** |
| --- | --- | --- | --- | --- | --- | --- |
|  |  |  |  |  |  |  |
| **140513** | 2045891-2057961 | Non-ribosomal peptide synthetase | HC-toxin synthetase | XP_001940764.1 | *P. tritici-repentis* | 24 |
| 86773 | 2059008-2059988 | 0003676:nucleic acid binding;0004523:ribonuclease H activity | HP | XP_001587081.1 | *S. sclerotiorum* | 35 |
| 61253 | 2062497-2063609 | DNA-binding centromere protein B (CENP-B) | HP | XP_003297107.1 | *P. teres f. teres* | 39 |
| 46085 | 2063643-2063858 |  |  |  |  |  |
| 35945 | 2065409-2070353 | Multidrug/pheromone exporter, ABC superfamily | pmd1 | XP_001940759.1 | *P. tritici-repentis* | 81 |
| 35946 | 2071052-2072167 | 3-hydroxyacyl-CoA dehydrogenase | HP | XP_001793390.1 | *P. nodorum* | 59 |
| **115356** | 2073740-2077584 | Non-ribosomal peptide synthetase | HP | XP_003305983 | *P. teres f. teres* | 36 |
| 35949 | 2082262-2083366 | Dehydrogenases with different specificities (related to short-chain alcohol dehydrogenases) | short chain dehydrogenase family protein | XP_002480320.1 | *T. stipitatus* | 40 |
| 86818 | 2083881-2084063 |  |  |  |  |  |
| 51865 | 2085983-2086171 |  |  |  |  |  |
| 140533 | 2087647-2089329 |  |  |  |  |  |
| 35954 | 2089462-2091622 |  |  |  |  |  |
| 49666 | 2093485-2093673 |  |  |  |  |  |
| 35956 | 2094441-2095038 |  |  |  |  |  |
| 86617 | 2095152-2096828 |  |  |  |  |  |
| 86221 | 2097749-2097916 | 0003677: DNA binding | HP | XP_003297743.1 | *P. teres f. teres* | 64 |
| **Table S6**. Continued. | | | | | | |
| **Protein ID ^a^** | **Location** | **KOG or GO Description** | **Best hit ^b^** | **Accession number** | **Organism** | **Percent identity** |
| 311945 | 2098674-2099345 |  |  |  |  |  |
| 86794 | 2099539-2101179 | DNA-binding centromere protein B (CENP-B) | HP | XP_003297743.1 | *P. teres f. teres* | 67 |
| 311967 | 2102242-2102971 |  |  |  |  |  |
| 170432 | 2104616-2106963 |  |  |  |  |  |
| 35965 | 2108595-2109682 |  |  |  |  |  |
| 159600 | 2111623-2111996 |  |  |  |  |  |
| 170433 | 2113530-2114021 |  |  |  |  |  |
| 87064 | 2114516-2115957 | Mitogen-activated protein kinase kinase (MAP2K) | Serine/threonine protein kinase | XP_001934630.1 | *P. tritici-repentis* | 72 |
| 170435 | 2118188-2118622 |  |  |  |  |  |
| 312157 | 2119914-2121265 | 0005515:protein binding | HP | XP_001940056.1 | *P. tritici-repentis* | 33 |
| 170437 | 2122464-2124601 | Vacuolar sorting protein VPS1, dynamin, and related proteins | interferon-induced GTP-binding protein | XP_001942221.1 | *P. tritici-repentis* | 83 |
| 35975 | 2126976-2128512 | 0005515:protein binding | HP | XP_001793198.1 | *P. nodorum* | 39 |
| 25598 | 2131334-2131852 |  |  |  |  |  |
| 159607 | 2133172-2134386 |  |  |  |  |  |
| 86437 | 2135136-2135816 | 0005515:protein binding | HP | XP_002623588.1 | *A. dermatitidis* | 46 |
| **Table S6. Continued.** | | | | | | |
| **Protein ID ^a^** | **Location** | **KOG or GO Description** | **Best hit ^b^** | **Accession number** | **Organism** | **Percent identity** |
| 198855 | 2137909-2139145 | FOG: Predicted E3 ubiquitin ligase | predicted protein | XP_002583523.1 | *U. reesii* | 34 |
| 189677 | 2139431-2140630 | SAM-dependent methyltransferases | HP | XP_003295285.1 | *P. teres f. teres* | 87 |
| 355571 | 2141717-2142144 |  |  |  |  |  |
| 159610 | 2142607-2143125 |  |  |  |  |  |
| 35979 | 2164154-2164524 |  |  |  |  |  |
| 86951 | 2167779-2170096 | Predicted histone tail methylase containing SET domain | HP | XP_003297709.1 | *P. teres f. teres* | 62 |
| 115406 | 2171986-2172249 |  |  |  |  |  |
| 70978 | 2172433-2172585 |  |  |  |  |  |
| 86652 | 2172874-2173784 |  |  |  |  |  |
| 35982 | 2174435-2175346 |  |  |  |  |  |
| 355596 | 2175549-2176935 |  |  |  |  |  |
| 355600 | 2177116-2177878 |  |  |  |  |  |
| ^a^ JGI protein ID (http://genome.jgi-psf.org/Cocsa1/Cocsa1.home.html).  ^b^ The best hit gene name. HP indicates hypothetical protein. | | | | | | |
