# Supplementary material for: Comparative Genome Structure, Secondary Metabolite, and Effector Coding Capacity across Cochliobolus Pathogens
Source: PLoS Genet. 2013 Jan 24;9(1):e1003233. doi: 10.1371/journal.pgen.1003233 (PMC3554632; doi:10.1371/journal.pgen.1003233)
Supplement: Table S8 — Summary of repetitive elements identified in the unique region of isolate ND90Pr. (DOC) [file pgen.1003233.s016.doc]

**Table S8.** Summary of repetitive elements identified in the unique region of isolate ND90Pr.

| **Name** | | **Description** | **Length (bp)** | **Position** |
| --- | --- | --- | --- | --- |
| Mariner-9_AN | | DNA transposon | 508 | 2062895-2063402 |
| MOLLY_SN | | DNA transposon | 258 | 2081114-2081371 |
| Mariner2_AO | | DNA transposon | 88 | 2081551-2081638 |
| I-1_AO | | Non-LTR retrotransposons | 1004 | 2085411-2086414 |
| I-3_AO | | Non-LTR retrotransposons | 595 | 2093079-2093673 |
| I-1_AO | | Non-LTR retrotransposons | 753 | 2093140-2093892 |
| Mariner-3_AF | | DNA transposon | 821 | 2097833-2098653 |
| MOLLY_SN | | DNA transposon | 826 | 2098059-2098884 |
| Mariner-3_AF | | DNA transposon | 854 | 2099680-2100533 |
| MOLLY_SN | | DNA transposon | 646 | 2099907-2100552 |
| MOLLY_SN | | DNA transposon | 535 | 2100018-2100552 |
| Mariner-1_AF | | DNA transposon | 704 | 2100038-2100741 |
| I-5_AO | | Non-LTR retrotransposons | 331 | 2102536-2102866 |
| I-5_AO | | Non-LTR retrotransposons | 2476 | 2107575-2110050 |
| Mariner-3_AF | | DNA transposon | 821 | 2129688-2130508 |
| MOLLY_SN | | DNA transposon | 642 | 2129919-2130560 |
|  | Mariner-1_AF | DNA transposon | 658 | 2130046-2130703 |
|  | I-5_AO | Non-LTR retrotransposons | 162 | 2140886-2141047 |
