# Supplementary material for: Comparative Genome Structure, Secondary Metabolite, and Effector Coding Capacity across Cochliobolus Pathogens
Source: PLoS Genet. 2013 Jan 24;9(1):e1003233. doi: 10.1371/journal.pgen.1003233 (PMC3554632; doi:10.1371/journal.pgen.1003233)
Supplement: Table S9 — Primers used in quantitative real time-PCR of C. sativus and S. turcica genes. (DOC) [file pgen.1003233.s017.doc]

**Table S9.** Primers used in the quantitative real time-PCR.

| **Primer name^a^** | **Primer sequence (5'-3')** |
| --- | --- |
| RT-Actin-F | GTATGGGCCAAAAGGACTCA |
| RT-Actin-R | CACGCAGCTCGTTGTAGAAG |
| 350779-F1 | GCATGGGAATCGACAGAACT |
| 350779-R1 | CGTACATGCTCTTCGGTGAA |
| 130053-F1 | GAGCCGCTGTGCTCTATACC |
| 130053-R1 | CGGAACTTCTGGGAAAACTG |
| 49884-F1 | CAGGTGAAGCTACACGGACA |
| 49884-R1 | CTCAAGACGAACGGGAGGTA |
| 151356-F1 | GTCGACTGCCATCTGGAAAC |
| 151356-R1 | TGCGGGGTCATCTTAAAAAC |
| 140513-F1 | CAAATACACGCCAGAATCCA |
| 140513-R1 | CCTGGTCCGCAGAAAGATAG |
| DW489^*^ | TGCAATCTCTCACCATCGTC |
| DW490^*^ | GGTCGCTGATTACTGCCTTC |

^a^All primers used with *C. sativus* except DW489 = *S. turcica* 161586 qPCR forward primer, and DW490 = *S. turcica* 161586 qPCR reverse primer.
