# Supplementary material for: Comparative Genome Structure, Secondary Metabolite, and Effector Coding Capacity across Cochliobolus Pathogens
Source: PLoS Genet. 2013 Jan 24;9(1):e1003233. doi: 10.1371/journal.pgen.1003233 (PMC3554632; doi:10.1371/journal.pgen.1003233)
Supplement: Text S1 — Supplementary Methods. Mapping scaffolds onto the C. heterostrophus genetic map. Mapping scaffolds to the C. sativus genetic map. Mating type region comparisons. (DOCX) [file pgen.1003233.s020.docx]

**Supplementary Methods**

**Mapping scaffolds onto the *C. heterostrophus* genetic map.**

An RFLP map with 125 markers was constructed previously using *C. heterostrophus* race O field strain Hm540 and race T C-strain B30.A3.R.45 as parents [1]. When RFLP marker sequences were used as blast queries against the strain C5 genome assembly, 62 markers could be placed unambiguously. Placed markers were used to unite scaffolds and genetic linkage groups (Figure 1, Tables S1, 2). Twenty-nine scaffolds (denoted by internal JGI IDs, Table S2) were combined to create 16 genetically grounded scaffolds (30.6 Mb total), 14 of which could be oriented by the presence of multiple markers; 4.94 Mb of sequence on 28 scaffolds was left unplaced (Figure 1). In addition, 24 unplaced fosmids that could not be placed in the assembly, but which contained valid sequence, were added as individual scaffolds (864 kb).

For markers that mapped to the same scaffold, genetic distance could be directly compared to physical distance. Twenty nine such pairwise comparisons were made, showing an average ratio of 13 kb/cM (ranging from 4.2-30 kb/cM). For a number of the linkage groups, the sum physical distance is very near previous estimates for chromosome size: assemblies for linkage groups 4, 7, 12, 13 and 14 are all within 300 kb of predictions based on Clamped Homogeneous Electric Field (CHEF) gels [1,2] (Table S1). Other assembled linkage groups have much more physical sequence than previously estimated. Chromosome 10, for example, estimated by CHEF gels to be 1.9 Mb, appears to be 3.4 Mb when assembled, with the caveat that each scaffold is linked by a single marker only (the *MAT* locus and RFLP B285) (Figure 1, Table S1). Chromosome 1, estimated to be 3.1 Mb but assembling to 4.2 Mb, is a more robust example, as each scaffold is linked by at least two RFLPs (Figure 1, Table S1). There is a gross difference in physical size and genetic distance between markers B277 and B91 on this chromosome (Figure 1), however, suggesting that recombination is very rare between these two markers, or that a structural rearrangement has occurred on this chromosome in the sequenced C5 strain. Chromosomes 2 and 3, on the other hand, assemble to only 2.5 Mb, while CHEF methods estimate 3.7 and 3.6 Mb, respectively. For total C5 genome size, however, previous estimates (34.6 Mb, [2]) are very near that of the final assembly (36.46 Mb, Table S1, Figure 1). Note that the total assembled genome size of isogenic *C. heterostrophus* strain C4 is 32.93 Mb. This is less than predicted since we know that a unique region (not in strain C5) encoding the *Tox1* locus is ~1.2 Mb. Therefore, based on the C5 genome size, we estimate the C4 genome size should be ~ 36.46 + 1.2 = 37.66 Mb. Kodama et al [2] predict ~ 35.8 Mb. This discrepancy is likely due to the fact that C4 was sequenced using Illumina, an approach that results in underestimations of repeat content coupled with the fact that the 1.2 Mb *Tox1* region contains an abundance of difficult to assemble repeats [3,4].

**Breakpoint linkage group.** Strains C5 and C4 are progeny of a backcross series selected on the basis of whether or not they carried the *Tox1* locus responsible for T-toxin production [5]. As noted above, estimates from chromosome separation gels suggest that there is at least 1.2 Mb of DNA in race T strain C4 that is not present in race O strain C5 [1,2,3]. It has been shown previously that the *Tox1* locus maps to the intersection of a four armed linkage group consisting of two race O chromosomes (chromosomes 6 and 12) and two race T chromosomes (chromosomes 6;12 and 12;6) that have undergone a reciprocal translocation with respect to the race O pair [1,2]. Mapping of scaffolds to the genetic map identified scaffolds 11027.2 and 11057 mapping to race O linkage group 6 (chromosome 6) and scaffolds 11053 and 11035 mapping to C5 linkage group 12 (chromosome 12), which were combined to form scaffolds 6 and 12 in the final assembly (Figure 1).

Chromosome 6 (1.3 Mb) of C5 is well covered by isogenic race T C4 scaffolds 13 (900 kb) and 33 (270 kb). Although both of these scaffolds aligned to a single chromosome (6) of C5, they are likely split onto the two reciprocally translocated chromosomes, 6:12 and 12:6, of C4. C5 chromosome 12 (1.8 Mb) is primarily covered by C4 scaffold 20 (56 kb), scaffold 29, (161 kb), scaffold 47 (146 kb), scaffold 55 (80 kb), and scaffold 11 (1 Mb). On both chromosome 6 and 12, there is a region between aligned C4 scaffolds where there is poor coverage (Figure 3). To date, no scaffold has been identified that clearly spans the breakpoints of the reciprocal translocations, likely due to the repetitive nature of *Tox1*-associated DNA. This leaves the exact physical positions of the 1.2 Mb of *Tox1* DNA unresolved.

**Dispensable chromosome**. Many markers mapped to linkage group B1 (scaffold 16, JGI ID 11041) which corresponds to the dispensable chromosome on the original map. This chromosome is present in the parent race T strain B30.A3.R.45 [1] used to build the RFLP map and is also in reference race O strain C5. It was not, however, present in the second parent (Hm540) of the original genetic map. Because of this, there are no frequencies of crossing over to calculate genetic distance for this chromosome.

When *C. heterostrophus* Hm540 was aligned to the C5 reference genome, only small, non-co-linear islands of the B1 chromosome could be mapped with Hm540 sequences, although Hm540 sequences aligned well to other C5 chromosomes/scaffolds (Figure S2). This gap was specific to *C. heterostrophus* Hm540, as strain C4, Hm338 and PR1x412 sequence reads could be mapped to this region. This supports the argument that chromosome B1 is a dispensable chromosome and that it is missing from strain Hm540 (Figure S2). This chromosome did not meet dispensable chromosome criteria set in Ohm *et al.* (PLoS Pathogens, submitted), as the gene density (392.9 genes/Mb) is too high and repeat content (4.89%) too low. No known virulence factors map to the *C. heterostrophus* B chromosome. Thus, while the genetic evidence for chromosome B1 being dispensable is strong, it does not fit the pattern of several other dispensable chromosomes. There were no RFLP markers linking this scaffold to our map, and the JGI synteny browser allowed us to assess the presence and co-linearity of Scaffold 16 (chromosome B1) in other strains and species. Scaffold 16 (chromosome B1) is present, in its entirety, in isogenic strain C4 (scaffold 18), along with small regions (<1 kb) distributed on other scaffolds. When aligned to *C. sativus*, however, there is almost no co-linearity, although the majority of the scaffold has alignments, generally < 20 kb long (not shown). This pattern of reduced co-linearity of this chromosome/scaffold, compared to conservation for the rest of the genome is also evident when comparisons are made to other species examined here.

**Telomeres**. Twelve candidate telomeric sequences were identified in the JGI C5 assembly out of the complete inventory of 32 when scaffolds were searched for repeats with telomeric sequence CCCTAA. Six were placed on mapped scaffolds, with only a single linkage group (14, scaffolds 11055 and 11038) having both telomeres. Six additional telomere calls were on unplaced scaffolds (S18, 23, 27, 50, 52, 57), however, there are no data linking these scaffolds to any particular chromosome.

**Mapping scaffolds to the *C. sativus* genetic map**

To add more markers to the previously constructed *C. sativus* genetic map [6] and associate scaffolds with linkage groups, 121 polymorphic simple sequence repeat (SSR) markers were identified in the assembly sequences of the ND90Pr and ND93-1 parents. Of these, 106 segregated in a 1:1 ratio, while 15 exhibited distorted segregation, ten at the 5% and five at the 1%, significance level. A final genetic linkage map (Figure S3) was constructed with 68 SSR markers and 140 previously mapped markers, including 102 amplified fragment length polymorphism (AFLP), 34 RFLP and two PCR markers, plus the mating type (*MAT*) and the *VHv1* locus associated with virulence of pathotype 2 isolate ND90Pr on barley cv Bowman [6]. The markers (208 in total) were associated with 37 linkage groups which had at least two markers, when a minimum logarithm of odds (LOD) value of 4.0 and a maximum theta of 0.3 were used in the MAPMAKER program [7]. Since 30 of these linkage groups contained SSR markers, they could be associated with 16 scaffolds, summing to 29.32 Mb. Seven linkage groups were unassigned (Figure S3). When DNA sequences of the two AFLP markers (E-AG/M-CA-207 and E-AG/M-CG-121, Figure S3), co-segregating with the virulence locus *VHv1,* were used as blast queries against the ND90Pr genome assembly, E-AG/M-CG-121 mapped to coordinates 2,132,630-2,132,734 of scaffold 5, while E-AG/M-CA-207 mapped to a thioesterase gene (protein ID 42084), unique to ND90Pr on scaffold 40, on a small contig (6,399 bp) that is likely linked to scaffold 5 because it carries the second AFLP marker that co-segregates with the *VHv1* locus (Figure S3)*.*

Comparison of the physical and genetic distances between 32 pairs of SSR markers that mapped to the same scaffold indicated that the ratios varied and ranged from 0.67 to 13.28 kb/cM with an average of 5.07 kb/cM).

**Mating type region comparisons**

*Cochliobolus*, like other heterothallic Dothideomycetes, has two mating type idiomorphs [8,9], *MAT1-1* and *MAT1-2*, and these were identified in all genomes. The *C. heterostrophus* reference C5, Hm540, and PR1x412, *C. carbonum* 26-R-13 and *S. turcica* strains were *MAT1-1*, while the others were *MAT1-2*. 10 kb regions flanking the *MAT* idiomorphs were aligned for each mating type. In all cases, the order of genes immediately surrounding the *MAT* locus was conserved (Figure S4). Thus the larger collection of isolates confirms earlier data regarding which genes are encoded on the *MAT* flanks and that these are indeed different from genes flanking *MAT* loci in other ascomycetes [8]. *S. turcica*, not surprisingly, had the most variation in these regions compared to the *Cochliobolus* regions, however the same genes were present (Figure S4). Although the *MAT* and flanking genes themselves were well conserved compared to those in the other species, 500 bp of the 5’ region and ~3 kb of the 3’ region (Figure S4) were highly variable compared to these regions in *Cochliobolus*. There were other, smaller, gaps elsewhere in the 20 kb region analyzed.

The number of SNPs called when aligning *MAT* regions (excluding the *MAT* genes themselves when comparing *MAT1-1* to *MAT1-2*) was lowest when comparing within *C. heterostrophus* species (Table S4). When *C. heterostrophus* strains of the same mating type were compared, there were only 0 [C5 (*MAT1-1*) and PR1x412 (*MAT1-1*)], 15, [C5 (*MAT1-1*) and Hm540 (*MAT1-1*)] or 12 [C4 (*MAT1-2*) and Hm338 (*MAT1-2*)] SNPs called. Within the species, (i.e., comparing *C. heterostrophus* *MAT1-1* flanks to *MAT1-2* flanks), however, there were 100-200 SNP calls in each alignment. *C. heterostrophus* C4 and C5 are the most similar strains on the genome scale (Table 3), and the average number of bps/SNP would predict only a single SNP across the 20 kb flanking regions outside the *MAT* genes. Instead, there were 91 between C4 and C5, on par with the other *MAT1-1* flank to *MAT1-2* flank comparisons across mating type within the species. For all other *C. heterostrophus* strains and *Cochliobolus* species aligned to C5, the number of SNPs called was slightly higher than the number predicted by the whole-genome average bps/SNP. Aligning the 20 kb of *MAT* flanking DNA across species produced 1500-1823 SNPs, regardless of whether or not the comparison was for regions carrying the same or different *MAT* genes, and over twice as many SNPs when aligned to *S. turcica*, when either C4 (*MAT1*-*2*) or C5 (*MAT1*-*1*) was used. *C. carbonum* (*MAT1-1*) and *C. victoriae* (*MAT1-2*) had only 121 SNPs when aligned to each other, fewer than comparing *C. heterostrophus* C5 (*MAT1-1*) to *C. heterostrophus* Hm338 (*MAT1-2*). The observation that the similarity between *MAT* regions of *C. carbonum* and *C. victoriae* is comparable to that of *MAT* loci within *C. heterostrophus* strains is consistent with their close phylogenetic relationship, and, ability to cross to one another.

**References**

1. Tzeng TH, Lyngholm LK, Ford CF, Bronson CR (1992) A restriction fragment length polymorphism map and electrophoretic karyotype of the fungal maize pathogen *Cochliobolus heterostrophus*. Genetics 130: 81-96.

2. Kodama M, Rose MS, Yang G, Yun SH, Yoder OC, et al. (1999) The translocation-associated *Tox1* locus of *Cochliobolus heterostrophus* is two genetic elements on two different chromosomes. Genetics 151: 585-596.

3. Turgeon BG, Baker SE (2007) Genetic and genomic dissection of the *Cochliobolus heterostrophus Tox1* locus controlling biosynthesis of the polyketide virulence factor T-toxin. Adv Genet 57: 219-261.

4. Inderbitzin P, Asvarak T, Turgeon BG (2010) Six new genes required for production of T-toxin, a polyketide determinant of high virulence of *Cochliobolus heterostrophus* to maize. Mol Plant Microbe Interact 23: 458-472.

5. Leach J, Lang BR, Yoder OC (1982) Methods for selection of mutants and *in vitro* culture of *Cochliobolus heterostrophus*. J Gen Microbiol 128: 1719-1729.

6. Zhong S, Steffenson BJ, Martinez JP, Ciuffetti LM (2002) A molecular genetic map and electrophoretic karyotype of the plant pathogenic fungus *Cochliobolus sativus*. MPMI 15: 481-492.

7. Lander ES, Green P, Abrahamson J, Barlow A, Daly MJ, et al. (1987) MAPMAKER: an interactive computer package for constructing primary genetic linkage maps of experimental and natural populations. Genomics 1: 174-181.

8. Turgeon B, Debuchy R, editors (2007) *Cochliobolus* and *Podospora*: Mechanisms of sex determination and the evolution of reproductive lifestyle. Washington, DC: ASM. p93-121 p.

9. Turgeon BG, Bohlmann H, Ciuffetti LM, Christiansen SK, Yang G, et al. (1993) Cloning and analysis of the mating type genes from *Cochliobolus heterostrophus*. Mol Gen Genet 238: 270-284.
