# Supplementary material for: Comparative Genome Structure, Secondary Metabolite, and Effector Coding Capacity across Cochliobolus Pathogens
Source: PLoS Genet. 2013 Jan 24;9(1):e1003233. doi: 10.1371/journal.pgen.1003233 (PMC3554632; doi:10.1371/journal.pgen.1003233)
Supplement: Figure S5 — Maximum likelihood tree of NRPS AMP-binding (AMP) domains identified using Augustus (A) and Genewise (B). RAxML using the RTREVF model with a gamma distribution was used to infer the maximum likelihood tree and bootstrap support was determined using the fast-bootstrap method with 1000 bootstrap replicates. See Materials and Methods. AMP domains are color-coded by species. Plain branches at the top of the tree are AMP domains from related adenylating enzymes (e.g., acyl CoA ligases, etc). AMP domains corresponding to the C. heterostrophus reference set (Figure 3, Table 4, Table S6,) are indicated on the right of each group. Bootstrap values above the branches. Certain additional AMP domains correspond to well-known metabolites from fungi outside the Cochliobolus and Setosphaeria strains. Gibberella fujikuroi FusS produces fusarin C, Fusarium heterosporum EqiS produces Equisetin, Aspergillus fumigatus GliP produces Gliotoxin, Leptosphaeria maculans SirP produces sirodesmin, Magnaporthe oryzae Ace1 produces an unknown metabolite involved in pathogenicity, Fusarium spp. Esyn1 produces enniatin, AAR, involved in lysine biosynthesis, Tolypocladium inflatum TiSimA produces cyclosporin, Penicillium spp., produces penicillin, δ-(L-α-aminoadipyl)-L-cysteine-D-valine (ACV), C. carbonum HTS1 produces HC-toxin, Hypocrea virens Tex1 produces peptaibols, Alternaria alternata AMT produces AMT toxin, Claviceps purpurea PS1(LPS1) is an NRPS that along with lysergic acid, produces an ergot alkaloid, Metarhizium anisopliae PesA unknown product, Epichloë festucae perA produces Peramine. (PDF) [file pgen.1003233.s005.pdf]

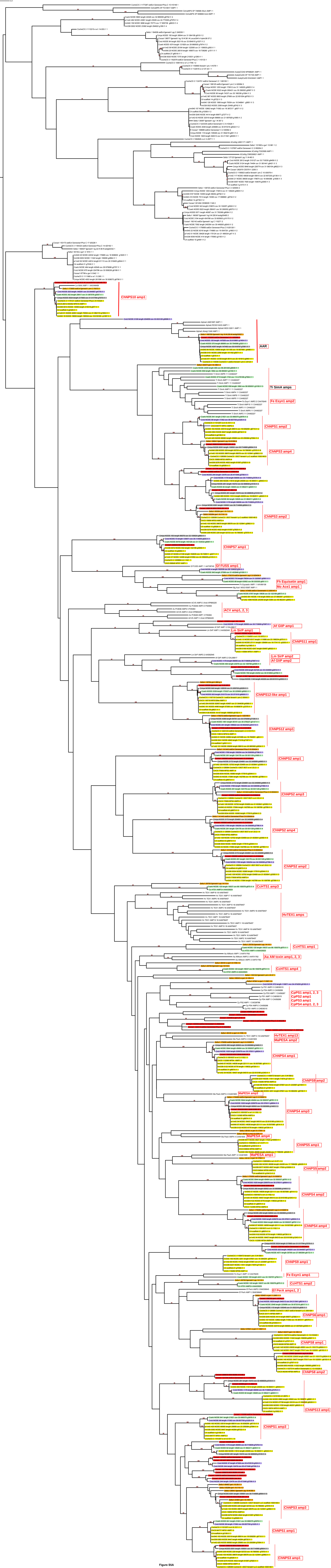

ChNPS10 amp1
AAR
Tl SimA amps
Fe Esyn1 amp2
ChNPS1 amp2
ChNPS3 amp4
ChNPS3 amp2
ChNPS7 amp1
GI FUS5 amp1
Fh Equisetin amp1
Mo Ace1 amp1
ACV amp1, 2, 3
Af GliP amp1
Lm SirP amp1
ChNPS11 amp1
Lm SirP amp2
Af GliP amp2
ChNPS12-like amp1
ChNPS12 amp1
ChNPS2 amp1
ChNPS2 amp3
ChNPS2 amp4
ChNPS2 amp2
ChCHTS1 amp3
HvTEX1 amps
ChCHTS1 amp1
Aa AM toxin amp1, 2
ChCHTS1 amp4
HvTEX1 amp13
MaPESA amp2
ChNPS4 amp1
ChNPS8 amp2
MaPESA amp3
ChNPS4 amp3
MaPESA amp4
ChNPS5 amp1
MaPESA amp1
ChNPS5 amp2
ChNPS4 amp2
ChNPS4 amp4
ChNPS9 amp1
Fe Esyn1 amp1
ChCHTS1 amp2
Ef PerA amp1, 2
ChNPS6 amp1
ChNPS8 amp1
ChNPS8 amp2
ChNPS13 amp1
ChNPS1 amp3
ChNPS3 amp3
ChNPS1 amp1
ChNPS3 amp1

Figure S5A

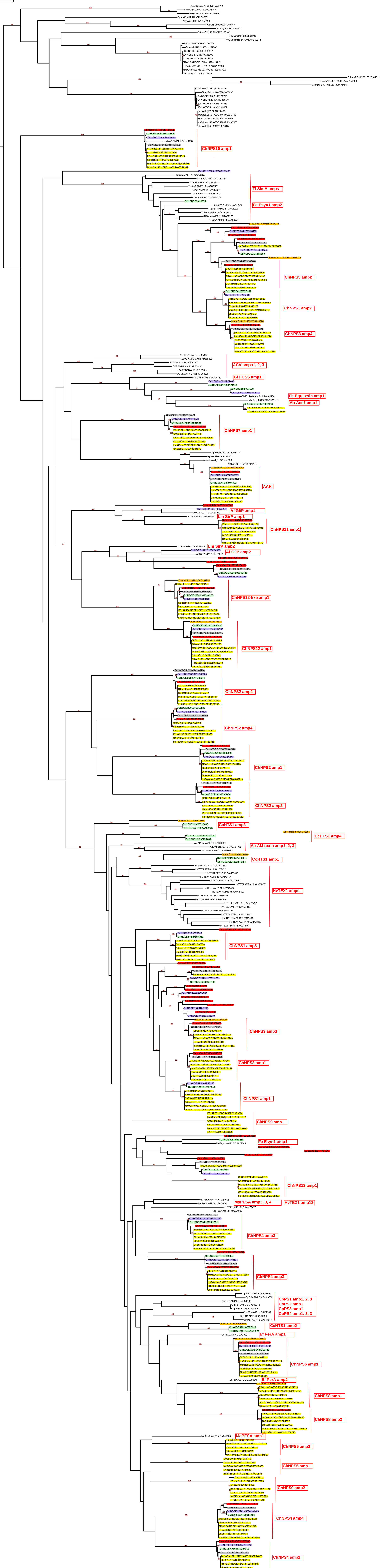

ChNPS10 amp1
Ti SimA amps
Fe Esyn1 amp2
ChNPS3 amp2
ChNPS1 amp2
ChNPS3 amp4
ACV amps1, 2, 3
Gf FUSS amp1
Fh Equisetin amp1
Mo Ace1 amp1
ChNPS7 amp1
AAR
Af GliP amp1
Lm SirP amp1
ChNPS11 amp1
Lm SirP amp2
Af GliP amp2
ChNPS12-like amp1
ChNPS12 amp1
ChNPS2 amp2
ChNPS2 amp4
ChNPS2 amp1
ChNPS2 amp3
CcHTS1 amp3
CcHTS1 amp4
Aa AM toxin amp1, 2, 3
CcHTS1 amp1
HvTEX1 amps
ChNPS1 amp3
ChNPS3 amp3
ChNPS3 amp1
ChNPS1 amp1
ChNPS9 amp1
Fe Esyn1 amp1
ChNPS13 amp1
MaPESA amp2, 3, 4
HvTEX1 amp13
ChNPS4 amp3
ChNPS4 amp3
CpPS1 amp1, 2, 3
CpPS2 amp1
CpPS3 amp1
CpPS4 amp1. 2, 3
CcHTS1 amp2
Ef PerA amp1
ChNPS6 amp1
Ef PerA amp2
ChNPS8 amp1
ChNPS8 amp2
MaPESA amp1
ChNPS5 amp2
ChNPS5 amp1
ChNPS9 amp2
ChNPS4 amp4
ChNPS4 amp2

Figure S5B
